# Rapid decreases in relative testes mass among monogamous birds but not in other vertebrates

**DOI:** 10.1111/ele.13431

**Published:** 2019-11-21

**Authors:** Joanna Baker, Stuart Humphries, Henry Ferguson‐Gow, Andrew Meade, Chris Venditti

**Affiliations:** ^1^ School of Biological Sciences University of Reading Reading RG6 6BX UK; ^2^ School of Life Sciences University of Lincoln Joseph Banks Laboratories Green Lane Lincoln LN6 7DL UK; ^3^ Department of Genetics, Evolution and Environment Centre for Biodiversity and Environment Research University College London Gower Street London WC1E 6BT UK

**Keywords:** Adaptation, evolutionary rates, relative testes mass, social mating system, sperm competition, vertebrates

## Abstract

Larger testes produce more sperm and therefore improve reproductive success in the face of sperm competition. Adaptation to social mating systems with relatively high and low sperm competition are therefore likely to have driven changes in relative testes size in opposing directions. Here, we combine the largest vertebrate testes mass dataset ever collected with phylogenetic approaches for measuring rates of morphological evolution to provide the first quantitative evidence for how relative testes mass has changed over time. We detect explosive radiations of testes mass diversity distributed throughout the vertebrate tree of life: bursts of rapid change have been frequent during vertebrate evolutionary history. In socially monogamous birds, there have been repeated rapid reductions in relative testes mass. We see no such pattern in other monogamous vertebrates; the prevalence of monogamy in birds may have increased opportunities for investment in alternative behaviours and physiologies allowing reduced investment in expensive testes.

## Graphical abstract

Explosive radiations of testes mass diversity have punctuated the vertebrate tree of life: bursts of rapid testes mass change have been frequent during vertebrate evolutionary history. In socially monogamous birds, there have been repeated rapid reductions in relative testes mass.

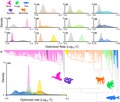

## Background

**Figure 1:**
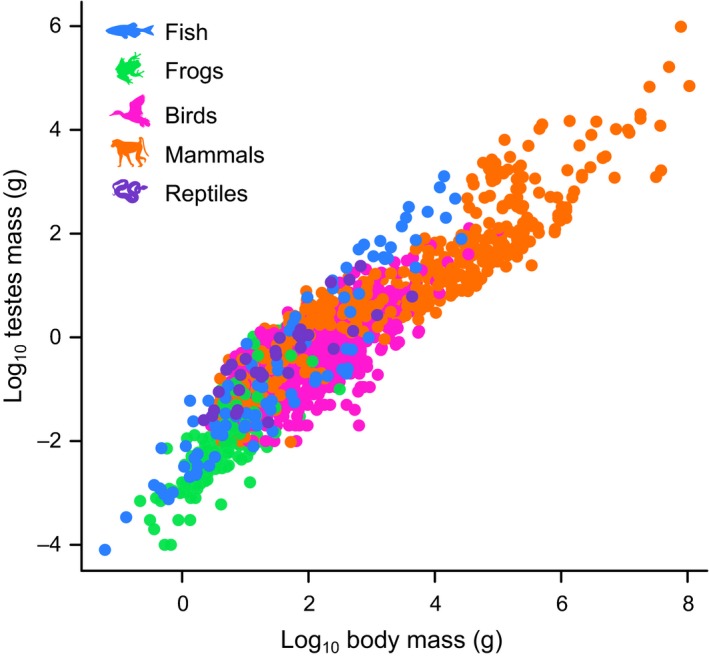
Testes mass and body mass for 1913 vertebrate species. Testes mass is highly variable even after accounting for body mass: in a maximum likelihood phylogenetic generalized least squares (GLS) regression model across this data, *r* ^2^ = 0.231. All silhouettes are taken from phylopic.org.

Testes mass is extremely variable across vertebrates, even after considering its association with body mass (e.g. MacLeod & MacLeod 2009, this variation is visualised in Figure 1). Many decades of research have shown that one of the most important factors explaining variation in relative testes size (including mass) among species is sperm competition. Sperm competition arises when sperm from multiple males compete for the fertilisation of a single female (Parker 1970). Increasing testes size is one way of improving male reproductive success in the presence of high levels of sperm competition (Parker *et al.* 1997; Parker & Pizzari 2010; Vahed & Parker 2012). Larger testes are likely to produce more sperm (Møller 1988, 1989; Stockley *et al.* 1997) which is a key determinant of competitive fertilisation success (Parker & Pizzari 2010). Accordingly, sperm competition has repeatedly been demonstrated to be linked to differences in testes sizes both within individual species (Hosken & Ward 2001; Schulte‐Hostedde & Millar 2004; Simmons & García‐González 2008) and amongst whole taxonomic groups including (but not limited to) butterflies (Gage 1994), bats (Hosken 1998) and frogs (Byrne *et al.* 2002) – reviewed in Parker *et al. *(1997).

The level of sperm competition a species faces can be approximated using social mating system (e.g. Harcourt *et al.* 1981; Pitcher *et al.* 2005). Socially polyandrous species – where females form social bonds with two or more males within a single breeding season – have ample opportunity to mate with multiple males and thus have relatively high levels of sperm competition (Smith 1984). In comparison, socially monogamous species form pair‐bonds between a single male and a single female that persist throughout an entire breeding season. Although extra‐pair copulations occur within socially monogamous species (e.g. DeWoody & Avise 2001; Griffith *et al.* 2002), compared with socially polyandrous species they have less opportunity for mating with multiple males and therefore lower levels of sperm competition. The amount of sperm competition faced by socially polygynous species, where a single male forms social bonds with multiple females within a single breeding season, may be different from both socially polyandrous and monogamous species (e.g. Hasselquist & Sherman 2001; Soulsbury 2010). There are two opposing predictions made for the level of sperm competition faced by socially polygynous species. Firstly, where males invest in increasing the number of socially‐bonded females, they are unlikely to be able to mate‐guard as effectively, potentially leading to increases in the level of sperm competition compared with social monogamy (Birkhead & Møller 1992). Secondly, female choice may lead to a reduced investment of females seeking or accepting extra‐pair copulations (Møller 1992; Hasselquist & Sherman 2001). This reduction in female investment in extra‐pair mating coupled with a reduced ability of male investment in extra‐pair mating owing to defense of his territory may actually decrease the amount of sperm competition faced by polygynous species (Hasselquist & Sherman 2001).

Here, in line with the expectations outlined above, we consider the following social mating systems separately: monogamy, polygyny, and social polyandry (including polygynandry, where multiple females and males form social bonds within a breeding season). In general, the testes of males belonging to social mating systems comprising multiple males are likely to be larger than those comprising only a single male. Such an expectation has been upheld in many groups spanning the vertebrate tree of life (e.g. Harcourt *et al.* 1981; Jennions & Passmore 1993; Parker *et al.* 1997; Pitcher *et al.* 2005), though reportedly not in all cases (e.g. Iossa *et al.* 2008).

We seek to provide the first comprehensive phylogenetic analysis of how testes mass diversity has evolved in combination with social mating system across vertebrates. We now have the opportunity to use phylogenetic approaches that simultaneously characterise the underlying testes‐body mass relationship whilst detecting rapid bursts in the rate of relative testes mass evolution (Venditti *et al.* 2011; Baker *et al.* 2016). Where the rate of evolution is faster, testes mass changes more than expected along an individual branch given the background rate of evolutionary change acting across all vertebrates and the amount of time that it has had to evolve (see Methods). Testes are energetically expensive to develop and maintain (e.g. Meerlo *et al.* 1997; Schulte‐Hostedde *et al.* 2005; Hayward & Gillooly 2011). Therefore, any rapid changes in testes mass along the branches of the vertebrate phylogenetic tree are likely to be a consequence of natural selection (most likely sexual selection imposed by sperm competition), reflecting periods of intense adaptive change (Baker *et al.* 2016; Baker & Venditti 2019).

We expect sperm competition to be a key driver of such intense adaptation in testes mass, and possibly may have even driven long term directional change in relative testes mass over millions of years. Directional trends in relative testes mass could occur in either direction. For example, social polyandry may have maintained high levels of sperm competition and thus exerted pressure for adaptive (i.e. rapid) increases in relative testes mass. On the other hand, in social mating systems with relatively lower levels of sperm competition it may have been beneficial to minimise investment in expensive reproductive tissues in favour of other expensive tissues such as brains (Aiello & Wheeler 1995; Pitnick *et al.* 2006) or alternative adaptations for improving reproductive success such as weapons, displays, or paternal care (e.g. Møller 2000; Lüpold *et al.* 2014; Buzatto *et al.* 2015; Dunn *et al.* 2015). In such cases, we might expect to see rapid relative testes mass reductions. Such bursts of directional rapid evolutionary change – in either direction – if repeated over millions of years along many branches of the vertebrate tree of life, could combine to give rise to sustained directional changes in relative testes mass over the last 400 million years of vertebrate evolutionary history (Baker *et al.* 2015). Thus, we might expect to see a trend towards larger relative testes mass in socially polyandrous species, and smaller relative testes mass in socially monogamous and polygynous species – with potential differences between the two.

## Material and methods

### Data and Phylogenetic tree

**Figure 2:**
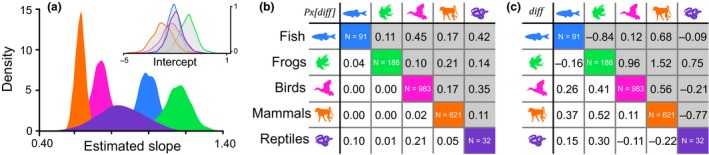
Testes mass allometry in vertebrates. (a) The posterior distribution of regression coefficients (slopes) inferred from our variable rates regression model estimating the relationship between testes mass and body mass for each of the five major vertebrate classes. Inset: The posterior distribution of intercepts for each group. Colours link to silhouettes in both (b) and (c) which show post‐hoc pairwise comparisons assessing the posterior distribution of the differences between each pair of estimated parameters. In (b) values represent the proportion of the posterior distribution of differences that cross zero, $P_{x\left[\mathrm{diff}\right]}$ and in (b) values represent mean differences. In both (b) and (c), the upper right half of the table shaded in grey shows values for intercepts and the lower left half shows values for slopes. The sample size for each group is shown along the diagonal. All silhouettes are taken from phylopic.org.

We collected testes mass (grams, g) and body mass (g) from the literature for 1913 vertebrate species matched to the time tree of life (Hedges *et al.* 2015). Sample sizes for each group are shown in Figure 2. We included only a single source per species in order to avoid conflicts among datasets; more detail on our data collection procedure can be found in the supplementary text. We log_10_‐transformed all testes masses and body masses (Figure 1).

We collected information on the social mating system from the literature, assigning a total of 1445 of the species for which we had testes mass to one of three possible states (monogamy, polygyny, polyandry/polygynandry). For birds, we excluded species where the social mating system is to be considered ‘cooperative’ as in many cases, group composition in terms of numbers of males and females was unspecified. For frogs, only coarse data were available and so the mating system of these species was collected as a dichotomous variable: monogamous/polygynous and polyandrous/polygynandrous. More details on how species were classified can be found in the supplementary text. Our total sample sizes for species with social mating system data were as follows: 63 fish, 169 frogs, 845 birds, and 358 mammals.

Our full protocol, reference list, and dataset are available in the online supplementary material (see Appendix S1 and Table S1).

### Rate heterogeneity

We detected variation in the rate of testes mass evolution after accounting for body mass using the *variable rates regression model* (Venditti *et al.* 2011; Baker *et al.* 2016). This Bayesian Markov chain Monte Carlo regression technique acts within a phylogenetic generalised least squares (GLS) framework to estimate the rate of evolution in the phylogenetically structured residual error of a regression model along the branches of a tree (Baker *et al.* 2016). The model simultaneously estimates an underlying Brownian motion process (background rate, $\sigma_{b}^{2}$) along with a set of rate scalars *r* defining branch‐wise shifts (identifying branches evolving faster (r>1) or slower (0≤r<1) than the background rate. The model then multiplies the original branch lengths (measured in time) by the corresponding *r* for each branch, resulting in a scaled phylogeny where longer branches (compared to their original length in time, r>1) indicate faster rates of morphological evolution, and shorter branches (0≤r<1) have slower rates. These branch‐specific scalars therefore optimise the fit of the phylogeny to the underlying background rate $\sigma_{b}^{2}$ given the inferred phenotypic change along each branch.

We used Bayes Factors (BF) to identify evidence for rate heterogeneity, calculated as $BF=-2\log_{e}\left[m_{1}/m_{0}\right],$ comparing the marginal likelihood of our variable rates model ($m_{1}$) to that of a model with a single underlying $\sigma_{b}^{2}$ ($m_{0}$). Marginal likelihoods were estimated using stepping‐stone sampling (Xie *et al.* 2010) implemented in BayesTraits (Pagel *et al.* 2004). For each of 200 stones, we ran 1 million iterations drawing values from a beta‐distribution (α = 0.40, β = 1) (Xie *et al.* 2010) and discarded the first 250 000 iterations as burn‐in. The variable rates model is implemented within a Markov Chain Monte Carlo framework, giving us a posterior distribution of estimated *r* and $\sigma_{b}^{2}$ *.* We visually checked all traces to ensure models were robust and had reached convergence. We ensured effective sample sizes of greater than 500 for all parameters, and results were replicated over multiple independent chains.

For each branch, we calculate an *optimised rate* $\left(\sigma_{v}^{2}=\sigma_{b}^{2}r\right)$. We then identified branches where $\sigma_{v}^{2}$ differs from the background rate $\left(\sigma_{b}^{2}\right).$ For a given branch, where *r > *1 in more than 50% of the posterior, we considered $\sigma_{v}^{2}\neq \sigma_{b}^{2};$this is where more than half of all iterations in the MCMC chain show an increase (or decrease where *r* < 1) in the rate of morphological evolution. All other branches were assumed to be evolving according to the background rate$\left(\sigma_{v}^{2}=\sigma_{b}^{2}\right)$. We then identified clades of 10 or more species across the vertebrate phylogeny that have inherited wholescale increases in the rate of relative testes mass evolution by comparing ancestor‐descendant branch pairs. These clades are termed *heritable rate shifts* and are defined on the basis of two criteria: (1) where a branch differs in rate ($\sigma_{v}^{2}$) from its ancestor in > 50% of the posterior distribution and (2) where all descendant branches inherit this new rate; i.e. all descendant branches do not differ in rate from the initial ancestral lineage. In this way, we define heritable rate shifts on the basis of an increase in variance throughout the entire clade – the clade has more variation about the regression line than would be expected given the underlying relationship between testes mass and body mass (Baker *et al.* 2016). Rate decreases are identified in the same way; in these cases, the clade would have a reduction in variation about the regression line. All branches where $\sigma_{v}^{2}\neq \sigma_{b}^{2}$are considered instances of rapid evolutionary change (or decelerated evolution, where *r* < 1).

### Characterising the testes‐body mass relationship

We identified rate heterogeneity using a bivariate regression between testes mass and body mass across all vertebrates (*N* = 1913). Metabolic theory predicts a simple linear relationship between testes mass and body mass with scaling differences among groups (Hayward & Gillooly 2011) much like that observed for other organs (Peters 1986). We therefore additionally ran a model estimating an interaction between taxonomic group and body mass as a fixed effect, allowing a different slope within each major clade (fish, frogs, birds, mammals, and reptiles).

We calculate the proportion of the posterior distribution of each regression parameter that crosses zero ($P_{x}$). Where *P*_x_ < 0.05, this means that less than five percent of the posterior distribution overlaps with zero, and we consider a variable to be substantially different from zero. To compare between parameters, we calculated the difference between each pair of parameters at each iteration and looked at the posterior distribution of differences. Where the proportion of this distribution crossing zero is less than 5% ($P_{x\left[\mathrm{diff}\right]}$ < 0.05), we consider two parameters to be different from one another.

### Directionality in testes mass evolution and the role of social mating system

Our method of detecting rate heterogeneity introduces meaningful variation into the branch lengths of a phylogeny, which makes it possible to study adaptive trends in trait evolution (Baker *et al.* 2015). Longer branches represent an increase in the rate of evolution arising from selective influences (Baker *et al.* 2016; Baker & Venditti 2019); they have experienced more relative testes mass change than would be expected given their length in time. The sum of all the rate‐scaled branches along the evolutionary path of a species (*path‐wise rates*) can therefore be used to measure the total amount of adaptive change that species has experienced during its history (Baker *et al.* 2015). We used this logic to determine whether there has been any long‐term adaptive trends in vertebrate relative testes mass evolution and whether they differ among species experiencing different levels of sperm competition.

In order to estimate whether there has been any adaptive directionality in relative testes mass evolution, we incorporated social mating system, path‐wise rate and an interaction between the two variables as additional explanatory factors into the testes‐body mass regression model. We term these our *trends analyses*. We only do this where we have 10 data points per parameter (Freckleton & Watkinson 2001) i.e. *N* ≥ 20 (we estimate a slope and intercept for the relationship between testes mass and path‐wise rate after accounting for shared ancestry and body mass). Sample sizes for individual mating systems are shown in Figure 4 and details are found in the supporting information. We have too few monogamous (*N* = 15) and polygynous fish (*N* = 8) to estimate two separate slopes, so for this group, we combined monogamy and polygyny into a single category (as in frogs). Too few data were available for reptiles and so these taxa were excluded from these analyses.

We performed all trends analyses within a maximum‐likelihood phylogenetic GLS framework (Pagel 1999). We used the median path‐wise rate as our predictor variable (but results do not qualitatively differ using the mean or mode). We assessed significance of parameters using standard *P*‐values and compare model fit using likelihood ratio (*D*) tests. All trends analyses were conducted on the median rate‐scaled phylogeny in order to account for differences in the amount of testes mass change expected owing to rate heterogeneity (Baker *et al.* 2015) and were limited to the data to the species for which we could collect mating system data (see above and electronic supplementary material). To account for multiple hypothesis testing (multiple categories of mating system within each group), we adjust *P*‐values using Bonferonni corrections.

Where the slope of the relationship between testes mass and median path‐wise rate was determined to be non‐significant, we ran an additional model that estimated the difference in the intercept of the testes‐body mass regression relationship for the mating system of each group. This was to determine whether there were significant differences in the average relative testes mass of species belonging to each social mating system – after accounting for phylogenetic shared ancestry and the rate of testes mass evolution.

### Code availability

All analyses in the present study were conducted using the freely available software BayesTraits v3.0, available at the following website: http://www.evolution.rdg.ac.uk/BayesTraitsV3/BayesTraitsV3.0.1.html.

## Results

**Figure 3:**
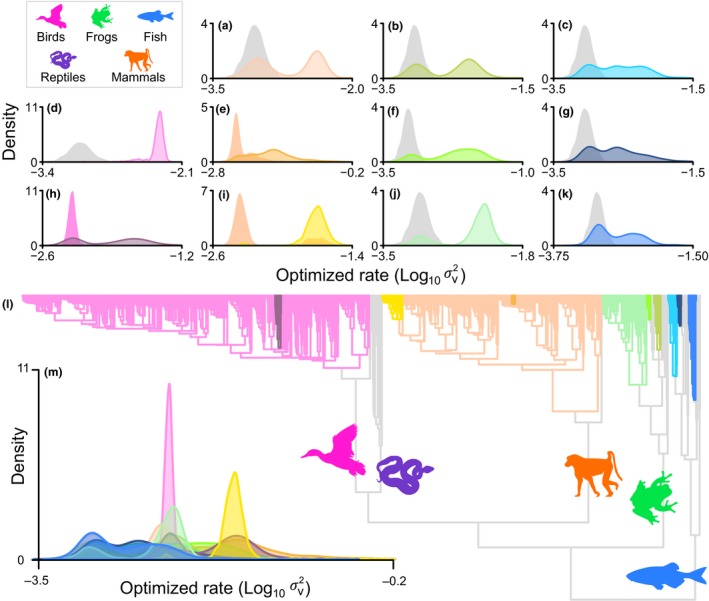
Heritable rate shifts in the rate of testes size evolution across the vertebrate phylogeny representing increases in testes mass diversity (each colour represents a unique shift). In (a–k), we show the distribution of $\sigma_{v}^{2}$ (optimized rate per branch) along the branch leading to each clade compared to the distribution of $\sigma_{v}^{2}$ along the immediately ancestral branch. The colours of each distribution correspond to the branch colours on the phylogeny (l). Silhouettes indicate the branch leading to each of the five major vertebrate clades and are taken from phylopic.org. We find heritable rate shifts in all major clades studied with the exception of reptiles: (a) Within mammals [Subclass Theria: *N* = 618]. (b) Within frogs [Superfamily Hyloidea + Family Myobatrachidae: *N* = 126] (c) Within fish [Order Perciformes: *N* = 26]. (d) Within birds [Neognathae: *N* = 979]. (e) Within mammals [Order Cetacea: *N* = 58]. (f) Within frogs [Genera Rana + Odorrana: *N* = 11]. (g) Within fish [Family Syngnathidae: *N* = 12]. (h) Within birds [Order Charardriiformes: *N* = 18]. (i) Within mammals [Genus Pseudomys: *N* = 10]. (j) Within frogs [Families Dicroglossidae and Rhacophoridae: *N* = 19]. (k) Within fish [Cyprinidae: *N* = 22]. For more detailed descriptions of each clade, see Table S2. In (m) distributions from (a–k) are shown on a single plot for comparison.

We find that testes mass evolution across vertebrates is best described by a model that allows a different relationship with body mass (i.e. a different allometric slope) for each of the five major classes we include (mammals, birds, fish, reptiles, and amphibians). There are substantial differences in slope among groups (Figure 2), with very little overlap in estimated parameters. With the exception of reptiles, less than 5% of the proportion of the posterior distribution of differences between each pair of slope parameters P*_x[diff]_* crosses zero. The magnitude of the difference varies from group to group and can be observed in Figure 2. Our results conform to theory predicting a simple linear relationship between testes mass and body mass, with variation amongst major groups (Hayward & Gillooly 2011). Deviations away from the underlying relationships arise in the form of rate heterogeneity (Figure 3).

Our results indicate rapid evolutionary change in both directions throughout the vertebrate tree of life (Figure 3). We find ‘very strong’ support (Raftery 1996) for rate heterogeneity compared to a model estimating only a single rate of evolution (BF = 660.86, Methods). The rate heterogeneity we identify arises in the form of 11 independent heritable rate shifts (Figure 3, Table S2) wherein a monophyletic group of 10 or more species inherits a single accelerated rate, resulting in a radiation of relative testes mass diversity (Methods). The heritable rate shifts we identify are distributed across the vertebrate phylogeny, occurring within all major vertebrate groups except reptiles (Figure 3). We additionally observe rate increases in 22 smaller clades (N ranging between 2 and 9) as well as 11 individual species (Table S2) – these increases in rate might indicate incipient heritable shifts. We observe only a single mean shift, where a change in the intercept of the regression relationship between testes mass and body mass manifests as a rate increase along an internal branch (Baker *et al.* 2016). This is observed within mammals, and more specifically, within rodents – along the branch leading to Australian hopping mice (genus *Notomys,* Table S2). The branch leading to *Notomys cervinus*, the fawn hopping mouse, was identified as a rate decrease – this species has experienced less change in testes mass than expected for its body mass and its branch length in time.

**Figure 4:**
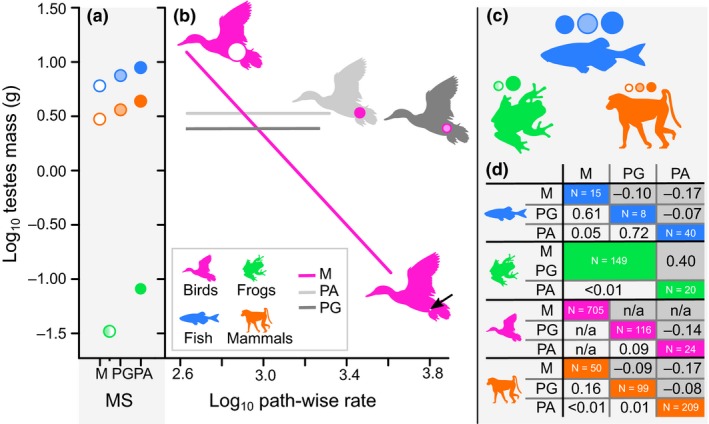
The effect of social mating system (MS) and the rate of morphological evolution on vertebrate testes sizes. (a) Differences between predicted testes mass (using the parameters of our regression model) of polyandrous/polygynandrous (PA, closed circles), polygynous (PG, lightly shaded circles), and monogamous (M, open circles) species given the average body mass within fish (523.60 g), frogs (11.46 g), and mammals (1548.82 g). (b) Rapid shifts in the rate of evolution give rise to an overwhelming tendency for testes mass reduction in monogamous birds (pink line). The relationship is non‐significant in birds of other social mating systems (grey lines and silhouettes). Differences in relative testes mass are visualised in (b) and (c); circles represent the predicted testes mass in proportion to the area of the animal silhouette assuming that it represents the average body mass. Each silhouette is identical in area. For an average‐sized (891.25 g) monogamous bird, we calculate the potential magnitude of adaptive testes reduction by predicting testes mass (white circles in (b)) given the smallest path‐wise rate (10.23 g) and the largest path‐wise rate (0.11 g, black arrow). Average body sizes are calculated using phylogenetic GLS models [4]. In (d) we show post‐hoc pairwise comparisons assessing the difference in average testes mass among different mating systems. The estimated intercept differences are shown by the upper right half of the table, shaded in grey (where a value is positive, it indicates that the column‐wise mating system is larger). The *P*‐value for this difference is shown in the lower left half of the table. Sample size for each group is shown along the diagonal. For frogs, we only have data on monandry/polyandry. For birds, pair‐wise differences from monogamous species are not comparable owing to the significant estimated slope. All silhouettes are taken from phylopic.org.

We find a significant negative relationship between testes mass and mean path‐wise rate for monogamous birds only (*P* < 0.01, Figure 4). There is no such significant relationship in birds belonging to any other social mating system. These results are supported using cross‐validation tests (see supplementary text for details).

In no other social mating system for any other group do we find a significant relationship between path‐wise rate and testes mass. However, it is significantly better to estimate separate intercepts allowing differences between the average testes mass of species belonging to different mating systems for each of these groups (frogs: *D* = 16.094, *P* < 0.01, d.f. = 1; fish: *D* = 6.07, *P* = 0.05, d.f. = 2; mammals: *D* = 11.97, *P* ≤ 0.01, d.f. = 2). In all three groups where we estimate mean differences between testes mass of species belonging to different social mating systems (fish, frogs, and mammals), we find that polygynandrous species have significantly larger testes than monogamous species (Figure 4). In mammals, polygynandrous species also have larger testes than polygynous species (Figure 4). No other comparison is significant (Figure 4). Correcting for multiple hypothesis testing (Bonferroni correction) does not alter our results, with one exception: statistical support for the differences in testes sizes between mating systems for fish is marginal, and is non‐significant after correcting for multiple hypothesis testing.

## Discussion

Our results highlight that social mating systems (and associated variation in sperm competition) are likely to have played a key role during the evolution of vertebrate testes. In line with expectations, we find that vertebrates with polyandrous mating systems tend to have significantly larger relative testes mass than monandrous vertebrates (Figure 4). Furthermore, several bursts of rapid testes mass change have punctuated vertebrate evolutionary history that are likely to have been linked to changes in reproductive biology. For example, we observe a heritable rate shift in the tree frog family Rhacophoridae (Roberts & Byrne 2011) (Figure 3j, Table S2) which has a high occurrence of polyandry (Roberts & Byrne 2011) and includes the grey foam‐nest tree frog (*Chiromantis xerampelina*), described as the most polyandrous of all vertebrate species (Byrne & Whiting 2011). We find rapid evolution leading to very tiny testes in a ‘classic’ example of a monogamous species (Ribble 1991), the California mouse *Peromyscus californicus* (nearly ten times the rate observed among other therian mammals, Table S2). The single mean shift we identify is a reduction in relative testes mass found within the monogamous hopping mouse genus *Notomys*, the remarkably small testes of which are thought to be linked with increased sperm efficiency (Breed & Jason 2000). A small group of pipefish – renowned for unusual reproductive biology (Kvarnemo & Simmons 2004) – have testes evolving at nearly twice the background rate (Figure 3, Table S2). Sex role reversal such as that observed among some pipefish (Berglund & Rosenqvist 2003) can lead to low levels of sperm competition even in species with high levels of polyandry (Rose *et al.* 2013) which might explain some of the rapid testes mass changes observed in this group. Although sex role‐reversal could lead to some of the other rapid evolutionary changes in relative testes masses that we observe, this phenomenon is relatively rare in vertebrates (Eens & Pinxten 2000) and thus our overall results are unlikely to be affected.

We find that there has only been very limited adaptive directional evolution in testes mass during the course of vertebrate evolutionary history. In birds only, rapid rates of testes mass change (i.e. where path‐wise rates are largest, see Methods) have overwhelmingly been towards smaller mass in the social mating system where sperm competition has been lowest: monogamy. We observe no such association in any other vertebrate group. This means that after an initial increase in size associated with the evolution of social polyandry, there has been no strong pressure *within* socially polyandrous species driving continued increases. This lack of continued adaptation suggests that there has not been increasing levels of sperm competition within groups over time**.** Additionally, non‐directionality in the evolution of testes mass of socially polyandrous vertebrates may be owing to the different types of polyandry observed in nature. Simultaneous polyandry, where a female produces a single brood after mating with multiple males would predict very high levels of sperm competition whereas sequential polyandry, where a female produces a brood with multiple males one after the other may predict relatively less – though sperm competition can still be very high in these species owing to sperm storage (Møller 1988; Oring *et al.* 1992).

One obvious reason for the observed differences between monogamous birds and other vertebrates is that flight restricts the available energy budget to birds (Butler & Bishop 2000). Any opportunity to reduce investment in expensive tissues (e.g. in the case of reduced sperm competition) may have been advantageous in such a scenario. However, we find no significant directionality in testes mass change along the branches of bats – the only other group of flighted vertebrates (*N* = 49, *P* = 0.71), so this explanation seems unlikely. An alternative explanation is the overall prevalence of monogamy in birds. Over 75% of all bird species are socially monogamous (Mock & Fujioka 1990; Dunn *et al.* 2001; Lukas & Clutton‐Brock 2013), and although social monogamy does exist in other vertebrates (Bull 2000; Whiteman & Côté 2004; Lukas & Clutton‐Brock 2013), it tends to be much rarer (Mock & Fujioka 1990; Bull 2000). Frogs also have high levels of monogamy (Liao *et al.* 2011) (88% in our dataset, Table S1), but most species are external fertilizers (Beck 1998), which imposes a unique set of selection pressures on testes compared with species with internal fertilisation (Parker 2014). The prevalence of single‐partner mating systems in combination with internal fertilisation in birds may have increased opportunity for the evolution of diverse behaviours and morphologies where investment in testes mass are less important (Parker 2014; Parker 2016). Sexual traits used for mate acquisition such as ornamentation or weapons have been shown to be more important investments than testes mass in several animal groups (Simmons & Emlen 2006; Fitzpatrick *et al.* 2012; Buzatto *et al.* 2015; Dines *et al.* 2015; Dunn *et al.* 2015) – although these tend to be associated with polygynous or lekking mating systems (Møller & Pomiankowski 1993; Savalli 1995). It therefore warrants further investigation to reveal whether any costly traits in particular are more prevalent in monogamous birds than those of other mating systems.

In general, a negative relationship between testes mass and its rate of evolution such as the one we find here for monogamous birds could imply a trade‐off between testes and other behaviours (see above). Trade‐offs between expensive organs like testes, brains, and guts are predicted by the expensive‐tissue hypothesis (Aiello & Wheeler 1995; Isler & van Schaik 2006) although there has been little evidence for this in testes (e.g. Schillaci 2006; Lemaître *et al.* 2009; Bordes *et al.* 2011; Kelley *et al.* 2014). However, at least across species, it may be difficult to observe trade‐offs owing to the numerous potential morphologies, physiologies, and behaviours that have the potential to trade‐off with testes at macroevolutionary scales (Lüpold *et al.* 2014; Somjee *et al.* 2018). An alternative explanation for such a negative relationship might be a continued reduction in the level of sperm competition (by some unknown mechanism) observed in monogamous species over time. Whilst an interesting concept – it implies increased specialisation to monogamous mating systems – this remains difficult to test in the face of present data. It is also hard to imagine that the level of sperm competition has continually decreased in monogamous birds over time when we consider the fact that there is variation in the level of sperm competition faced by monogamous species (Griffith *et al.* 2002; Ophir *et al.* 2008; Biagolini‐Jr *et al.* 2017).

The fact that monogamous species are increasingly shown to face sperm competition and vary in their level of extra‐pair paternity has led to a general acceptance that social and genetic mating systems may not directly correlate among different animal groups (DeWoody & Avise 2001; Griffith *et al.* 2002; Clutton‐Brock & Isvaran 2006; Isvaran & Clutton‐Brock 2006; Ophir *et al.* 2008; Biagolini‐Jr *et al.* 2017). An explicit empirical link between sperm competition and social mating system across vertebrates is desirable, though we currently lack the data to show this at large scales. This is owing to the fact most genetic data measures extra‐pair paternity (EPP) (e.g. Griffith *et al.* 2002; Biagolini‐Jr *et al.* 2017) which is not clearly comparable to paternity within social systems comprising multiple partners. The proportion of offspring sired by a male outside a social group (i.e. extra‐group paternity) (e.g. Westneat & Stewart 2003), is not comparable to EPP and actually tells us very little in terms of sperm competition – it would be better to measure the number of offspring fathered by the non‐dominant male (e.g. Møller & Briskie 1995). Per‐brood measures of multiple‐paternity such as number of sires per brood (Rowley *et al.* 2018) or the frequency of broods with mixed paternity (Taylor *et al.* 2014; Biagolini‐Jr *et al.* 2017; Rowley *et al.* 2018) can also give us an indication of sperm competition but are not without their problems. For example, the frequency of extra‐pair copulations does not correlate with the frequency of extra‐pair fertilisation in pair‐bonded species (Birkhead & Møller 1995) and it is unclear whether “mixed paternity” can be measurable or meaningful in species which routinely produce only a single offspring per clutch/brood. In any case, most studies identify variation in multiple‐paternity within socially monogamous species (reviewed in Griffith *et al.* 2002) – but this amounts to, on average, only 11% of all offspring being sired by a male that is not the social parent (Griffith *et al.* 2002), at least within birds.

Social mating system is still a reasonable proxy for the *relative* amount of sperm competition faced by species. Social monogamy clearly does not eliminate sperm competition but rather there is less scope for sperm competition compared with social polyandry. This has been shown in mammals, where socially monogamous species have significantly lower multiple paternity rates than those with multi‐male social systems (Soulsbury 2010). Sociality and mating system are also significantly linked to the occurrence of multiple‐mating by female birds (Møller & Birkhead 1993). Distinct social mating systems have also been maintained in an evolutionary sense and are repeatedly linked to testes size in many animal groups (e.g. Harcourt *et al.* 1981; Jennions & Passmore 1993; Parker *et al.* 1997; Pitcher *et al.* 2005) (also supported here across vertebrates, Figure 4). The fact that there is also an association between different genetic measures of multiple paternity and testes size across vertebrates (e.g. Møller & Briskie 1995; Ramm *et al.* 2005; Rowley *et al.* 2018) makes it almost impossible to imagine a scenario where social mating system provides no information on the relative amount of sperm competition faced by species.

Sperm competition clearly has an important role in driving changes in testes mass (Harcourt *et al.* 1981; Gage 1994; Møller & Briskie 1995; Hosken 1997; Parker *et al.* 1997; Birkhead & Møller 1998) (Figure 4), but exactly how changes in social mating system can explain the radiations of testes mass diversity that we see across vertebrates (Figure 3) remains unclear, and warrants further investigation. We hope that our results will inspire researchers to investigate what factors might have been driving the strong selection on relative testes mass that manifest as rapid bursts of evolutionary change during the course of vertebrate history. For example, as more data on both testes mass and mating systems become available in other groups, it may be possible to reveal nuances in the evolution of relative testes mass that are currently otherwise impossible. Factors such as geography, dispersion, mating rates, or migratory behaviour (Dunn *et al.* 2001; Pitcher *et al.* 2005; Vahed & Parker 2012), etc. may also have played key roles in driving bursts of testes mass change.

Many radiations of testes mass diversity (Figure 3, Table S2) have punctuated the last 400 million years of vertebrate evolutionary history. These radiations reveal clades in the vertebrate phylogeny where there has been intense adaptive change in testes mass. However, in most vertebrates, these adaptive bursts of testes mass evolution have not led to any sustained directional changes (Figure 4). In socially monogamous birds only, we observe adaptive reductions in testes mass that almost certainly arise from a combination of different factors and trade‐offs. One key outcome of our analysis is that we highlight a novel opportunity to reveal historical trends in traits after considering the effect of other factors such as body mass. Soft tissues like testes are often preserved poorly in the fossil record (Brusatte 2012), and we reveal patterns and processes of evolution that occurred deep in time that may otherwise have been impossible to detect.

## Authorship

All authors contributed to this work, including the writing of the manuscript.
