# Supplementary material for: Rapid decreases in relative testes mass among monogamous birds but not in other vertebrates
Source: Ecol Lett. 2019 Nov 21;23(2):283–92. doi: 10.1111/ele.13431 (PMC6973093; doi:10.1111/ele.13431)
Supplement: Supplementary file 1 [file ELE-23-283-s001.docx]

| **Table S1:** Dataset used for all analyses. Both testes mass (TM) and body mass (BM) are provided in units of log_10_ grams. Social mating systems (MS) are abbreviated as follows: C (cooperative), M (monogamous), PG (polygynous), PA (polyandrous), PGA (polygynandrous). | | | | | | | |
| --- | --- | --- | --- | --- | --- | --- | --- |
| **Species** | **TM** | **Ref** | **BM** | **Ref** | **Notes** | **MS** | **Ref** |
| **Fish** | | | | | | | |
| Coregonus_migratorius | 1.875 | Stockley 1997 | 3.699 | FishBase | Original data is for Coregonus_peled - taxa included by genus matching. |  |  |
| Doryrhamphus_excisus | -4.097 | Kvarnemo 2004 | -1.222 | Kvarnemo 2004 | Original data is for Doryrhamphus_negrosensis - taxa included by genus matching. |  |  |
| Gila_conspersa | -0.538 | Pyron 2013 | 1.101 | Pyron 2013 | Original data is for Gila_atraria - taxa included by genus matching. | PA/PGA | Pyron 2013 |
| Hippichthys_penicillus | -3.027 | Kvarnemo 2004 | -0.284 | Kvarnemo 2004 | Original data is for Hippichthys_heptagonus - taxa included by genus matching. | M | Fishbase |
| Hippocampus_kelloggi | -1.738 | Kvarnemo 2004 | 0.985 | Kvarnemo 2004 | Original data is for Hippocampus_angustus - taxa included by genus matching. | M | Fishbase |
| Lythrurus_roseipinnis | -2.097 | Pyron 2013 | 0.058 | Pyron 2013 | Original data is for Lythrurus_bellus - taxa included by genus matching. | PA/PGA | Pyron 2013 |
| Melanotaenia_sp._AC-2011 | -2.493 | Hayward 2011 | 0.032 | Hayward 2011 | Original data is for Melanotaenia_eachamensis - taxa included by genus matching. |  |  |
| Oreochromis_andersonii | 1.735 | Stockley 1997 | 3.303 | FishBase | Original data is for Oreochromis_aureus - taxa included by genus matching. | M | Stockley 1997 |
| Solegnathus_hardwickii | -1.253 | Kvarnemo 2004 | 1.778 | Kvarnemo 2004 | Original data is for Solegnathus_spinosissimus - taxa included by genus matching. |  |  |
| Tilapia_guinasana | 0.130 | Liao 2018 | 1.699 | Liao 2018 | Original data is for Tilapia_zillii - taxa included by genus matching. | M | Stockley 1997 |
| Acipenser_baerii | 3.105 | Liao 2018 | 4.146 | FishBase |  | PA/PGA | Liao 2018 |
| Acipenser_gueldenstaedtii | 2.140 | Liao 2018 | 3.477 | Kvarnemo 2004 |  | PA/PGA | Stockley 1997; Liao 2018 |
| Acipenser_persicus | 2.892 | Liao 2018 | 4.079 | Pyron 2013 |  | PA/PGA | Liao 2018 |
| Acipenser_ruthenus | 1.563 | Liao 2018 | 3.027 | Kvarnemo 2004 |  | PA/PGA | Liao 2018 |
| Acipenser_stellatus | 2.307 | Liao 2018 | 3.544 | Kvarnemo 2004 |  | PA/PGA | Liao 2018 |
| Acipenser_transmontanus | 2.674 | Liao 2018 | 4.322 | Pyron 2013 |  | PA/PGA | Stockley 1997; Liao 2018 |
| Agosia_chrysogaster | -1.469 | Pyron 2013 | 0.686 | Hayward 2011 |  | PA/PGA | Pyron 2013 |
| Barbus_barbus | 1.096 | Liao 2018 | 2.380 | FishBase |  | PA/PGA | Liao 2018 |
| Bodianus_rufus | -0.745 | Hayward 2011 | 2.301 | Kvarnemo 2004 |  | PG | Fishbase |
| Campostoma_ornatum | -1.854 | Pyron 2013 | 0.544 | Liao 2018 |  | PA/PGA | Fishbase; Pyron 2013 |
| Carassius_carassius | 0.770 | Liao 2018 | 2.566 | Liao 2018 |  | PA/PGA | Fishbase; Stockley 1997; Liao 2018 |
| Clarias_gariepinus | 0.840 | Eyo 2014 | 2.800 | Liao 2018 |  | PA/PGA | Fishbase |
| Clepticus_parrae | -0.796 | Hayward 2011 | 2.125 | Liao 2018 |  |  |  |
| Clinostomus_funduloides | -1.081 | Pyron 2013 | 0.664 | Liao 2018 |  | PA/PGA | Pyron 2013 |
| Corythoichthys_intestinalis | -2.690 | Kvarnemo 2004 | 0.121 | Liao 2018 |  | M | Fishbase |
| Cryptotomus_roseus | -1.699 | Hayward 2011 | 0.688 | Liao 2018 |  | M | Fishbase |
| Ctenopharyngodon_idella | 1.348 | Liao 2018 | 3.696 | Pyron 2013 |  | PA/PGA | Stockley 1997; Liao 2018 |
| Cyprinella_lutrensis | -1.699 | Pyron 2013 | 0.523 | Liao 2018 |  | PA/PGA | Pyron 2013 |
| Cyprinus_carpio | 1.857 | Liao 2018 | 3.137 | Hayward 2011 |  | PA/PGA | Stockley 1997; Liao 2018 |
| Dunckerocampus_dactyliophorus | -2.600 | Kvarnemo 2004 | 0.230 | Pyron 2013 |  |  |  |
| Entelurus_aequoreus | -2.910 | Kvarnemo 2004 | -0.357 | Liao 2018 |  |  |  |
| Esox_lucius | 1.544 | Liao 2018 | 3.189 | Eyo 2014 |  | PA/PGA | Stockley 1997; Liao 2018 |
| Halicampus_grayi | -2.251 | Kvarnemo 2004 | 0.246 | Hayward 2011 |  |  |  |
| Halichoeres_bivittatus | -0.959 | Hayward 2011 | 1.271 | Pyron 2013 |  |  |  |
| Halichoeres_garnoti | -0.222 | Hayward 2011 | 2.602 | Kvarnemo 2004 |  | M | Fishbase |
| Halichoeres_maculipinna | -1.398 | Hayward 2011 | 1.398 | Hayward 2011 |  |  |  |
| Halichoeres_pictus | -1.699 | Hayward 2011 | 1.187 | Liao 2018 |  |  |  |
| Halichoeres_poeyi | -1.222 | Hayward 2011 | 1.416 | Pyron 2013 |  |  |  |
| Halichoeres_radiatus | -0.770 | Hayward 2011 | 2.117 | Liao 2018 |  |  |  |
| Haliichthys_taeniophorus | -1.460 | Kvarnemo 2004 | 1.013 | Kvarnemo 2004 |  |  |  |
| Hesperoleucus_symmetricus | -1.620 | Pyron 2013 | 0.176 | Kvarnemo 2004 |  | PA/PGA | Pyron 2013 |
| Hippocampus_erectus | -2.102 | Kvarnemo 2004 | 1.127 | Liao 2018 |  |  |  |
| Ictalurus_punctatus | 1.897 | Stockley 1997 | 4.420 | Kvarnemo 2004 |  | M | Stockley 1997 |
| Labeo_rohita | 1.525 | Liao 2018 | 3.255 | Hayward 2011 |  |  |  |
| Lavinia_exilicauda | -0.932 | Pyron 2013 | 0.726 | Hayward 2011 |  | PA/PGA | Pyron 2013 |
| Lepidomeda_vittata | -1.222 | Pyron 2013 | 0.125 | Hayward 2011 |  | PA/PGA | Pyron 2013 |
| Lepomis_macrochirus | 0.260 | Liao 2018 | 1.754 | Hayward 2011 |  | PA/PGA | Stockley 1997; Liao 2018 |
| Microphis_brachyurus | -2.650 | Kvarnemo 2004 | 0.217 | Hayward 2011 |  |  |  |
| Morone_chrysops | 0.944 | Liao 2018 | 2.387 | Hayward 2011 |  | PA/PGA | Liao 2018 |
| Nerophis_ophidion | -2.848 | Kvarnemo 2004 | -0.444 | Kvarnemo 2004 |  | PA/PGA | Fishbase |
| Nocomis_asper | -0.636 | Pyron 2013 | 1.519 | Pyron 2013 |  | M | Pyron 2013 |
| Nocomis_biguttatus | -0.496 | Pyron 2013 | 1.504 | Kvarnemo 2004 |  | PA/PGA | Fishbase |
| Nocomis_leptocephalus | -0.094 | Pyron 2013 | 1.952 | FishBase |  | M | Pyron 2013 |
| Nocomis_micropogon | -1.886 | Pyron 2013 | 0.637 | Liao 2018 |  | M | Pyron 2013 |
| Notemigonus_crysoleucas | -1.222 | Pyron 2013 | 0.416 | Pyron 2013 |  | PA/PGA | Pyron 2013 |
| Oncorhynchus_kisutch | 2.513 | Liao 2018 | 3.588 | Pyron 2013 |  | PA/PGA | Stockley 1997; Liao 2018 |
| Oncorhynchus_mykiss | 1.788 | Liao 2018 | 2.879 | Liao 2018 |  | PA/PGA | Stockley 1997; Liao 2018 |
| Oncorhynchus_tshawytscha | 1.695 | Liao 2018 | 2.794 | Kvarnemo 2004 |  | PA/PGA | Stockley 1997; Liao 2018 |
| Perca_fluviatilis | 0.771 | Liao 2018 | 1.982 | Liao 2018 |  | PA/PGA | Fishbase; Stockley 1997; Liao 2018 |
| Phyllopteryx_taeniolatus | -1.500 | Kvarnemo 2004 | 1.158 | Kvarnemo 2004 |  |  |  |
| Pimephales_promelas | -1.538 | Pyron 2013 | 0.462 | Pyron 2013 |  | PA/PGA | Fishbase |
| Polyodon_spathula | 2.305 | Liao 2018 | 4.175 | Pyron 2013 |  | PA/PGA | Stockley 1997; Liao 2018 |
| Pseudocrenilabrus_multicolor | -1.523 | Liao 2018 | 0.851 | Pyron 2013 |  | M | Liao 2018 |
| Richardsonius_balteatus | -1.167 | Pyron 2013 | 0.686 | Pyron 2013 |  | PA/PGA | Pyron 2013 |
| Rutilus_rutilus | 0.398 | Liao 2018 | 1.792 | Pyron 2013 |  | PA/PGA | Liao 2018 |
| Salmo_salar | 2.420 | Liao 2018 | 3.886 | Liao 2018 |  | PA/PGA | Stockley 1997; Liao 2018 |
| Salmo_trutta | 1.342 | Liao 2018 | 2.602 | Liao 2018 |  | PA/PGA | Stockley 1997; Liao 2018 |
| Sarotherodon_aff._galilaeus_mudfeeder | 1.510 | Stockley 1997 | 3.209 | Liao 2018 |  | M | Stockley 1997 |
| Scardinius_erythrophthalmus | -0.125 | Liao 2018 | 1.021 | Liao 2018 |  | PA/PGA | Stockley 1997; Liao 2018 |
| Scarus_iseri | -1.097 | Hayward 2011 | 1.727 | Kvarnemo 2004 |  | PG | Fishbase |
| Scarus_taeniopterus | -0.699 | Hayward 2011 | 2.602 | Pyron 2013 |  | M | Fishbase |
| Scarus_vetula | -0.004 | Hayward 2011 | 2.954 | Liao 2018 |  | PG | Fishbase |
| Solegnathus_guentheri | -1.810 | Kvarnemo 2004 | 1.431 | Liao 2018 |  |  |  |
| Sparisoma_atomarium | -1.699 | Hayward 2011 | 1.000 | Pyron 2013 |  | PG | Fishbase |
| Sparisoma_aurofrenatum | -0.854 | Hayward 2011 | 2.105 | Liao 2018 |  | PG | Fishbase |
| Sparisoma_chrysopterum | -0.620 | Hayward 2011 | 2.535 | Liao 2018 |  | PG | Fishbase |
| Sparisoma_radians | -0.921 | Hayward 2011 | 1.198 | Liao 2018 |  |  |  |
| Sparisoma_rubripinne | -0.237 | Hayward 2011 | 2.684 | FishBase |  | PG | Fishbase |
| Sparisoma_viride | -0.602 | Hayward 2011 | 2.620 | Liao 2018 |  | PG | Fishbase |
| Stigmatopora_argus | -2.474 | Kvarnemo 2004 | 0.246 | Hayward 2011 |  |  |  |
| Stigmatopora_nigra | -3.469 | Kvarnemo 2004 | -0.886 | Hayward 2011 |  |  |  |
| Syngnathus_abaster | -2.932 | Kvarnemo 2004 | -0.337 | Hayward 2011 |  |  |  |
| Syngnathus_acus | -1.281 | Kvarnemo 2004 | 1.332 | Kvarnemo 2004 |  |  |  |
| Syngnathus_floridae | -2.991 | Kvarnemo 2004 | -0.149 | Hayward 2011 |  |  |  |
| Syngnathus_fuscus | -2.331 | Kvarnemo 2004 | 0.196 | Hayward 2011 |  |  |  |
| Syngnathus_rostellatus | -2.137 | Kvarnemo 2004 | -0.328 | Hayward 2011 |  |  |  |
| Syngnathus_scovelli | -3.119 | Kvarnemo 2004 | -0.215 | Hayward 2011 |  | PA/PGA | Fishbase |
| Syngnathus_typhle | -2.476 | Kvarnemo 2004 | 0.428 | Hayward 2011 |  | M | Stockley 1997 |
| Thalassoma_bifasciatum | -1.523 | Hayward 2011 | 1.135 | Hayward 2011 |  |  |  |
| Tinca_tinca | 0.489 | Liao 2018 | 2.662 | Kvarnemo 2004 |  | PA/PGA | Stockley 1997; Liao 2018 |
| Vanacampus_poecilolaemus | -2.307 | Kvarnemo 2004 | 0.517 | Kvarnemo 2004 |  | PA/PGA | Fishbase |
| **Frogs** | | | | | | | |
| Physalaemus_cuvieri | -2.796 | Prado 2003 | 0.000 | Kvarnemo 2004 | Original data is for Physalaemus_crombiei - taxa included by genus matching. | M/PG | Prado 2003 |
| Kaloula_conjuncta | -1.896 | Zheng 2014 | 0.740 | Kvarnemo 2004 | Original data is for Kaloula_verrucosa - taxa included by genus matching. | M/PG | Zeng 2014 |
| Brachytarsophrys_feae | 0.020 | Zheng 2014 | 2.004 | Kvarnemo 2004 | Original data is for Brachytarsophrys_chuannanensis - taxa included by genus matching. | PA/PGA | Zeng 2014 |
| Babina_chapaensis | -1.886 | Liao 2018 | 0.964 | Kvarnemo 2004 | Original data is for Babina_daunchina - taxa included by genus matching. | M/PG | Liao 2018 |
| Megophrys_nasuta | -1.524 | Liao 2018 | 1.863 | Kvarnemo 2004 | Original data is for Megophrys_shapingensis - taxa included by genus matching. | M/PG | Zeng 2014 |
| Adelotus_brevis | -2.208 | Byrne 2002 | 0.734 | Kvarnemo 2004 |  | M/PG | Byrne 2002 |
| Amietophrynus_garmani | -1.256 | Prado 2003 | 1.630 | Kvarnemo 2004 |  | M/PG | Prado 2003 |
| Amietophrynus_gutturalis | -0.926 | Prado 2003 | 1.588 | Hayward 2011 |  | M/PG | Prado 2003 |
| Amietophrynus_maculatus | -1.428 | Prado 2003 | 0.991 | Liao 2018 |  | M/PG | Prado 2003 |
| Amolops_lifanensis | -1.349 | Zheng 2014 | 1.157 | Kvarnemo 2004 |  | M/PG | Zeng 2014 |
| Amolops_mantzorum | -1.646 | Zheng 2014 | 1.240 | Zheng 2014 |  | M/PG | Zeng 2014 |
| Assa_darlingtoni | -3.097 | Byrne 2002 | -0.412 | Byrne 2002 |  | M/PG | Byrne 2002 |
| Babina_pleuraden | -1.588 | Zheng 2014 | 0.982 | Zheng 2014 |  | M/PG | Zeng 2014 |
| Buergeria_buergeri | -1.674 | Prado 2003 | 0.613 | Prado 2003 |  | M/PG | Prado 2003 |
| Buergeria_japonica | -2.367 | Prado 2003 | 0.255 | Prado 2003 |  | M/PG | Prado 2003 |
| Bufo_gargarizans | -1.461 | Zheng 2014 | 1.539 | Zheng 2014 |  | M/PG | Zeng 2014 |
| Bufo_japonicus | -0.479 | Prado 2003 | 1.974 | Prado 2003 |  | M/PG | Prado 2003 |
| Bufo_tibetanus | -1.020 | Zheng 2014 | 1.307 | Zheng 2014 |  | M/PG | Zeng 2014 |
| Bufo_torrenticola | -0.514 | Kusano 1991 | 1.990 | Kusano 1991 |  | M/PG | Kusano 1991 |
| Chiromantis_xerampelina | 0.013 | Prado 2003 | 1.127 | Prado 2003 |  | PA/PGA | Prado 2003 |
| Crinia_deserticola | -3.155 | Byrne 2002 | -0.665 | Byrne 2002 |  | M/PG | Byrne 2002 |
| Crinia_parinsignifera | -4.000 | Byrne 2002 | -0.178 | Byrne 2002 |  | M/PG | Byrne 2002 |
| Crinia_riparia | -3.155 | Byrne 2002 | -0.339 | Byrne 2002 |  | M/PG | Byrne 2002 |
| Crinia_signifera | -3.699 | Byrne 2002 | -0.443 | Byrne 2002 |  | M/PG | Byrne 2002 |
| Crinia_tinnula | -3.523 | Byrne 2002 | -0.509 | Byrne 2002 |  | M/PG | Byrne 2002 |
| Cyclorana_alboguttata | -2.102 | Byrne 2002 | 1.054 | Byrne 2002 |  | M/PG | Byrne 2002 |
| Cyclorana_australis | -1.067 | Byrne 2002 | 1.405 | Byrne 2002 |  | M/PG | Byrne 2002 |
| Cyclorana_brevipes | -1.395 | Byrne 2002 | 0.849 | Byrne 2002 |  |  |  |
| Cyclorana_cryptotis | -2.222 | Byrne 2002 | 0.717 | Byrne 2002 |  | M/PG | Byrne 2002 |
| Cyclorana_cultripes | -2.149 | Byrne 2002 | 0.708 | Byrne 2002 |  | M/PG | Byrne 2002 |
| Cyclorana_longipes | -1.023 | Byrne 2002 | 0.607 | Byrne 2002 |  | M/PG | Byrne 2002 |
| Cyclorana_maculosa | -1.445 | Byrne 2002 | 0.856 | Byrne 2002 |  |  |  |
| Cyclorana_maini | -2.523 | Byrne 2002 | 0.630 | Byrne 2002 |  | M/PG | Byrne 2002 |
| Cyclorana_manya | -3.097 | Byrne 2002 | 0.211 | Byrne 2002 |  | M/PG | Byrne 2002 |
| Cyclorana_novaehollandiae | -1.256 | Byrne 2002 | 1.516 | Byrne 2002 |  | M/PG | Byrne 2002 |
| Cyclorana_platycephala | -1.742 | Byrne 2002 | 1.055 | Byrne 2002 |  | M/PG | Byrne 2002 |
| Cyclorana_vagitus | -1.903 | Byrne 2002 | 0.875 | Byrne 2002 |  |  |  |
| Cyclorana_verrucosa | -2.357 | Byrne 2002 | 0.940 | Byrne 2002 |  | M/PG | Byrne 2002 |
| Duttaphrynus_melanostictus | -1.676 | Liao 2018 | 1.309 | Liao 2018 |  | M/PG | Zeng 2014 |
| Eupemphix_nattereri | -1.770 | Prado 2003 | 0.944 | Prado 2003 |  | M/PG | Prado 2003 |
| Geocrinia_victoriana | -1.886 | Byrne 2002 | 0.197 | Byrne 2002 |  | M/PG | Byrne 2002 |
| Glandirana_rugosa | -1.834 | Kusano 1991 | 0.828 | Kusano 1991 |  | M/PG | Kusano 1991 |
| Heleioporus_australiacus | -1.126 | Byrne 2002 | 1.753 | Byrne 2002 |  | M/PG | Byrne 2002 |
| Hyla_annectans | -1.309 | Zheng 2014 | 0.544 | Zheng 2014 |  | PA/PGA | Zeng 2014 |
| Hyla_japonica | -2.167 | Prado 2003 | 0.398 | Prado 2003 |  | M/PG | Prado 2003 |
| Hyla_tsinlingensis | -2.076 | Liao 2018 | 0.364 | Liao 2018 |  | M/PG | Zeng 2014 |
| Hylarana_guentheri | -1.409 | Zheng 2014 | 1.348 | Zheng 2014 |  | M/PG | Zeng 2014 |
| Lechriodus_fletcheri | -2.046 | Byrne 2002 | 0.891 | Byrne 2002 |  | M/PG | Byrne 2002 |
| Leptodactylus_chaquensis | 0.133 | Prado 2003 | 1.516 | Prado 2003 |  | PA/PGA | Prado 2003 |
| Leptodactylus_fuscus | -2.301 | Prado 2003 | 0.886 | Prado 2003 |  | M/PG | Prado 2003 |
| Leptodactylus_labyrinthicus | -0.658 | Prado 2003 | 2.749 | Prado 2003 |  | M/PG | Prado 2003 |
| Leptodactylus_mystacinus | -2.027 | Prado 2003 | 1.185 | Prado 2003 |  | M/PG | Prado 2003 |
| Leptodactylus_notoaktites | -2.301 | Prado 2003 | 1.029 | Prado 2003 |  | M/PG | Prado 2003 |
| Leptodactylus_ocellatus | -0.886 | Prado 2003 | 2.055 | Prado 2003 |  | M/PG | Prado 2003 |
| Leptodactylus_podicipinus | -1.481 | Prado 2003 | 0.633 | Prado 2003 |  | PA/PGA | Prado 2003 |
| Limnodynastes_convexiusculus | -1.670 | Byrne 2002 | 0.998 | Byrne 2002 |  | M/PG | Byrne 2002 |
| Limnodynastes_dorsalis | -1.583 | Byrne 2002 | 1.278 | Byrne 2002 |  | M/PG | Byrne 2002 |
| Limnodynastes_dumerilii | -1.883 | Byrne 2002 | 1.185 | Byrne 2002 |  | M/PG | Byrne 2002 |
| Limnodynastes_fletcheri | -2.137 | Byrne 2002 | 1.055 | Byrne 2002 |  | M/PG | Byrne 2002 |
| Limnodynastes_interioris | -1.407 | Byrne 2002 | 1.562 | Byrne 2002 |  | M/PG | Byrne 2002 |
| Limnodynastes_ornatus | -2.469 | Byrne 2002 | 0.739 | Byrne 2002 |  | M/PG | Byrne 2002 |
| Limnodynastes_peronii | -2.174 | Byrne 2002 | 1.085 | Byrne 2002 |  | M/PG | Byrne 2002 |
| Limnodynastes_salmini | -1.646 | Byrne 2002 | 1.319 | Byrne 2002 |  | M/PG | Byrne 2002 |
| Limnodynastes_spenceri | -3.222 | Byrne 2002 | 0.617 | Byrne 2002 |  | M/PG | Byrne 2002 |
| Limnodynastes_tasmaniensis | -2.538 | Byrne 2002 | 0.528 | Byrne 2002 |  | M/PG | Byrne 2002 |
| Limnodynastes_terraereginae | -2.114 | Byrne 2002 | 1.344 | Byrne 2002 |  | M/PG | Byrne 2002 |
| Litoria_adelaidensis | -2.469 | Byrne 2002 | 0.488 | Byrne 2002 |  | M/PG | Byrne 2002 |
| Litoria_aurea | -0.990 | Byrne 2002 | 1.097 | Byrne 2002 |  | PA/PGA | Byrne 2002 |
| Litoria_bicolor | -2.921 | Byrne 2002 | -0.203 | Byrne 2002 |  | M/PG | Byrne 2002 |
| Litoria_brevipalmata | -2.284 | Byrne 2002 | 0.532 | Byrne 2002 |  | M/PG | Byrne 2002 |
| Litoria_caerulea | -1.717 | Byrne 2002 | 1.416 | Byrne 2002 |  | M/PG | Byrne 2002 |
| Litoria_cavernicola | -2.229 | Byrne 2002 | 0.728 | Byrne 2002 |  | M/PG | Byrne 2002 |
| Litoria_chloris | -1.660 | Byrne 2002 | 1.101 | Byrne 2002 |  | M/PG | Byrne 2002 |
| Litoria_citropa | -1.921 | Byrne 2002 | 0.935 | Byrne 2002 |  | M/PG | Byrne 2002 |
| Litoria_coplandi | -3.523 | Byrne 2002 | 0.127 | Byrne 2002 |  | M/PG | Byrne 2002 |
| Litoria_cyclorhyncha | -1.139 | Byrne 2002 | 1.244 | Byrne 2002 |  | M/PG | Byrne 2002 |
| Litoria_dahlii | -2.081 | Byrne 2002 | 0.910 | Byrne 2002 |  | M/PG | Byrne 2002 |
| Litoria_dentata | -1.951 | Byrne 2002 | 0.547 | Byrne 2002 |  | M/PG | Byrne 2002 |
| Litoria_electrica | -2.260 | Byrne 2002 | 0.226 | Byrne 2002 |  | M/PG | Byrne 2002 |
| Litoria_eucnemis | -2.244 | Byrne 2002 | 0.575 | Byrne 2002 |  | M/PG | Byrne 2002 |
| Litoria_ewingii | -2.824 | Byrne 2002 | 0.229 | Byrne 2002 |  | M/PG | Byrne 2002 |
| Litoria_fallax | -3.000 | Byrne 2002 | -0.028 | Byrne 2002 |  | M/PG | Byrne 2002 |
| Litoria_freycineti | -2.276 | Byrne 2002 | 0.508 | Byrne 2002 |  | M/PG | Byrne 2002 |
| Litoria_genimaculata | -2.046 | Byrne 2002 | 0.602 | Byrne 2002 |  | M/PG | Byrne 2002 |
| Litoria_gracilenta | -2.620 | Byrne 2002 | 0.404 | Byrne 2002 |  | M/PG | Byrne 2002 |
| Litoria_inermis | -2.678 | Byrne 2002 | 0.276 | Byrne 2002 |  | M/PG | Byrne 2002 |
| Litoria_infrafrenata | -1.391 | Byrne 2002 | 1.388 | Byrne 2002 |  | M/PG | Byrne 2002 |
| Litoria_latopalmata | -2.678 | Byrne 2002 | 0.291 | Byrne 2002 |  | M/PG | Byrne 2002 |
| Litoria_lesueurii | -1.701 | Byrne 2002 | 0.732 | Byrne 2002 |  | M/PG | Byrne 2002 |
| Litoria_littlejohni | -1.914 | Byrne 2002 | 0.824 | Byrne 2002 |  | M/PG | Byrne 2002 |
| Litoria_meiriana | -4.000 | Byrne 2002 | -0.276 | Byrne 2002 |  | M/PG | Byrne 2002 |
| Litoria_moorei | -1.005 | Byrne 2002 | 1.082 | Byrne 2002 |  | PA/PGA | Byrne 2002 |
| Litoria_nannotis | -1.506 | Byrne 2002 | 0.968 | Byrne 2002 |  | M/PG | Byrne 2002 |
| Litoria_nasuta | -2.357 | Byrne 2002 | 0.625 | Byrne 2002 |  | M/PG | Byrne 2002 |
| Litoria_nigrofrenata | -2.194 | Byrne 2002 | 0.367 | Byrne 2002 |  | M/PG | Byrne 2002 |
| Litoria_nyakalensis | -2.745 | Byrne 2002 | 0.400 | Byrne 2002 |  | M/PG | Byrne 2002 |
| Litoria_olongburensis | -2.143 | Byrne 2002 | -0.241 | Byrne 2002 |  |  |  |
| Litoria_pallida | -2.854 | Byrne 2002 | 0.235 | Byrne 2002 |  | M/PG | Byrne 2002 |
| Litoria_paraewingi | -3.000 | Byrne 2002 | 0.081 | Byrne 2002 |  | M/PG | Byrne 2002 |
| Litoria_pearsoniana | -2.959 | Byrne 2002 | 0.037 | Byrne 2002 |  | M/PG | Byrne 2002 |
| Litoria_peronii | -1.963 | Byrne 2002 | 0.769 | Byrne 2002 |  | M/PG | Byrne 2002 |
| Litoria_phyllochroa | -2.824 | Byrne 2002 | 0.065 | Byrne 2002 |  | PA/PGA | Byrne 2002 |
| Litoria_raniformis | -1.262 | Byrne 2002 | 1.185 | Byrne 2002 |  | PA/PGA | Byrne 2002 |
| Litoria_revelata | -3.000 | Byrne 2002 | -0.031 | Byrne 2002 |  |  |  |
| Litoria_rheocola | -2.469 | Byrne 2002 | 0.218 | Byrne 2002 |  | M/PG | Byrne 2002 |
| Litoria_rothii | -2.167 | Byrne 2002 | 0.722 | Byrne 2002 |  | M/PG | Byrne 2002 |
| Litoria_rubella | -2.143 | Byrne 2002 | 0.489 | Byrne 2002 |  | M/PG | Byrne 2002 |
| Litoria_splendida | -1.072 | Byrne 2002 | 1.568 | Byrne 2002 |  | M/PG | Byrne 2002 |
| Litoria_subglandulosa | -1.967 | Byrne 2002 | 0.754 | Byrne 2002 |  | M/PG | Byrne 2002 |
| Litoria_tornieri | -3.097 | Byrne 2002 | 0.137 | Byrne 2002 |  | M/PG | Byrne 2002 |
| Litoria_tyleri | -1.764 | Byrne 2002 | 0.754 | Byrne 2002 |  | M/PG | Byrne 2002 |
| Litoria_verreauxii | -2.921 | Byrne 2002 | 0.240 | Byrne 2002 |  | M/PG | Byrne 2002 |
| Litoria_watjulumensis | -2.357 | Byrne 2002 | 0.413 | Byrne 2002 |  | M/PG | Byrne 2002 |
| Litoria_xanthomera | -2.041 | Byrne 2002 | 0.768 | Byrne 2002 |  | M/PG | Byrne 2002 |
| Mixophyes_balbus | -1.648 | Byrne 2002 | 1.101 | Byrne 2002 |  | M/PG | Byrne 2002 |
| Mixophyes_fasciolatus | -1.481 | Byrne 2002 | 1.307 | Byrne 2002 |  | M/PG | Byrne 2002 |
| Mixophyes_schevilli | -1.439 | Byrne 2002 | 1.503 | Byrne 2002 |  | M/PG | Byrne 2002 |
| Myobatrachus_gouldii | -2.420 | Byrne 2002 | 0.563 | Byrne 2002 |  | M/PG | Byrne 2002 |
| Nanorana_parkeri | -1.428 | Liao 2018 | 1.244 | Liao 2018 |  | M/PG | Zeng 2014 |
| Nanorana_pleskei | -1.428 | Zheng 2014 | 1.244 | Zheng 2014 |  | M/PG | Zeng 2014 |
| Nanorana_quadranus | 0.192 | Zheng 2014 | 1.637 | Zheng 2014 |  | PA/PGA | Zeng 2014 |
| Nanorana_ventripunctata | -1.740 | Zheng 2014 | 0.629 | Zheng 2014 |  | M/PG | Zeng 2014 |
| Nanorana_yunnanensis | -0.388 | Zheng 2014 | 1.477 | Zheng 2014 |  | M/PG | Zeng 2014 |
| Neobatrachus_pelobatoides | -1.160 | Byrne 2002 | 0.785 | Byrne 2002 |  | PA/PGA | Byrne 2002 |
| Neobatrachus_pictus | -0.818 | Byrne 2002 | 1.091 | Byrne 2002 |  | M/PG | Byrne 2002 |
| Neobatrachus_sudelli | -1.236 | Byrne 2002 | 0.885 | Byrne 2002 |  | M/PG | Byrne 2002 |
| Notaden_bennettii | -2.009 | Byrne 2002 | 1.238 | Byrne 2002 |  | M/PG | Byrne 2002 |
| Notaden_melanoscaphus | -1.762 | Byrne 2002 | 0.950 | Byrne 2002 |  | M/PG | Byrne 2002 |
| Nyctimystes_dayi | -2.770 | Byrne 2002 | 0.368 | Byrne 2002 |  | M/PG | Byrne 2002 |
| Nyctimystes_papua | -2.770 | Hayward 2011 | 0.368 | Hayward 2011 |  |  |  |
| Odorrana_grahami | -1.320 | Zheng 2014 | 1.360 | Zheng 2014 |  | M/PG | Zeng 2014 |
| Odorrana_margaretae | -1.188 | Zheng 2014 | 1.521 | Zheng 2014 |  | M/PG | Zeng 2014 |
| Paracrinia_haswelli | -2.824 | Byrne 2002 | 0.290 | Byrne 2002 |  | M/PG | Byrne 2002 |
| Pelophylax_hubeiensis | -2.495 | Liao 2018 | 0.708 | Liao 2018 |  | M/PG | Zeng 2014 |
| Pelophylax_nigromaculatus | -1.618 | Zheng 2014 | 1.343 | Zheng 2014 |  | M/PG | Zeng 2014 |
| Pelophylax_porosus | -1.606 | Prado 2003 | 1.173 | Prado 2003 |  | M/PG | Prado 2003 |
| Philoria_sphagnicolus | -1.959 | Byrne 2002 | 0.326 | Byrne 2002 |  | M/PG | Byrne 2002 |
| Phyllomedusa_hypochondrialis | -1.921 | Prado 2003 | 0.431 | Prado 2003 |  | M/PG | Prado 2003 |
| Physalaemus_albonotatus | -2.854 | Prado 2003 | 0.079 | Prado 2003 |  | M/PG | Prado 2003 |
| Polypedates_leucomystax | -1.304 | Prado 2003 | 0.857 | Prado 2003 |  | PA/PGA | Prado 2003 |
| Polypedates_megacephalus | -0.995 | Zheng 2014 | 0.916 | Zheng 2014 |  | PA/PGA | Zeng 2014 |
| Polypedates_mutus | -1.383 | Liao 2018 | 0.692 | Liao 2018 |  | PA/PGA | Zeng 2014 |
| Pseudophryne_bibroni | -1.947 | Byrne 2002 | 0.077 | Byrne 2002 |  | M/PG | Byrne 2002 |
| Pseudophryne_coriacea | -2.824 | Byrne 2002 | 0.054 | Byrne 2002 |  | M/PG | Byrne 2002 |
| Quasipaa_boulengeri | -0.348 | Zheng 2014 | 1.722 | Zheng 2014 |  | M/PG | Zeng 2014 |
| Rana_catesbeiana | -1.000 | Hayward 2011 | 2.494 | Hayward 2011 |  | M/PG | Ryan 1980 |
| Rana_chaochiaoensis | -1.860 | Zheng 2014 | 0.913 | Zheng 2014 |  | M/PG | Zeng 2014 |
| Rana_chensinensis | -1.607 | Zheng 2014 | 0.997 | Zheng 2014 |  | M/PG | Zeng 2014 |
| Rana_japonica | -1.836 | Liao 2018 | 1.476 | Liao 2018 |  | M/PG | Zeng 2014 |
| Rana_kukunoris | -1.096 | Liao 2011 | 0.994 | Liao 2011 |  | M/PG | Liao 2011 |
| Rana_omeimontis | -2.796 | Zheng 2014 | 1.074 | Zheng 2014 |  | M/PG | Zeng 2014 |
| Rana_ornativentris | -1.444 | Prado 2003 | 1.140 | Prado 2003 |  | M/PG | Prado 2003 |
| Rana_tagoi | -1.635 | Prado 2003 | 0.771 | Prado 2003 |  | M/PG | Prado 2003 |
| Rana_tsushimensis | -2.202 | Kusano 1991 | 0.509 | Kusano 1991 |  | M/PG | Kusano 1991 |
| Rhacophorus_arboreus | -0.123 | Prado 2003 | 1.143 | Prado 2003 |  | PA/PGA | Prado 2003 |
| Rhacophorus_chenfui | -0.882 | Zheng 2014 | 0.840 | Zheng 2014 |  | PA/PGA | Zeng 2014 |
| Rhacophorus_dennysi | -1.014 | Liao 2018 | 0.728 | Liao 2018 |  | PA/PGA | Zeng 2014 |
| Rhacophorus_dugritei | -1.024 | Zheng 2014 | 0.849 | Zheng 2014 |  | PA/PGA | Zeng 2014 |
| Rhacophorus_omeimontis | -0.350 | Zheng 2014 | 1.199 | Zheng 2014 |  | PA/PGA | Zeng 2014 |
| Rhacophorus_schlegelii | -1.475 | Prado 2003 | 0.505 | Prado 2003 |  | PA/PGA | Prado 2003 |
| Rheobatrachus_silus | -2.027 | Byrne 2002 | 0.777 | Byrne 2002 |  |  |  |
| Rhinella_marina | -0.463 | Kusano 1991 | 2.063 | Kusano 1991 |  | M/PG | Kusano 1991 |
| Scinax_acuminatus | -2.076 | Prado 2003 | 0.763 | Prado 2003 |  | M/PG | Prado 2003 |
| Scinax_nasicus | -2.222 | Prado 2003 | 0.255 | Prado 2003 |  | M/PG | Prado 2003 |
| Taudactylus_acutirostris | -3.523 | Byrne 2002 | -0.059 | Byrne 2002 |  | M/PG | Byrne 2002 |
| Tomopterna_cryptotis | -1.745 | Jennions 1993 | 0.816 | Jennions 1993 |  |  |  |
| Uperoleia_laevigata | -2.770 | Byrne 2002 | 0.118 | Byrne 2002 |  | M/PG | Byrne 2002 |
| Uperoleia_littlejohni | -2.456 | Byrne 2002 | 0.041 | Byrne 2002 |  | M/PG | Byrne 2002 |
| Fejervarya_limnocharis | -1.686 | Zheng 2014 | 0.659 | Zheng 2014 |  | M/PG | Zeng 2014 |
| Ptychadena_anchietae | -1.818 | Prado 2003 | 0.748 | Prado 2003 |  | M/PG | Prado 2003 |
| Ptychadena_oxyrhynchus | -1.504 | Jennions 1993 | 1.172 | Jennions 1993 |  |  |  |
| Ptychadena_porosissima | -2.022 | Jennions 1993 | 0.740 | Jennions 1993 |  |  |  |
| Ptychadena_mascareniensis | -1.547 | Jennions 1993 | 1.017 | Jennions 1993 |  |  |  |
| Phrynobatrachus_natalensis | -2.553 | Jennions 1993 | 0.412 | Jennions 1993 |  |  |  |
| Microhyla_ornata | -2.959 | Zheng 2014 | -0.143 | Zheng 2014 |  | M/PG | Zeng 2014 |
| Hyperolius_tuberilinguis | -2.292 | Jennions 1993 | 0.149 | Jennions 1993 |  |  |  |
| Hyperolius_marmoratus | -2.538 | Jennions 1993 | 0.130 | Jennions 1993 |  | M/PG | Telford 1988 |
| Hyperolius_argus | -2.387 | Jennions 1993 | 0.301 | Jennions 1993 |  |  |  |
| Afrixalus_fornasini | -2.420 | Jennions 1993 | 0.408 | Jennions 1993 |  |  |  |
| Kassina_senegalensis | -1.493 | Jennions 1993 | 0.699 | Jennions 1993 |  |  |  |
| Kassina_maculata | -1.151 | Jennions 1993 | 1.192 | Jennions 1993 |  |  |  |
| Leptopelis_natalensis | -2.041 | Prado 2003 | 0.672 | Prado 2003 |  | M/PG | Prado 2003 |
| Hemisus_marmoratus | -2.409 | Jennions 1993 | 0.500 | Jennions 1993 |  | M/PG | Kaminsky 1999 |
| Xenophrys_omeimontis | -1.273 | Liao 2018 | 0.652 | Liao 2018 |  | M/PG | Zeng 2014 |
| Scaphiopus_couchii | -1.347 | Hayward 2011 | 1.476 | Hayward 2011 |  | M/PG | Pfennig 2000; Klein 2000 |
| Bombina_maxima | -0.779 | Zheng 2014 | 1.278 | Zheng 2014 |  | M/PG | Zeng 2014 |
| **Birds** | | | | | | | |
| Ailuroedus_melanotis | -0.161 | Calhim 2006 | 2.173 | Calhim 2006 | Original data is for Ailuroedus_buccoides - taxa included by genus matching. All members of this genus are defined as monogamous in source. | M | Lenz 1994 |
| Anhinga_anhinga | 0.748 | Calhim 2006 | 3.245 | Calhim 2006 | Original data is for Anhinga_melanogaster - taxa included by genus matching. |  |  |
| Calandrella_acutirostris | 0.013 | Calhim 2006 | 1.322 | Calhim 2006 | Original data is for Calandrella_cinerea - taxa included by genus matching. |  |  |
| Cinclus_cinclus | -1.097 | Calhim 2006 | 1.653 | Calhim 2006 | Original data is for Cinclus_leucocephalus - taxa included by genus matching. | M | Biagolini 2017 |
| Cotinga_cayana | -0.456 | Calhim 2006 | 1.819 | Calhim 2006 | Original data is for Cotinga_amabilis - taxa included by genus matching. |  |  |
| Cyphorhinus_arada | -1.222 | Hayward 2011 | 1.391 | Hayward 2011 | Original data is for Cyphorhinus_phaeocephalus - taxa included by genus matching. |  |  |
| Ducula_aenea | 0.344 | Calhim 2006 | 2.669 | Calhim 2006 | Original data is for Ducula_spilorrhoa - taxa included by genus matching. |  |  |
| Grallaria_andicolus | 0.004 | Calhim 2006 | 1.822 | Calhim 2006 | Original data is for Grallaria_quitensis - taxa included by genus matching. |  |  |
| Hylopezus_berlepschi | -1.398 | Calhim 2006 | 1.623 | Calhim 2006 | Original data is for Hylopezus_perspicillatus - taxa included by genus matching. |  |  |
| Laniocera_hypopyrra | -1.222 | Calhim 2006 | 1.690 | Calhim 2006 | Original data is for Laniocera_rufescens - taxa included by genus matching. |  |  |
| Oncostoma_cinereigulare | -1.699 | Calhim 2006 | 0.903 | Calhim 2006 | Original data is for Oncostoma_olivaceum - taxa included by genus matching. |  |  |
| Sphecotheres_vieilloti | 0.104 | Calhim 2006 | 2.140 | Calhim 2006 | Original data is for Sphecotheres_viridis - taxa included by genus matching. |  |  |
| Acanthagenys_rufogularis | -0.347 | Hayward 2011 | 1.699 | Hayward 2011 |  | M | Garamszegi 2005; Pitcher 2005 |
| Acanthiza_apicalis | -1.097 | Calhim 2006 | 0.845 | Calhim 2006 |  | M | Pitcher 2005 |
| Acanthiza_chrysorrhoa | -1.097 | Calhim 2006 | 0.903 | Calhim 2006 |  | C | Pitcher 2005 |
| Acanthiza_lineata | -0.854 | Calhim 2006 | 0.857 | Calhim 2006 |  | C | Pitcher 2005 |
| Acanthiza_nana | -0.367 | Calhim 2006 | 0.845 | Calhim 2006 |  | C | Pitcher 2005 |
| Acanthiza_pusilla | -0.959 | Calhim 2006 | 0.881 | Calhim 2006 |  | M | Pitcher 2005 |
| Acanthiza_reguloides | -1.000 | Calhim 2006 | 0.881 | Calhim 2006 |  | M | Pitcher 2005 |
| Acanthiza_uropygialis | -1.523 | Calhim 2006 | 0.857 | Calhim 2006 |  | C | Pitcher 2005 |
| Acanthorhynchus_tenuirostris | -0.921 | Calhim 2006 | 1.086 | Calhim 2006 |  | M | Pitcher 2005 |
| Accipiter_cirrocephalus | -0.921 | Calhim 2006 | 2.111 | Calhim 2006 |  | M | Pitcher 2005 |
| Accipiter_gentilis | 0.863 | Calhim 2006 | 2.935 | Calhim 2006 |  | M | Pitcher 2005 |
| Accipiter_nisus | -0.276 | Calhim 2006 | 2.146 | Calhim 2006 |  | M | Pitcher 2005 |
| Accipiter_novaehollandiae | -0.699 | Calhim 2006 | 2.549 | Calhim 2006 |  | M | Pitcher 2005 |
| Sturnus_tristis | -0.456 | Calhim 2006 | 2.137 | Calhim 2006 |  | M | Pitcher 2005 |
| Acrocephalus_arundinaceus | -0.337 | Calhim 2006 | 1.435 | Calhim 2006 |  | PG | Pitcher 2005; Biagolini 2017 |
| Acrocephalus_melanopogon | -0.569 | Calhim 2006 | 1.041 | Calhim 2006 |  | M | Biagolini 2017 |
| Acrocephalus_paludicola | -0.432 | Calhim 2006 | 1.064 | Calhim 2006 |  | PG | Pitcher 2005 |
| Acrocephalus_palustris | -0.854 | Calhim 2006 | 1.079 | Calhim 2006 |  | PG | Pitcher 2005; Biagolini 2017 |
| Acrocephalus_schoenobaenus | -0.770 | Calhim 2006 | 1.083 | Calhim 2006 |  | PG | Pitcher 2005; Biagolini 2017 |
| Acrocephalus_scirpaceus | -0.824 | Hayward 2011 | 1.079 | Hayward 2011 |  | PG | Garamszegi 2005; Pitcher 2005; Biagolini 2017 |
| Acrocephalus_stentoreus | -1.523 | Calhim 2006 | 1.204 | Calhim 2006 |  | PG | Pitcher 2005 |
| Actitis_macularia | -0.252 | Hayward 2011 | 1.605 | Hayward 2011 |  | PA/PGA | Garamszegi 2005; Griffith 2002; Pitcher 2005; Biagolini 2017; Szekely 2000 |
| Aegithalos_caudatus | -1.523 | Calhim 2006 | 0.914 | Calhim 2006 |  | C | Biagolini 2017 |
| Aegolius_funereus | 0.107 | Calhim 2006 | 2.000 | Calhim 2006 |  | M | Pitcher 2005; Biagolini 2017 |
| Aethia_pusilla | 0.407 | Calhim 2006 | 1.991 | Calhim 2006 |  | M | Pitcher 2005 |
| Agelaius_phoeniceus | -0.268 | Hayward 2011 | 1.803 | Hayward 2011 |  | PG | Pitcher 2005; Biagolini 2017 |
| Agelaius_tricolor | 0.053 | Calhim 2006 | 1.826 | Calhim 2006 |  | PG | Pitcher 2005 |
| Ailuroedus_crassirostris | -0.444 | Calhim 2006 | 2.179 | Calhim 2006 | All members of this genus are defined as monogamous in source. | M | Pitcher 2005; Lenz 1994 |
| Aimophila_aestivalis | -0.921 | Calhim 2006 | 1.260 | Calhim 2006 |  |  |  |
| Peucaea_botterii | -0.509 | Calhim 2006 | 1.294 | Calhim 2006 |  | M | Pitcher 2005 |
| Aimophila_cassinii | -0.678 | Calhim 2006 | 1.255 | Calhim 2006 |  | M | Pitcher 2005 |
| Aimophila_ruficeps | -0.509 | Calhim 2006 | 1.292 | Calhim 2006 |  | M | Pitcher 2005 |
| Aix_sponsa | -0.009 | Calhim 2006 | 2.833 | Calhim 2006 |  | M | Garamszegi 2005; Pitcher 2005 |
| Alauda_arvensis | -0.357 | Calhim 2006 | 1.591 | Calhim 2006 |  | M | Pitcher 2005; Biagolini 2017 |
| Alca_torda | 0.568 | Calhim 2006 | 2.857 | Calhim 2006 |  | M | Pitcher 2005 |
| Aleadryas_rufinucha | -0.387 | Calhim 2006 | 1.568 | Calhim 2006 |  | M | Pitcher 2005 |
| Alectoris_chukar | 0.450 | Calhim 2006 | 2.712 | Calhim 2006 |  |  |  |
| Alectoris_graeca | 0.447 | Calhim 2006 | 2.653 | Calhim 2006 |  | M | Pitcher 2005 |
| Alectoris_rufa | 0.860 | Calhim 2006 | 2.653 | Calhim 2006 |  | M | Pitcher 2005 |
| Alectura_lathami | 0.243 | Calhim 2006 | 3.389 | Calhim 2006 |  | PA/PGA | Pitcher 2005; Biagolini 2017 |
| Alisterus_scapularis | -0.959 | Calhim 2006 | 2.324 | Calhim 2006 |  | M | Pitcher 2005 |
| Alle_alle | 0.405 | Calhim 2006 | 2.146 | Calhim 2006 |  | M | Pitcher 2005; Biagolini 2017 |
| Amalocichla_incerta | -0.678 | Calhim 2006 | 1.423 | Calhim 2006 |  | M | Pitcher 2005 |
| Amandava_amandava | -1.097 | Calhim 2006 | 0.903 | Calhim 2006 |  | PG | Pitcher 2005 |
| Amblyornis_macgregoriae | -0.268 | Calhim 2006 | 2.104 | Calhim 2006 | Lekking | PG | Pitcher 2005; Diamond 1986; Pruett-Jones 1982; Lenz 1994 |
| Ammodramus_bairdii | -0.481 | Calhim 2006 | 1.276 | Calhim 2006 |  | M | Pitcher 2005 |
| Ammodramus_caudacutus | 0.100 | Calhim 2006 | 1.241 | Calhim 2006 |  | PG | Pitcher 2005 |
| Ammodramus_leconteii | -0.301 | Calhim 2006 | 1.130 | Calhim 2006 |  | M | Pitcher 2005 |
| Ammodramus_maritimus | -0.481 | Calhim 2006 | 1.352 | Calhim 2006 |  | M | Pitcher 2005; Biagolini 2017 |
| Ammodramus_nelsoni | -0.886 | Calhim 2006 | 1.220 | Calhim 2006 |  |  |  |
| Ammodramus_savannarum | -0.409 | Calhim 2006 | 1.248 | Calhim 2006 |  | M | Pitcher 2005 |
| Ammomanes_grayi | -0.796 | Calhim 2006 | 1.301 | Calhim 2006 |  | M | Pitcher 2005 |
| Amphispiza_bilineata | -0.602 | Hayward 2011 | 1.130 | Hayward 2011 |  | M | Garamszegi 2005; Pitcher 2005 |
| Amytornis_purnelli | -1.222 | Calhim 2006 | 1.342 | Calhim 2006 |  | C | Pitcher 2005 |
| Amytornis_striatus | -0.398 | Calhim 2006 | 1.279 | Calhim 2006 |  | M | Pitcher 2005 |
| Anas_acuta | 0.977 | Calhim 2006 | 2.954 | Calhim 2006 |  | M | Pitcher 2005 |
| Anas_americana | 0.949 | Calhim 2006 | 2.899 | Calhim 2006 |  | M | Pitcher 2005 |
| Anas_castanea | 0.356 | Calhim 2006 | 2.819 | Calhim 2006 |  | M | Pitcher 2005 |
| Anas_clypeata | 0.360 | Calhim 2006 | 2.785 | Calhim 2006 |  | M | Pitcher 2005 |
| Anas_crecca | 0.593 | Calhim 2006 | 2.556 | Calhim 2006 |  | M | Pitcher 2005 |
| Anas_discors | 0.225 | Calhim 2006 | 2.591 | Calhim 2006 |  | M | Pitcher 2005 |
| Anas_georgica | 0.991 | Calhim 2006 | 2.800 | Calhim 2006 |  | M | Pitcher 2005 |
| Anas_gracilis | -0.155 | Calhim 2006 | 2.690 | Calhim 2006 |  | M | Pitcher 2005 |
| Anas_platyrhynchos | 0.982 | Hayward 2011 | 3.057 | Hayward 2011 |  | M | Garamszegi 2005; Pitcher 2005; Biagolini 2017 |
| Anas_rhynchotis | -0.347 | Calhim 2006 | 2.824 | Calhim 2006 |  | M | Pitcher 2005 |
| Anas_rubripes | -0.244 | Calhim 2006 | 3.146 | Calhim 2006 |  | M | Pitcher 2005 |
| Anas_superciliosa | 0.778 | Calhim 2006 | 3.000 | Calhim 2006 |  | M | Pitcher 2005 |
| Anisognathus_igniventris | -0.432 | Calhim 2006 | 1.531 | Calhim 2006 |  | M | Pitcher 2005 |
| Anous_stolidus | -0.409 | Calhim 2006 | 2.083 | Calhim 2006 |  | M | Pitcher 2005 |
| Anser_albifrons | -0.092 | Calhim 2006 | 3.432 | Calhim 2006 |  | M | Pitcher 2005 |
| Anser_anser | 0.602 | Calhim 2006 | 3.539 | Calhim 2006 |  | M | Pitcher 2005 |
| Anthochaera_carunculata | -0.284 | Calhim 2006 | 2.053 | Calhim 2006 |  | M | Pitcher 2005 |
| Anthochaera_chrysoptera | -0.398 | Calhim 2006 | 1.924 | Calhim 2006 |  | M | Pitcher 2005 |
| Anthochaera_paradoxa | -1.301 | Calhim 2006 | 2.253 | Calhim 2006 |  | M | Pitcher 2005 |
| Anthus_campestris | -0.367 | Calhim 2006 | 1.362 | Calhim 2006 |  |  |  |
| Anthus_pratensis | -0.495 | Calhim 2006 | 1.255 | Calhim 2006 |  | M | Pitcher 2005 |
| Anthus_rubescens | -0.180 | Calhim 2006 | 1.338 | Calhim 2006 |  | M | Pitcher 2005 |
| Anthus_spinoletta | -0.372 | Calhim 2006 | 1.377 | Calhim 2006 |  | PA/PGA | Pitcher 2005; Biagolini 2017 |
| Aphelocoma_coerulescens | 0.258 | Hayward 2011 | 1.904 | Hayward 2011 |  | C | Griffith 2002; Pitcher 2005; Biagolini 2017 |
| Aphelocoma_ultramarina | 0.170 | Calhim 2006 | 2.078 | Calhim 2006 |  | C | Griffith 2002; Pitcher 2005; Biagolini 2017 |
| Aplonis_metallica | -0.244 | Calhim 2006 | 1.806 | Calhim 2006 |  | M | Pitcher 2005 |
| Aptenodytes_forsteri | 1.602 | Hayward 2011 | 4.531 | Hayward 2011 |  | M | Garamszegi 2005; Pitcher 2005 |
| Apus_apus | 0.053 | Calhim 2006 | 1.613 | Calhim 2006 |  | M | Pitcher 2005; Biagolini 2017 |
| Aquila_chrysaetos | 0.867 | Calhim 2006 | 3.569 | Calhim 2006 |  | M | Pitcher 2005 |
| Hieraaetus_fasciatus | 0.362 | Calhim 2006 | 3.223 | Calhim 2006 |  | M | Pitcher 2005 |
| Aramus_guarauna | 0.702 | Calhim 2006 | 3.142 | Calhim 2006 |  | M | Pitcher 2005 |
| Archilochus_colubris | -1.699 | Calhim 2006 | 0.459 | Calhim 2006 |  | PG | Pitcher 2005 |
| Ardea_herodias | 0.176 | Calhim 2006 | 3.411 | Calhim 2006 |  | M | Pitcher 2005 |
| Arenaria_interpres | -0.699 | Calhim 2006 | 2.041 | Calhim 2006 |  | M | Pitcher 2005; Szekely 2000 |
| Buarremon_brunneinucha | -0.721 | Calhim 2006 | 1.668 | Calhim 2006 |  | M | Pitcher 2005 |
| Arremonops_rufivirgatus | -0.796 | Calhim 2006 | 1.373 | Calhim 2006 |  | M | Pitcher 2005 |
| Arses_telescopthalmus | -0.602 | Calhim 2006 | 1.204 | Calhim 2006 |  | M | Pitcher 2005 |
| Artamus_cinereus | -0.268 | Calhim 2006 | 1.556 | Calhim 2006 |  | C | Pitcher 2005 |
| Artamus_cyanopterus | -0.347 | Calhim 2006 | 1.505 | Calhim 2006 |  | C | Pitcher 2005 |
| Artamus_leucorhynchus | -1.155 | Calhim 2006 | 1.591 | Calhim 2006 |  | C | Pitcher 2005 |
| Artamus_minor | -0.770 | Calhim 2006 | 1.255 | Calhim 2006 |  | C | Pitcher 2005 |
| Asio_flammeus | 0.583 | Calhim 2006 | 2.550 | Calhim 2006 |  | M | Pitcher 2005 |
| Asio_otus | 0.433 | Calhim 2006 | 2.436 | Calhim 2006 |  | M | Pitcher 2005 |
| Astrapia_splendidissima | -0.495 | Calhim 2006 | 2.079 | Calhim 2006 | Lekking | PG | Pitcher 2005; Hoglund 1989; Irestedt et al 2009 |
| Astrapia_stephaniae | -0.092 | Calhim 2006 | 2.358 | Calhim 2006 | Lekking | PG | Pitcher 2005; Hoglund 1989; Irestedt et al 2009 |
| Atlapetes_albinucha | -0.620 | Calhim 2006 | 1.510 | Calhim 2006 |  | M | Pitcher 2005 |
| Atlapetes_pileatus | -0.721 | Calhim 2006 | 1.380 | Calhim 2006 |  | M | Pitcher 2005 |
| Attagis_gayi | -0.854 | Calhim 2006 | 2.493 | Calhim 2006 |  | M | Pitcher 2005; Szekely 2000 |
| Aulacorhynchus_prasinus | -0.620 | Calhim 2006 | 2.255 | Calhim 2006 |  | M | Pitcher 2005 |
| Auriparus_flaviceps | -1.409 | Calhim 2006 | 0.814 | Calhim 2006 |  | M | Pitcher 2005 |
| Aviceda_subcristata | -0.347 | Calhim 2006 | 2.529 | Calhim 2006 |  | M | Pitcher 2005 |
| Aythya_affinis | 0.528 | Calhim 2006 | 2.806 | Calhim 2006 |  | M | Pitcher 2005 |
| Aythya_collaris | -0.959 | Calhim 2006 | 2.877 | Calhim 2006 |  | M | Pitcher 2005 |
| Aythya_marila | -0.959 | Calhim 2006 | 2.969 | Calhim 2006 |  | M | Pitcher 2005 |
| Aythya_valisineria | -0.721 | Calhim 2006 | 3.096 | Calhim 2006 |  | M | Pitcher 2005 |
| Baeolophus_bicolor | -0.770 | Calhim 2006 | 1.318 | Calhim 2006 |  | M | Pitcher 2005 |
| Baeolophus_inornatus | -1.301 | Calhim 2006 | 1.272 | Calhim 2006 |  | M | Pitcher 2005 |
| Baeolophus_wollweberi | -1.222 | Calhim 2006 | 0.996 | Calhim 2006 |  | C | Pitcher 2005 |
| Baryphthengus_martii | -1.000 | Calhim 2006 | 2.202 | Calhim 2006 |  | M | Pitcher 2005 |
| Basileuterus_belli | -0.745 | Calhim 2006 | 1.021 | Calhim 2006 |  | M | Pitcher 2005 |
| Basileuterus_bivittatus | -0.699 | Calhim 2006 | 1.149 | Calhim 2006 |  | M | Pitcher 2005 |
| Basileuterus_coronatus | -0.678 | Calhim 2006 | 1.199 | Calhim 2006 |  | M | Pitcher 2005 |
| Basileuterus_melanogenys | -0.824 | Calhim 2006 | 1.130 | Calhim 2006 |  | M | Pitcher 2005 |
| Basileuterus_rufifrons | -0.921 | Calhim 2006 | 1.000 | Calhim 2006 |  | M | Pitcher 2005 |
| Biziura_lobata | 0.513 | Calhim 2006 | 3.380 | Calhim 2006 |  |  |  |
| Bombycilla_cedrorum | -1.000 | Calhim 2006 | 1.500 | Calhim 2006 |  | M | Pitcher 2005 |
| Bombycilla_garrulus | -0.444 | Calhim 2006 | 1.695 | Calhim 2006 |  | M | Pitcher 2005 |
| Tetrastes_bonasia | 0.053 | Calhim 2006 | 2.559 | Calhim 2006 |  |  |  |
| Bonasa_umbellus | 0.286 | Calhim 2006 | 2.761 | Calhim 2006 | Lekking |  | Pitcher 2005 |
| Branta_bernicla | -0.201 | Hayward 2011 | 3.161 | Hayward 2011 |  | M | Pitcher 2005; Biagolini 2017 |
| Branta_canadensis | 0.629 | Calhim 2006 | 3.589 | Calhim 2006 |  | M | Pitcher 2005; Biagolini 2017 |
| Branta_leucopsis | 0.188 | Calhim 2006 | 3.252 | Calhim 2006 |  | M | Pitcher 2005; Biagolini 2017 |
| Branta_sandvicensis | 0.137 | Calhim 2006 | 3.333 | Calhim 2006 |  | M | Pitcher 2005 |
| Brotogeris_cyanoptera | -0.387 | Calhim 2006 | 1.866 | Calhim 2006 |  | M | Pitcher 2005 |
| Bubo_bubo | 0.825 | Calhim 2006 | 3.391 | Calhim 2006 |  | M | Pitcher 2005 |
| Bubulcus_ibis | -0.569 | Calhim 2006 | 2.562 | Calhim 2006 |  | M | Pitcher 2005 |
| Bucephala_albeola | -0.523 | Calhim 2006 | 2.675 | Calhim 2006 |  | M | Pitcher 2005 |
| Burhinus_grallarius | -0.268 | Calhim 2006 | 2.827 | Calhim 2006 |  | M | Pitcher 2005; Szekely 2000 |
| Buteo_buteo | 0.403 | Calhim 2006 | 2.863 | Calhim 2006 |  | M | Pitcher 2005 |
| Buteo_jamaicensis | 0.124 | Calhim 2006 | 2.978 | Calhim 2006 |  | M | Pitcher 2005 |
| Buteo_lagopus | -0.569 | Calhim 2006 | 2.928 | Calhim 2006 |  | M | Pitcher 2005 |
| Buteo_magnirostris | -0.745 | Calhim 2006 | 2.430 | Calhim 2006 |  | M | Pitcher 2005 |
| Buteo_nitidus | -0.699 | Calhim 2006 | 2.668 | Calhim 2006 |  | M | Pitcher 2005 |
| Buteo_platypterus | -0.222 | Calhim 2006 | 2.623 | Calhim 2006 |  | M | Pitcher 2005 |
| Buthraupis_montana | -0.222 | Calhim 2006 | 2.009 | Calhim 2006 |  | M | Pitcher 2005 |
| Cacatua_galerita | -0.469 | Calhim 2006 | 2.921 | Calhim 2006 |  | M | Pitcher 2005 |
| Cacicus_cela | -0.149 | Calhim 2006 | 2.020 | Calhim 2006 |  | PG | Pitcher 2005 |
| Cairina_moschata | 1.477 | Calhim 2006 | 3.556 | Calhim 2006 |  | PG | Pitcher 2005 |
| Calamanthus_campestris | -0.921 | Calhim 2006 | 1.041 | Calhim 2006 |  | M | Pitcher 2005 |
| Calamospiza_melanocorys | -0.060 | Calhim 2006 | 1.602 | Calhim 2006 |  | PG | Pitcher 2005 |
| Calcarius_lapponicus | -0.268 | Hayward 2011 | 1.436 | Hayward 2011 |  | PG | Pitcher 2005 |
| Calcarius_mccownii | -0.638 | Calhim 2006 | 1.403 | Calhim 2006 |  | M | Pitcher 2005 |
| Calcarius_ornatus | -0.569 | Calhim 2006 | 1.297 | Calhim 2006 |  | M | Pitcher 2005 |
| Calcarius_pictus | 0.079 | Calhim 2006 | 1.455 | Calhim 2006 |  | PA/PGA | Griffith 2002; Pitcher 2005; Biagolini 2017 |
| Calidris_alba | -0.354 | Calhim 2006 | 1.700 | Calhim 2006 |  | PA/PGA | Pitcher 2005; Szekely 2000 |
| Calidris_alpina | -0.669 | Calhim 2006 | 1.731 | Calhim 2006 |  | M | Pitcher 2005; Szekely 2000 |
| Calidris_bairdii | -0.771 | Calhim 2006 | 1.609 | Calhim 2006 |  | M | Pitcher 2005; Szekely 2000 |
| Calidris_canutus | -0.141 | Calhim 2006 | 2.094 | Calhim 2006 |  | M | Pitcher 2005; Szekely 2000 |
| Calidris_fuscicollis | -0.602 | Calhim 2006 | 1.617 | Calhim 2006 |  | PG | Pitcher 2005; Szekely 2000 |
| Micropalama_himantopus | -0.620 | Calhim 2006 | 1.716 | Calhim 2006 |  | M | Pitcher 2005; Szekely 2000 |
| Calidris_maritima | -0.719 | Calhim 2006 | 1.846 | Calhim 2006 |  | M | Pitcher 2005; Biagolini 2017; Szekely 2000 |
| Calidris_mauri | -0.310 | Hayward 2011 | 1.415 | Hayward 2011 |  | M | Garamszegi 2005; Pitcher 2005; Biagolini 2017; Szekely 2000 |
| Calidris_melanotos | -0.125 | Calhim 2006 | 1.993 | Calhim 2006 | Lekking | PG | Pitcher 2005; Holmes 1998; Szekely 2000 |
| Calidris_minuta | -0.540 | Calhim 2006 | 1.342 | Calhim 2006 |  | PA/PGA | Pitcher 2005 |
| Calidris_minutilla | -1.421 | Calhim 2006 | 1.362 | Calhim 2006 |  | M | Pitcher 2005; Szekely 2000 |
| Calidris_ptilocnemis | -0.427 | Calhim 2006 | 1.899 | Calhim 2006 |  | M | Pitcher 2005; Szekely 2000 |
| Calidris_pusilla | -0.310 | Hayward 2011 | 1.447 | Hayward 2011 |  | M | Garamszegi 2005; Pitcher 2005; Szekely 2000 |
| Calidris_ruficollis | -0.890 | Calhim 2006 | 1.519 | Calhim 2006 |  | M | Pitcher 2005; Szekely 2000 |
| Calidris_temminckii | -0.499 | Calhim 2006 | 1.415 | Calhim 2006 |  | PA/PGA | Pitcher 2005 |
| Callipepla_californica | 0.204 | Calhim 2006 | 2.255 | Calhim 2006 |  | M | Pitcher 2005 |
| Callipepla_gambelii | 0.155 | Calhim 2006 | 2.202 | Calhim 2006 |  | M | Pitcher 2005 |
| Callipepla_squamata | 0.152 | Calhim 2006 | 2.256 | Calhim 2006 |  | M | Pitcher 2005 |
| Callocephalon_fimbriatum | -0.959 | Calhim 2006 | 2.433 | Calhim 2006 |  | M | Pitcher 2005 |
| Calypte_anna | -2.000 | Calhim 2006 | 0.699 | Calhim 2006 | Lekking | PG | Pitcher 2005; Emlen 1977 |
| Calyptorhynchus_banksii | 0.093 | Calhim 2006 | 2.888 | Calhim 2006 |  | M | Pitcher 2005 |
| Campylorhynchus_brunneicapillus | -1.046 | Hayward 2011 | 1.590 | Hayward 2011 |  | M | Garamszegi 2005 |
| Capito_niger | -0.638 | Calhim 2006 | 1.845 | Calhim 2006 |  | M | Pitcher 2005 |
| Cardinalis_cardinalis | -0.347 | Calhim 2006 | 1.640 | Calhim 2006 |  | M | Pitcher 2005; Biagolini 2017 |
| Cardinalis_sinuatus | -0.745 | Calhim 2006 | 1.567 | Calhim 2006 |  | M | Pitcher 2005 |
| Carduelis_cannabina | -0.699 | Calhim 2006 | 1.279 | Calhim 2006 |  | M | Pitcher 2005; Biagolini 2017 |
| Carduelis_carduelis | -0.873 | Calhim 2006 | 1.204 | Calhim 2006 |  | M | Pitcher 2005 |
| Carduelis_chloris | -0.688 | Calhim 2006 | 1.436 | Calhim 2006 |  | M | Pitcher 2005 |
| Carduelis_flammea | -0.409 | Hayward 2011 | 1.079 | Hayward 2011 |  | M | Garamszegi 2005; Pitcher 2005 |
| Carduelis_hornemanni | -0.745 | Calhim 2006 | 1.146 | Calhim 2006 |  | M | Pitcher 2005 |
| Carduelis_pinus | -0.854 | Calhim 2006 | 1.111 | Calhim 2006 |  | M | Pitcher 2005 |
| Carduelis_psaltria | -1.000 | Calhim 2006 | 0.996 | Calhim 2006 |  | M | Pitcher 2005 |
| Carduelis_tristis | -0.638 | Calhim 2006 | 1.107 | Calhim 2006 |  | M | Pitcher 2005; Biagolini 2017 |
| Carpodacus_cassinii | -1.155 | Calhim 2006 | 1.430 | Calhim 2006 |  | M | Pitcher 2005 |
| Carpodacus_erythrinus | -0.620 | Calhim 2006 | 1.342 | Calhim 2006 |  | PG | Pitcher 2005; Biagolini 2017 |
| Carpodacus_mexicanus | -0.770 | Calhim 2006 | 1.330 | Calhim 2006 |  | PG | Pitcher 2005; Biagolini 2017 |
| Carpodacus_purpureus | -0.585 | Calhim 2006 | 1.377 | Calhim 2006 |  | M | Pitcher 2005 |
| Catharus_aurantiirostris | -0.602 | Calhim 2006 | 1.431 | Calhim 2006 |  | M | Pitcher 2005 |
| Catharus_fuscescens | -0.509 | Calhim 2006 | 1.490 | Calhim 2006 |  | M | Pitcher 2005 |
| Catharus_gracilirostris | -0.921 | Calhim 2006 | 1.286 | Calhim 2006 |  | M | Pitcher 2005 |
| Catharus_guttatus | -0.638 | Calhim 2006 | 1.436 | Calhim 2006 |  | M | Pitcher 2005 |
| Catharus_minimus | -0.292 | Calhim 2006 | 1.533 | Calhim 2006 |  | M | Pitcher 2005 |
| Catharus_occidentalis | -0.569 | Calhim 2006 | 1.377 | Calhim 2006 |  | M | Pitcher 2005 |
| Catharus_ustulatus | -0.420 | Calhim 2006 | 1.476 | Calhim 2006 |  | M | Pitcher 2005 |
| Centrocercus_urophasianus | 0.819 | Calhim 2006 | 3.492 | Calhim 2006 |  | PA/PGA | Biagolini 2017 |
| Centropus_phasianinus | 0.670 | Calhim 2006 | 2.521 | Calhim 2006 |  | M | Pitcher 2005; Biagolini 2017 |
| Cepphus_columba | -0.387 | Calhim 2006 | 2.687 | Calhim 2006 |  | M | Pitcher 2005 |
| Cepphus_grylle | 0.420 | Calhim 2006 | 2.599 | Calhim 2006 |  | M | Pitcher 2005; Biagolini 2017; Szekely 2000 |
| Cercomacra_tyrannina | -1.398 | Calhim 2006 | 1.250 | Calhim 2006 |  | M | Pitcher 2005; Biagolini 2017 |
| Certhionyx_variegatus | -0.602 | Calhim 2006 | 1.398 | Calhim 2006 |  | M | Pitcher 2005 |
| Ceryle_rudis | 0.000 | Hayward 2011 | 1.886 | Hayward 2011 |  | C | Pitcher 2005 |
| Chaetura_pelagica | -0.509 | Calhim 2006 | 1.286 | Calhim 2006 |  | C | Pitcher 2005 |
| Charadrius_bicinctus | -1.699 | Calhim 2006 | 1.793 | Calhim 2006 |  | M | Pitcher 2005 |
| Charadrius_dubius | -1.523 | Calhim 2006 | 1.602 | Calhim 2006 |  | M | Pitcher 2005; Szekely 2000 |
| Charadrius_hiaticula | -0.398 | Calhim 2006 | 1.732 | Calhim 2006 |  | M | Pitcher 2005 |
| Charadrius_marginatus | -1.699 | Calhim 2006 | 1.690 | Calhim 2006 |  | M | Pitcher 2005 |
| Charadrius_montanus | -0.409 | Calhim 2006 | 2.006 | Calhim 2006 |  | M | Pitcher 2005 |
| Charadrius_ruficapillus | -1.000 | Calhim 2006 | 1.560 | Calhim 2006 |  | M | Pitcher 2005 |
| Charadrius_semipalmatus | -1.000 | Calhim 2006 | 1.643 | Calhim 2006 |  | M | Pitcher 2005; Biagolini 2017 |
| Charadrius_vociferus | -0.745 | Calhim 2006 | 1.979 | Calhim 2006 |  | M | Pitcher 2005 |
| Anser_caerulescens | 1.243 | Calhim 2006 | 3.420 | Calhim 2006 |  | M | Pitcher 2005; Biagolini 2017 |
| Chenonetta_jubata | 0.310 | Calhim 2006 | 2.911 | Calhim 2006 |  | M | Pitcher 2005 |
| Chersomanes_albofasciata | -1.046 | Calhim 2006 | 1.438 | Calhim 2006 |  | M | Pitcher 2005 |
| Chionis_minor | 0.021 | Hayward 2011 | 2.704 | Hayward 2011 |  | M | Garamszegi 2005; Pitcher 2005 |
| Chiroxiphia_caudata | -0.921 | Calhim 2006 | 1.408 | Calhim 2006 | Lekking | PG | Pitcher 2005; Foster 1981 |
| Chiroxiphia_linearis | -0.481 | Calhim 2006 | 1.230 | Calhim 2006 | Lekking | PG | Pitcher 2005; Foster 1977 |
| Chiroxiphia_pareola | -0.854 | Calhim 2006 | 1.176 | Calhim 2006 | Lekking | PG | Pitcher 2005; Prum 1994 (Family is polygynous) |
| Chlamydera_cerviniventris | -0.495 | Calhim 2006 | 2.243 | Calhim 2006 | Lekking | PG | Pitcher 2005; Lenz 1994 |
| Chlamydera_maculata | 0.318 | Calhim 2006 | 2.152 | Calhim 2006 | Lekking | PG | Pitcher 2005; Lenz 1994 |
| Chlamydera_nuchalis | -0.469 | Calhim 2006 | 2.338 | Calhim 2006 | Lekking | PG | Pitcher 2005; Lenz 1994 |
| Chlidonias_niger | -2.000 | Calhim 2006 | 1.808 | Calhim 2006 |  | M | Pitcher 2005; Biagolini 2017 |
| Chloroceryle_americana | -1.046 | Calhim 2006 | 1.462 | Calhim 2006 |  | M | Pitcher 2005 |
| Chondestes_grammacus | -0.921 | Calhim 2006 | 1.481 | Calhim 2006 |  |  |  |
| Chordeiles_acutipennis | -1.000 | Calhim 2006 | 1.707 | Calhim 2006 |  | M | Pitcher 2005 |
| Chordeiles_minor | -1.097 | Calhim 2006 | 1.815 | Calhim 2006 |  | M | Pitcher 2005 |
| Chalcites_lucidus | -1.301 | Calhim 2006 | 1.398 | Calhim 2006 |  | M | Pitcher 2005 |
| Cicinnurus_regius | -0.495 | Calhim 2006 | 1.708 | Calhim 2006 | Lekking | PG | Pitcher 2005; Irestedt et al 2009 |
| Cincloramphus_mathewsi | -0.658 | Calhim 2006 | 1.477 | Calhim 2006 |  | PG | Pitcher 2005 |
| Cinclosoma_castaneothorax | -1.301 | Calhim 2006 | 1.851 | Calhim 2006 |  | M | Pitcher 2005 |
| Cinclosoma_cinnamomeum | -0.538 | Calhim 2006 | 1.785 | Calhim 2006 |  | M | Pitcher 2005 |
| Circus_aeruginosus | 0.483 | Calhim 2006 | 2.728 | Calhim 2006 |  | M | Pitcher 2005 |
| Cisticola_juncidis | -0.921 | Calhim 2006 | 1.204 | Calhim 2006 |  | PG | Pitcher 2005 |
| Cistothorus_palustris | -0.699 | Calhim 2006 | 1.075 | Calhim 2006 |  | PG | Pitcher 2005 |
| Cistothorus_platensis | -0.854 | Calhim 2006 | 0.954 | Calhim 2006 |  | PG | Pitcher 2005 |
| Clangula_hyemalis | 0.810 | Calhim 2006 | 2.924 | Calhim 2006 |  | M | Pitcher 2005 |
| Claravis_pretiosa | -0.387 | Calhim 2006 | 1.856 | Calhim 2006 |  | M | Pitcher 2005 |
| Climacteris_picumnus | -0.745 | Calhim 2006 | 1.525 | Calhim 2006 |  | C | Pitcher 2005 |
| Climacteris_rufus | -0.745 | Calhim 2006 | 1.544 | Calhim 2006 |  | C | Pitcher 2005 |
| Cnemophilus_loriae | -0.420 | Calhim 2006 | 1.908 | Calhim 2006 | Lekking | PG | Pitcher 2005; Beehler 1983 |
| Hesperiphona_vespertina | -0.444 | Calhim 2006 | 1.752 | Calhim 2006 |  | M | Pitcher 2005 |
| Coccyzus_americanus | -0.824 | Calhim 2006 | 1.744 | Calhim 2006 |  | M | Pitcher 2005 |
| Coccyzus_erythropthalmus | -0.602 | Calhim 2006 | 1.664 | Calhim 2006 |  | M | Pitcher 2005 |
| Cochlearius_cochlearius | 0.083 | Calhim 2006 | 2.778 | Calhim 2006 |  | M | Pitcher 2005 |
| Coeligena_coeligena | -1.699 | Calhim 2006 | 0.826 | Calhim 2006 |  | PG | Pitcher 2005 |
| Coereba_flaveola | -0.444 | Calhim 2006 | 1.000 | Calhim 2006 |  | M | Pitcher 2005 |
| Colaptes_auratus | -0.215 | Calhim 2006 | 2.109 | Calhim 2006 |  | PA/PGA | Pitcher 2005; Biagolini 2017 |
| Colinus_cristatus | 0.053 | Calhim 2006 | 2.137 | Calhim 2006 |  |  |  |
| Colinus_virginianus | -0.086 | Calhim 2006 | 2.226 | Calhim 2006 |  | PG | Pitcher 2005 |
| Colius_striatus | -0.854 | Calhim 2006 | 1.724 | Calhim 2006 |  | C | Pitcher 2005 |
| Aerodramus_spodiopygius | -0.886 | Calhim 2006 | 1.021 | Calhim 2006 |  | M | Pitcher 2005 |
| Colluricincla_harmonica | -0.143 | Calhim 2006 | 1.792 | Calhim 2006 |  | M | Pitcher 2005 |
| Colluricincla_megarhyncha | -0.387 | Calhim 2006 | 1.556 | Calhim 2006 |  | M | Pitcher 2005 |
| Colluricincla_woodwardi | -0.444 | Calhim 2006 | 1.732 | Calhim 2006 |  | M | Pitcher 2005 |
| Colonia_colonus | -0.585 | Calhim 2006 | 1.212 | Calhim 2006 |  | M | Pitcher 2005 |
| Columba_livia | 0.305 | Hayward 2011 | 2.484 | Hayward 2011 |  | M | Garamszegi 2005; Pitcher 2005 |
| Columba_palumbus | 0.230 | Calhim 2006 | 2.667 | Calhim 2006 |  | M | Pitcher 2005 |
| Columbina_passerina | -0.854 | Calhim 2006 | 1.540 | Calhim 2006 |  | M | Pitcher 2005 |
| Columbina_talpacoti | -0.959 | Calhim 2006 | 1.668 | Calhim 2006 |  | M | Pitcher 2005 |
| Conopophila_albogularis | -2.000 | Calhim 2006 | 1.114 | Calhim 2006 |  | M | Pitcher 2005 |
| Conopophila_rufogularis | -2.000 | Calhim 2006 | 1.061 | Calhim 2006 |  | C | Pitcher 2005 |
| Contopus_cooperi | -0.921 | Calhim 2006 | 1.502 | Calhim 2006 |  | M | Pitcher 2005 |
| Contopus_sordidulus | -1.222 | Calhim 2006 | 1.146 | Calhim 2006 |  | M | Pitcher 2005 |
| Contopus_virens | -1.046 | Calhim 2006 | 1.196 | Calhim 2006 |  | M | Pitcher 2005 |
| Coracina_boyeri | -1.046 | Calhim 2006 | 1.785 | Calhim 2006 |  | M | Pitcher 2005 |
| Coracina_caeruleogrisea | -0.357 | Calhim 2006 | 2.176 | Calhim 2006 |  | M | Pitcher 2005 |
| Coracina_longicauda | -0.959 | Calhim 2006 | 1.991 | Calhim 2006 |  | M | Pitcher 2005 |
| Coracina_montana | -1.301 | Calhim 2006 | 1.740 | Calhim 2006 |  | M | Pitcher 2005 |
| Coracina_novaehollandiae | -0.444 | Calhim 2006 | 2.021 | Calhim 2006 |  | M | Pitcher 2005 |
| Coracina_papuensis | -0.824 | Calhim 2006 | 1.813 | Calhim 2006 |  | M | Pitcher 2005 |
| Coracina_tenuirostris | -0.469 | Calhim 2006 | 1.875 | Calhim 2006 |  | M | Pitcher 2005 |
| Cormobates_leucophaeus | -0.886 | Calhim 2006 | 1.403 | Calhim 2006 |  | M | Pitcher 2005 |
| Corvus_brachyrhynchos | 0.057 | Calhim 2006 | 2.879 | Calhim 2006 |  | C | Biagolini 2017 |
| Corvus_corax | 0.297 | Calhim 2006 | 3.070 | Calhim 2006 |  | M | Pitcher 2005 |
| Corvus_corone | 0.243 | Hayward 2011 | 2.833 | Hayward 2011 |  | M | Pitcher 2005 |
| Corvus_coronoides | -1.699 | Calhim 2006 | 2.799 | Calhim 2006 |  | M | Pitcher 2005 |
| Corvus_frugilegus | 1.247 | Hayward 2011 | 2.728 | Hayward 2011 |  | M | Garamszegi 2005; Pitcher 2005 |
| Corvus_mellori | -1.398 | Calhim 2006 | 2.732 | Calhim 2006 |  | M | Pitcher 2005 |
| Corvus_monedula | 0.580 | Calhim 2006 | 2.369 | Calhim 2006 |  | M | Pitcher 2005; Biagolini 2017 |
| Corvus_splendens | 0.290 | Calhim 2006 | 2.279 | Calhim 2006 |  | M | Pitcher 2005 |
| Coturnix_chinensis | 0.250 | Calhim 2006 | 2.061 | Calhim 2006 |  |  |  |
| Coturnix_coturnix | 0.477 | Calhim 2006 | 2.111 | Calhim 2006 |  | M | Pitcher 2005 |
| Coturnix_japonica | 0.477 | Hayward 2011 | 2.111 | Hayward 2011 | Lekking | PG | Pitcher 2005; Domjan 1986 |
| Coturnix_pectoralis | 0.663 | Calhim 2006 | 2.000 | Calhim 2006 |  | M | Pitcher 2005 |
| Cracticus_nigrogularis | 0.220 | Calhim 2006 | 2.041 | Calhim 2006 |  | C | Pitcher 2005 |
| Crateroscelis_robusta | -0.921 | Calhim 2006 | 1.230 | Calhim 2006 |  | C | Pitcher 2005 |
| Crax_rubra | 0.772 | Calhim 2006 | 3.623 | Calhim 2006 |  | M | Pitcher 2005 |
| Crex_crex | 0.068 | Calhim 2006 | 2.176 | Calhim 2006 |  | M | Pitcher 2005 |
| Crotophaga_sulcirostris | -0.420 | Calhim 2006 | 1.934 | Calhim 2006 |  | C | Pitcher 2005 |
| Cyanerpes_cyaneus | -0.409 | Calhim 2006 | 1.130 | Calhim 2006 |  | M | Pitcher 2005 |
| Cyanocitta_cristata | -0.721 | Calhim 2006 | 1.964 | Calhim 2006 |  | M | Pitcher 2005 |
| Cyanocitta_stelleri | -0.462 | Calhim 2006 | 2.075 | Calhim 2006 |  | M | Pitcher 2005; Biagolini 2017 |
| Cyanocompsa_cyanoides | -0.444 | Calhim 2006 | 1.477 | Calhim 2006 |  | M | Pitcher 2005 |
| Cyanocorax_morio | 0.204 | Calhim 2006 | 2.386 | Calhim 2006 |  | C | Pitcher 2005; Biagolini 2017 |
| Cyanocorax_sanblasianus | -0.004 | Calhim 2006 | 1.863 | Calhim 2006 |  | C | Pitcher 2005 |
| Cyanocorax_yncas | -0.086 | Calhim 2006 | 1.863 | Calhim 2006 |  | C | Pitcher 2005 |
| Cygnus_atratus | 0.770 | Calhim 2006 | 3.792 | Calhim 2006 |  | M | Pitcher 2005; Biagolini 2017 |
| Cygnus_cygnus | 1.082 | Calhim 2006 | 4.021 | Calhim 2006 |  | M | Pitcher 2005 |
| Cygnus_melancoryphus | 0.787 | Calhim 2006 | 3.732 | Calhim 2006 |  | M | Pitcher 2005 |
| Cygnus_olor | 0.854 | Calhim 2006 | 4.072 | Calhim 2006 |  | M | Pitcher 2005 |
| Cyrtonyx_montezumae | -0.046 | Calhim 2006 | 2.277 | Calhim 2006 |  |  |  |
| Dacelo_leachi | 1.055 | Calhim 2006 | 2.493 | Calhim 2006 |  | C | Pitcher 2005 |
| Dacelo_novaeguineae | 0.314 | Calhim 2006 | 2.496 | Calhim 2006 |  | C | Griffith 2002; Pitcher 2005; Biagolini 2017 |
| Daphoenositta_chrysoptera | -0.602 | Calhim 2006 | 1.190 | Calhim 2006 |  | C | Pitcher 2005 |
| Dasyornis_brachypterus | -0.745 | Calhim 2006 | 1.623 | Calhim 2006 |  | M | Pitcher 2005 |
| Dasyornis_broadbenti | -0.409 | Calhim 2006 | 1.881 | Calhim 2006 |  | M | Pitcher 2005 |
| Delichon_urbicum | -0.699 | Calhim 2006 | 1.230 | Calhim 2006 |  | M | Pitcher 2005; Biagolini 2017 |
| Falcipennis_canadensis | -0.194 | Calhim 2006 | 2.740 | Calhim 2006 | Lekking |  | Pitcher 2005 |
| Dendragapus_obscurus | 0.450 | Calhim 2006 | 3.112 | Calhim 2006 | Lekking |  | Pitcher 2005 |
| Dendrocincla_fuliginosa | -0.538 | Calhim 2006 | 1.641 | Calhim 2006 |  | M | Pitcher 2005 |
| Dendrocygna_arcuata | 0.892 | Calhim 2006 | 2.937 | Calhim 2006 |  | M | Pitcher 2005 |
| Dendrocygna_bicolor | 0.678 | Calhim 2006 | 2.838 | Calhim 2006 |  | M | Pitcher 2005 |
| Dendrocygna_eytoni | 0.220 | Calhim 2006 | 2.896 | Calhim 2006 |  | M | Pitcher 2005 |
| Setophaga_adelaidae | -1.398 | Calhim 2006 | 1.406 | Calhim 2006 |  | M | Pitcher 2005 |
| Setophaga_caerulescens | -1.398 | Calhim 2006 | 0.991 | Calhim 2006 |  | PG | Pitcher 2005; Biagolini 2017 |
| Setophaga_castanea | -0.886 | Calhim 2006 | 1.090 | Calhim 2006 |  | M | Pitcher 2005 |
| Setophaga_cerulea | -1.000 | Calhim 2006 | 0.959 | Calhim 2006 |  | M | Pitcher 2005 |
| Setophaga_coronata | -0.620 | Calhim 2006 | 1.079 | Calhim 2006 |  | M | Pitcher 2005 |
| Setophaga_discolor | -0.602 | Calhim 2006 | 0.954 | Calhim 2006 |  | PG | Pitcher 2005 |
| Setophaga_fusca | -1.398 | Calhim 2006 | 1.000 | Calhim 2006 |  | M | Pitcher 2005 |
| Setophaga_graciae | -1.000 | Calhim 2006 | 0.903 | Calhim 2006 |  | M | Pitcher 2005 |
| Setophaga_magnolia | -1.155 | Calhim 2006 | 0.944 | Calhim 2006 |  | M | Pitcher 2005 |
| Setophaga_nigrescens | -1.046 | Calhim 2006 | 0.954 | Calhim 2006 |  | M | Pitcher 2005 |
| Setophaga_occidentalis | -1.046 | Calhim 2006 | 0.991 | Calhim 2006 |  | M | Pitcher 2005 |
| Setophaga_palmarum | -1.155 | Calhim 2006 | 1.013 | Calhim 2006 |  | PG | Pitcher 2005 |
| Setophaga_pensylvanica | -1.097 | Calhim 2006 | 0.982 | Calhim 2006 |  | M | Pitcher 2005; Biagolini 2017 |
| Setophaga_petechia | -0.770 | Calhim 2006 | 0.982 | Calhim 2006 |  | M | Pitcher 2005; Biagolini 2017 |
| Setophaga_pharetra | -1.046 | Calhim 2006 | 1.013 | Calhim 2006 |  | M | Pitcher 2005 |
| Setophaga_pinus | -1.000 | Calhim 2006 | 1.068 | Calhim 2006 |  | M | Pitcher 2005 |
| Setophaga_plumbea | -1.000 | Calhim 2006 | 1.041 | Calhim 2006 |  | M | Pitcher 2005 |
| Setophaga_striata | -0.886 | Calhim 2006 | 1.100 | Calhim 2006 |  | PG | Pitcher 2005 |
| Setophaga_tigrina | -0.886 | Calhim 2006 | 1.053 | Calhim 2006 |  | M | Pitcher 2005 |
| Setophaga_virens | -0.959 | Calhim 2006 | 0.964 | Calhim 2006 |  | M | Pitcher 2005 |
| Setophaga_vitellina | -0.959 | Calhim 2006 | 0.908 | Calhim 2006 |  | M | Pitcher 2005 |
| Dicaeum_hirundinaceum | -0.854 | Calhim 2006 | 0.929 | Calhim 2006 |  | M | Pitcher 2005 |
| Dicaeum_pectorale | -1.155 | Calhim 2006 | 0.740 | Calhim 2006 |  | M | Pitcher 2005 |
| Dicrurus_bracteatus | -1.699 | Calhim 2006 | 1.984 | Calhim 2006 |  | M | Pitcher 2005 |
| Dicrurus_hottentottus | -0.481 | Calhim 2006 | 1.914 | Calhim 2006 |  | M | Pitcher 2005 |
| Diomedea_exulans | 1.774 | Hayward 2011 | 3.934 | Hayward 2011 |  | M | Garamszegi 2005; Pitcher 2005; Biagolini 2017 |
| Dolichonyx_oryzivorus | -0.409 | Calhim 2006 | 1.672 | Calhim 2006 |  | PG | Pitcher 2005 |
| Dromaius_novaehollandiae | 2.103 | Hayward 2011 | 4.544 | Hayward 2011 |  | M | Garamszegi 2005 |
| Drymodes_brunneopygia | -0.456 | Calhim 2006 | 1.568 | Calhim 2006 |  | M | Pitcher 2005 |
| Dumetella_carolinensis | -0.658 | Calhim 2006 | 1.544 | Calhim 2006 |  | M | Pitcher 2005; Biagolini 2017 |
| Eclectus_roratus | 0.274 | Calhim 2006 | 2.631 | Calhim 2006 |  | C | Pitcher 2005 |
| Egretta_caerulea | -0.658 | Calhim 2006 | 2.561 | Calhim 2006 |  | M | Pitcher 2005 |
| Egretta_thula | -0.102 | Calhim 2006 | 2.568 | Calhim 2006 |  | M | Pitcher 2005 |
| Egretta_tricolor | 0.713 | Calhim 2006 | 2.648 | Calhim 2006 |  | M | Pitcher 2005 |
| Elaenia_flavogaster | -0.745 | Calhim 2006 | 1.380 | Calhim 2006 |  | M | Pitcher 2005; Biagolini 2017 |
| Elseyornis_melanops | -1.699 | Calhim 2006 | 1.477 | Calhim 2006 |  | M | Pitcher 2005 |
| Emberiza_bruniceps | -0.796 | Calhim 2006 | 1.415 | Calhim 2006 |  | M | Pitcher 2005 |
| Emberiza_citrinella | -0.292 | Calhim 2006 | 1.436 | Calhim 2006 |  | M | Pitcher 2005; Biagolini 2017 |
| Emberiza_melanocephala | -0.481 | Calhim 2006 | 1.447 | Calhim 2006 |  | M | Pitcher 2005 |
| Emberiza_schoeniclus | -0.282 | Hayward 2011 | 1.262 | Hayward 2011 |  | PG | Pitcher 2005; Biagolini 2017 |
| Emberizoides_herbicola | -0.824 | Calhim 2006 | 1.398 | Calhim 2006 |  | M | Pitcher 2005 |
| Empidonax_affinis | -0.886 | Calhim 2006 | 1.021 | Calhim 2006 |  | M | Pitcher 2005 |
| Empidonax_alnorum | -0.921 | Calhim 2006 | 1.121 | Calhim 2006 |  | M | Pitcher 2005 |
| Empidonax_atriceps | -1.222 | Calhim 2006 | 0.954 | Calhim 2006 |  | M | Pitcher 2005 |
| Empidonax_difficilis | -1.155 | Calhim 2006 | 1.000 | Calhim 2006 |  | M | Pitcher 2005 |
| Empidonax_flaviventris | -1.000 | Calhim 2006 | 1.075 | Calhim 2006 |  | M | Pitcher 2005 |
| Empidonax_hammondii | -1.155 | Calhim 2006 | 1.029 | Calhim 2006 |  | M | Pitcher 2005 |
| Empidonax_minimus | -0.721 | Calhim 2006 | 1.029 | Calhim 2006 |  | M | Pitcher 2005; Biagolini 2017 |
| Empidonax_traillii | -0.886 | Calhim 2006 | 1.114 | Calhim 2006 |  | PG | Pitcher 2005; Biagolini 2017 |
| Empidonax_virescens | -1.155 | Calhim 2006 | 1.117 | Calhim 2006 |  | M | Pitcher 2005; Biagolini 2017 |
| Empidonax_wrightii | -1.398 | Calhim 2006 | 1.097 | Calhim 2006 |  | M | Pitcher 2005 |
| Entomyzon_cyanotis | -0.032 | Calhim 2006 | 2.009 | Calhim 2006 |  | M | Pitcher 2005 |
| Eopsaltria_australis | -0.523 | Calhim 2006 | 1.279 | Calhim 2006 |  | C | Pitcher 2005 |
| Eopsaltria_georgiana | -1.301 | Calhim 2006 | 1.255 | Calhim 2006 |  | C | Pitcher 2005 |
| Epthianura_albifrons | -1.046 | Calhim 2006 | 1.079 | Calhim 2006 |  | M | Pitcher 2005 |
| Epthianura_aurifrons | -0.678 | Calhim 2006 | 1.000 | Calhim 2006 |  | M | Pitcher 2005 |
| Epthianura_crocea | -0.658 | Calhim 2006 | 0.903 | Calhim 2006 |  | M | Pitcher 2005 |
| Epthianura_tricolor | -1.097 | Hayward 2011 | 1.000 | Hayward 2011 |  | M | Garamszegi 2005; Pitcher 2005 |
| Eremalauda_starki | -1.222 | Calhim 2006 | 1.265 | Calhim 2006 |  | M | Pitcher 2005 |
| Eremophila_alpestris | -0.357 | Calhim 2006 | 1.513 | Calhim 2006 |  | M | Pitcher 2005 |
| Eremopterix_verticalis | -1.046 | Calhim 2006 | 1.225 | Calhim 2006 |  | M | Pitcher 2005 |
| Erithacus_rubecula | -0.638 | Calhim 2006 | 1.260 | Calhim 2006 |  |  |  |
| Charadrius_morinellus | 0.121 | Calhim 2006 | 2.053 | Calhim 2006 |  | PA/PGA | Pitcher 2005; Biagolini 2017; Szekely 2000 |
| Eudynamys_scolopaceus | -0.377 | Calhim 2006 | 2.377 | Calhim 2006 |  | M | Pitcher 2005 |
| Eudyptula_minor | 0.204 | Calhim 2006 | 3.069 | Calhim 2006 |  | M | Pitcher 2005 |
| Eulacestoma_nigropectus | -0.854 | Calhim 2006 | 1.290 | Calhim 2006 |  | M | Pitcher 2005 |
| Euphagus_carolinus | -0.538 | Calhim 2006 | 1.766 | Calhim 2006 |  | M | Pitcher 2005 |
| Euphagus_cyanocephalus | 0.093 | Calhim 2006 | 1.839 | Calhim 2006 |  | PG | Pitcher 2005 |
| Euplectes_orix | -0.268 | Calhim 2006 | 1.362 | Calhim 2006 |  | PG | Pitcher 2005; Biagolini 2017 |
| Eurypyga_helias | -0.620 | Calhim 2006 | 2.356 | Calhim 2006 |  | M | Pitcher 2005 |
| Falco_berigora | -0.509 | Calhim 2006 | 2.656 | Calhim 2006 |  | M | Pitcher 2005 |
| Falco_columbarius | -0.721 | Calhim 2006 | 2.238 | Calhim 2006 |  | M | Pitcher 2005; Biagolini 2017 |
| Falco_mexicanus | 0.597 | Calhim 2006 | 2.740 | Calhim 2006 |  | M | Pitcher 2005 |
| Falco_peregrinus | -1.046 | Calhim 2006 | 2.786 | Calhim 2006 |  | M | Pitcher 2005; Biagolini 2017 |
| Falco_sparverius | 0.324 | Calhim 2006 | 1.978 | Calhim 2006 |  | M | Pitcher 2005; Biagolini 2017 |
| Falco_tinnunculus | -0.143 | Hayward 2011 | 2.283 | Hayward 2011 |  | PA/PGA | Garamszegi 2005; Pitcher 2005; Biagolini 2017 |
| Falcunculus_frontatus | -0.481 | Calhim 2006 | 1.477 | Calhim 2006 |  | C | Pitcher 2005 |
| Ficedula_albicollis | -1.398 | Calhim 2006 | 1.013 | Calhim 2006 |  | PG | Pitcher 2005; Biagolini 2017 |
| Ficedula_hypoleuca | -1.398 | Hayward 2011 | 1.064 | Hayward 2011 |  | PG | Pitcher 2005; Biagolini 2017 |
| Formicarius_analis | -0.310 | Calhim 2006 | 1.769 | Calhim 2006 |  | M | Pitcher 2005 |
| Formicarius_nigricapillus | -0.796 | Calhim 2006 | 1.886 | Calhim 2006 |  | M | Pitcher 2005 |
| Francolinus_africanus | 0.204 | Calhim 2006 | 2.643 | Calhim 2006 |  |  |  |
| Francolinus_sephaena | 0.167 | Calhim 2006 | 2.602 | Calhim 2006 |  |  |  |
| Fratercula_arctica | 0.322 | Calhim 2006 | 2.653 | Calhim 2006 |  | M | Pitcher 2005; Biagolini 2017; Szekely 2000 |
| Fratercula_corniculata | -0.699 | Calhim 2006 | 2.804 | Calhim 2006 |  | M | Pitcher 2005 |
| Fregata_magnificens | 0.436 | Calhim 2006 | 3.233 | Calhim 2006 |  | M | Pitcher 2005 |
| Fringilla_coelebs | -0.357 | Hayward 2011 | 1.064 | Hayward 2011 |  | M | Pitcher 2005; Biagolini 2017 |
| Fulmarus_glacialis | 0.387 | Calhim 2006 | 2.914 | Calhim 2006 |  | M | Pitcher 2005; Biagolini 2017 |
| Galbula_albirostris | -0.959 | Calhim 2006 | 1.342 | Calhim 2006 |  | M | Pitcher 2005 |
| Galerida_cristata | -0.481 | Calhim 2006 | 1.602 | Calhim 2006 |  | M | Pitcher 2005 |
| Gallinago_media | 0.134 | Calhim 2006 | 2.196 | Calhim 2006 |  | PG | Szekely 2000 |
| Gallinula_chloropus | 0.167 | Calhim 2006 | 2.531 | Calhim 2006 |  | M | Pitcher 2005; Biagolini 2017 |
| Gallus_gallus | 1.447 | Hayward 2011 | 3.362 | Hayward 2011 | Lekking | PG | Garamszegi 2005; Pitcher 2005 |
| Gavia_adamsii | 0.121 | Calhim 2006 | 3.750 | Calhim 2006 |  | M | Pitcher 2005 |
| Gavia_arctica | -0.143 | Calhim 2006 | 3.382 | Calhim 2006 |  | M | Pitcher 2005 |
| Gavia_immer | 0.090 | Calhim 2006 | 3.737 | Calhim 2006 |  | M | Pitcher 2005; Biagolini 2017 |
| Gavia_stellata | -0.018 | Calhim 2006 | 3.277 | Calhim 2006 |  | M | Pitcher 2005 |
| Geococcyx_californianus | -0.444 | Calhim 2006 | 2.575 | Calhim 2006 |  | M | Pitcher 2005 |
| Geopelia_striata | -0.456 | Calhim 2006 | 1.699 | Calhim 2006 |  | M | Pitcher 2005 |
| Geothlypis_trichas | -0.678 | Calhim 2006 | 1.021 | Calhim 2006 |  | PG | Pitcher 2005; Biagolini 2017 |
| Gerygone_chloronota | -1.699 | Calhim 2006 | 0.806 | Calhim 2006 |  | M | Pitcher 2005 |
| Gerygone_chrysogaster | -1.097 | Calhim 2006 | 0.869 | Calhim 2006 |  | C | Pitcher 2005 |
| Gerygone_cinerea | -1.699 | Calhim 2006 | 0.845 | Calhim 2006 |  | M | Pitcher 2005 |
| Gerygone_fusca | -1.097 | Calhim 2006 | 0.857 | Calhim 2006 |  | M | Pitcher 2005 |
| Gerygone_levigaster | -0.602 | Calhim 2006 | 0.869 | Calhim 2006 |  | M | Pitcher 2005 |
| Gerygone_magnirostris | -0.721 | Calhim 2006 | 0.857 | Calhim 2006 |  | C | Pitcher 2005 |
| Gerygone_mouki | -0.538 | Calhim 2006 | 0.792 | Calhim 2006 |  | C | Pitcher 2005 |
| Gerygone_olivacea | -1.000 | Calhim 2006 | 0.892 | Calhim 2006 |  | M | Pitcher 2005 |
| Glycichaera_fallax | -1.301 | Calhim 2006 | 1.061 | Calhim 2006 |  | M | Pitcher 2005 |
| Grallina_cyanoleuca | 0.127 | Calhim 2006 | 1.677 | Calhim 2006 |  | M | Pitcher 2005; Biagolini 2017 |
| Grus_canadensis | 0.894 | Calhim 2006 | 3.653 | Calhim 2006 |  | M | Pitcher 2005; Biagolini 2017 |
| Grus_grus | 1.061 | Calhim 2006 | 3.699 | Calhim 2006 |  | M | Pitcher 2005 |
| Guira_guira | 0.243 | Calhim 2006 | 2.143 | Calhim 2006 |  | C | Pitcher 2005 |
| Gymnopithys_leucaspis | -1.097 | Hayward 2011 | 1.493 | Hayward 2011 |  | M | Garamszegi 2005; Pitcher 2005 |
| Gymnorhina_tibicen | 0.330 | Hayward 2011 | 2.496 | Hayward 2011 |  | C | Pitcher 2005; Biagolini 2017 |
| Gymnorhinus_cyanocephalus | 0.299 | Calhim 2006 | 2.033 | Calhim 2006 |  | M | Pitcher 2005 |
| Gypaetus_barbatus | 1.526 | Calhim 2006 | 3.653 | Calhim 2006 |  | M | Pitcher 2005 |
| Haematopus_bachmani | -0.319 | Calhim 2006 | 2.784 | Calhim 2006 |  | M | Pitcher 2005 |
| Haematopus_ostralegus | -0.268 | Calhim 2006 | 2.724 | Calhim 2006 |  | M | Pitcher 2005; Biagolini 2017; Szekely 2000 |
| Haematopus_palliatus | -0.509 | Calhim 2006 | 2.777 | Calhim 2006 |  | M | Pitcher 2005 |
| Haematopus_unicolor | -0.959 | Calhim 2006 | 2.857 | Calhim 2006 |  | M | Pitcher 2005 |
| Haliastur_sphenurus | -0.027 | Calhim 2006 | 2.854 | Calhim 2006 |  | M | Pitcher 2005 |
| Heliornis_fulica | -1.398 | Calhim 2006 | 2.130 | Calhim 2006 |  | M | Pitcher 2005 |
| Poecilodryas_albispecularis | -0.745 | Calhim 2006 | 1.568 | Calhim 2006 |  | M | Pitcher 2005 |
| Hieraaetus_morphnoides | -0.229 | Calhim 2006 | 2.826 | Calhim 2006 |  | M | Pitcher 2005 |
| Hirundo_neoxena | -0.824 | Calhim 2006 | 1.176 | Calhim 2006 |  | M | Pitcher 2005 |
| Hirundo_nigricans | -0.854 | Calhim 2006 | 1.155 | Calhim 2006 |  | M | Pitcher 2005 |
| Hirundo_rustica | -0.260 | Hayward 2011 | 1.279 | Hayward 2011 |  | PG | Pitcher 2005; Biagolini 2017 |
| Hirundo_tahitica | -1.046 | Calhim 2006 | 1.176 | Calhim 2006 |  | M | Pitcher 2005 |
| Histrionicus_histrionicus | 0.083 | Calhim 2006 | 2.815 | Calhim 2006 |  | M | Pitcher 2005 |
| Calamanthus_cautus | -0.854 | Calhim 2006 | 1.176 | Calhim 2006 |  | M | Pitcher 2005 |
| Hylophilus_decurtatus | -1.398 | Calhim 2006 | 1.000 | Calhim 2006 |  |  |  |
| Hylophilus_ochraceiceps | -1.699 | Calhim 2006 | 1.041 | Calhim 2006 |  | M | Pitcher 2005 |
| Hylophylax_naevioides | -1.000 | Hayward 2011 | 1.230 | Hayward 2011 |  | M | Garamszegi 2005 |
| Hymenolaimus_malacorhynchos | -0.076 | Calhim 2006 | 2.921 | Calhim 2006 |  | M | Pitcher 2005; Biagolini 2017 |
| Icteria_virens | -0.301 | Calhim 2006 | 1.408 | Calhim 2006 |  | M | Pitcher 2005 |
| Icterus_bullockii | -0.102 | Calhim 2006 | 1.582 | Calhim 2006 |  | M | Pitcher 2005 |
| Icterus_galbula | -0.161 | Calhim 2006 | 1.554 | Calhim 2006 |  | M | Pitcher 2005; Biagolini 2017 |
| Icterus_leucopteryx | -0.194 | Calhim 2006 | 1.677 | Calhim 2006 |  |  |  |
| Icterus_parisorum | -0.469 | Calhim 2006 | 1.579 | Calhim 2006 |  | M | Pitcher 2005 |
| Icterus_pectoralis | -0.444 | Calhim 2006 | 1.719 | Calhim 2006 |  | M | Pitcher 2005 |
| Icterus_spurius | -0.252 | Calhim 2006 | 1.326 | Calhim 2006 |  | M | Pitcher 2005 |
| Ixobrychus_exilis | -0.770 | Calhim 2006 | 1.889 | Calhim 2006 |  | M | Pitcher 2005 |
| Jacana_spinosa | -0.027 | Calhim 2006 | 1.954 | Calhim 2006 |  | PA/PGA | Pitcher 2005; Szekely 2000 |
| Junco_hyemalis | -0.538 | Hayward 2011 | 1.322 | Hayward 2011 |  | M | Garamszegi 2005; Pitcher 2005; Biagolini 2017 |
| Jynx_torquilla | -0.585 | Calhim 2006 | 1.491 | Calhim 2006 |  | M | Pitcher 2005; Biagolini 2017 |
| Lagopus_lagopus | 0.176 | Hayward 2011 | 2.845 | Hayward 2011 |  | PG | Pitcher 2005; Biagolini 2017 |
| Lagopus_leucura | -0.398 | Calhim 2006 | 2.555 | Calhim 2006 |  | M | Pitcher 2005; Biagolini 2017 |
| Lagopus_muta | 0.604 | Calhim 2006 | 2.681 | Calhim 2006 |  | M | Pitcher 2005 |
| Lalage_atrovirens | -1.097 | Calhim 2006 | 1.512 | Calhim 2006 |  | M | Pitcher 2005 |
| Lalage_tricolor | -1.097 | Calhim 2006 | 1.406 | Calhim 2006 |  | C | Pitcher 2005 |
| Lamprotornis_chalybaeus | -0.319 | Hayward 2011 | 1.968 | Hayward 2011 |  | M | Garamszegi 2005 |
| Lamprotornis_pulcher | -0.252 | Calhim 2006 | 1.996 | Calhim 2006 |  | C | Pitcher 2005 |
| Lamprotornis_purpuroptera | -0.959 | Hayward 2011 | 1.778 | Hayward 2011 |  | M | Garamszegi 2005 |
| Lanius_collaris | -0.678 | Hayward 2011 | 1.580 | Hayward 2011 |  | C | Pitcher 2005 |
| Lanius_collurio | -0.620 | Hayward 2011 | 1.450 | Hayward 2011 |  | C | Griffith 2002; Biagolini 2017 |
| Lanius_cristatus | -0.538 | Calhim 2006 | 1.477 | Calhim 2006 |  | M | Pitcher 2005 |
| Lanius_schach | -0.553 | Calhim 2006 | 1.556 | Calhim 2006 |  | M | Pitcher 2005 |
| Larus_argentatus | 0.398 | Calhim 2006 | 3.047 | Calhim 2006 |  | M | Pitcher 2005; Szekely 2000 |
| Larus_californicus | 0.589 | Calhim 2006 | 2.839 | Calhim 2006 |  | M | Pitcher 2005 |
| Larus_canus | 0.196 | Calhim 2006 | 2.636 | Calhim 2006 |  | M | Pitcher 2005; Biagolini 2017 |
| Larus_delawarensis | 0.274 | Calhim 2006 | 2.753 | Calhim 2006 |  | PG | Pitcher 2005 |
| Larus_fuscus | 0.322 | Calhim 2006 | 2.950 | Calhim 2006 |  | M | Pitcher 2005 |
| Larus_glaucescens | 0.346 | Calhim 2006 | 3.031 | Calhim 2006 |  | M | Pitcher 2005 |
| Larus_marinus | 0.577 | Calhim 2006 | 3.257 | Calhim 2006 |  | M | Pitcher 2005 |
| Larus_occidentalis | 0.307 | Hayward 2011 | 3.053 | Hayward 2011 |  | M | Garamszegi 2005; Biagolini 2017 |
| Chroicocephalus_ridibundus | -0.377 | Hayward 2011 | 2.439 | Hayward 2011 |  | M | Garamszegi 2005; Biagolini 2017 |
| Legatus_leucophaius | -1.398 | Calhim 2006 | 1.362 | Calhim 2006 |  | M | Pitcher 2005 |
| Lepidothrix_coronata | -0.796 | Calhim 2006 | 0.908 | Calhim 2006 | Lekking | PG | Pitcher 2005; Prum 1994 (Family is polygynous) |
| Leucosticte_arctoa | -0.523 | Calhim 2006 | 1.431 | Calhim 2006 |  | M | Pitcher 2005 |
| Lichenostomus_flavus | -2.000 | Calhim 2006 | 1.322 | Calhim 2006 |  | M | Pitcher 2005 |
| Lichenostomus_leucotis | -0.456 | Calhim 2006 | 1.398 | Calhim 2006 |  | M | Pitcher 2005 |
| Lichenostomus_ornatus | -0.523 | Calhim 2006 | 1.290 | Calhim 2006 |  | M | Pitcher 2005 |
| Lichenostomus_penicillatus | -0.824 | Hayward 2011 | 1.322 | Hayward 2011 |  | C | Pitcher 2005 |
| Lichenostomus_virescens | -0.678 | Calhim 2006 | 1.415 | Calhim 2006 |  | M | Pitcher 2005 |
| Limnodromus_griseus | -0.721 | Calhim 2006 | 2.049 | Calhim 2006 |  | M | Pitcher 2005 |
| Limnodromus_scolopaceus | -0.796 | Calhim 2006 | 2.000 | Calhim 2006 |  | M | Pitcher 2005 |
| Limnothlypis_swainsonii | -0.638 | Calhim 2006 | 1.133 | Calhim 2006 |  | M | Pitcher 2005 |
| Limosa_fedoa | -0.398 | Calhim 2006 | 2.497 | Calhim 2006 |  | M | Pitcher 2005 |
| Limosa_haemastica | -0.770 | Calhim 2006 | 2.403 | Calhim 2006 |  | M | Pitcher 2005 |
| Limosa_lapponica | -0.745 | Calhim 2006 | 2.490 | Calhim 2006 |  | M | Pitcher 2005 |
| Limosa_limosa | -0.328 | Calhim 2006 | 2.435 | Calhim 2006 |  | M | Pitcher 2005; Szekely 2000 |
| Loboparadisea_sericea | -0.328 | Calhim 2006 | 1.778 | Calhim 2006 | Lekking | PG | Pitcher 2005; Beehler 1983 |
| Locustella_fluviatilis | -0.658 | Calhim 2006 | 1.260 | Calhim 2006 |  |  |  |
| Locustella_luscinioides | -1.155 | Calhim 2006 | 1.176 | Calhim 2006 |  | M | Biagolini 2017 |
| Lonchura_castaneothorax | -1.046 | Calhim 2006 | 1.114 | Calhim 2006 |  | M | Pitcher 2005 |
| Lonchura_punctulata | -0.983 | Calhim 2006 | 1.146 | Calhim 2006 |  | PG | Pitcher 2005 |
| Lonchura_striata | -1.155 | Hayward 2011 | 1.053 | Hayward 2011 |  | M | Garamszegi 2005; Pitcher 2005 |
| Lophorina_superba | -0.167 | Calhim 2006 | 1.959 | Calhim 2006 | Lekking | PG | Pitcher 2005; Hoglund 1989; Irestedt et al 2009 |
| Lophura_leucomelanos | 0.555 | Calhim 2006 | 2.900 | Calhim 2006 |  | C | Biagolini 2017 |
| Loxia_curvirostra | -0.796 | Calhim 2006 | 1.633 | Calhim 2006 |  | M | Pitcher 2005; Biagolini 2017 |
| Loxia_leucoptera | -0.959 | Calhim 2006 | 1.444 | Calhim 2006 |  | M | Pitcher 2005 |
| Luscinia_svecica | -0.620 | Calhim 2006 | 1.230 | Calhim 2006 |  | M | Pitcher 2005; Biagolini 2017 |
| Malurus_amabilis | -0.678 | Calhim 2006 | 0.954 | Calhim 2006 |  | C | Pitcher 2005 |
| Malurus_coronatus | -1.301 | Calhim 2006 | 1.161 | Calhim 2006 |  | C | Pitcher 2005; Biagolini 2017 |
| Malurus_cyaneus | -0.319 | Hayward 2011 | 0.991 | Hayward 2011 |  | C | Griffith 2002; Pitcher 2005; Biagolini 2017 |
| Malurus_elegans | -0.337 | Calhim 2006 | 1.029 | Calhim 2006 |  | C | Pitcher 2005; Biagolini 2017 |
| Malurus_lamberti | -0.602 | Hayward 2011 | 0.892 | Hayward 2011 |  | C | Pitcher 2005 |
| Malurus_leucopterus | -0.481 | Hayward 2011 | 0.968 | Hayward 2011 |  | C | Pitcher 2005 |
| Malurus_melanocephalus | -0.310 | Calhim 2006 | 0.949 | Calhim 2006 |  | C | Pitcher 2005; Biagolini 2017 |
| Malurus_pulcherrimus | -0.745 | Calhim 2006 | 0.924 | Calhim 2006 |  | C | Pitcher 2005 |
| Malurus_splendens | -0.319 | Calhim 2006 | 1.000 | Calhim 2006 |  | C | Pitcher 2005; Biagolini 2017 |
| Manacus_manacus | -0.444 | Calhim 2006 | 1.146 | Calhim 2006 | Lekking | PG | Pitcher 2005; Prum 1994 (Family is polygynous) |
| Manacus_vitellinus | -0.854 | Hayward 2011 | 1.286 | Hayward 2011 | Lekking | PG | Prum 1994 |
| Manorina_flavigula | -0.420 | Hayward 2011 | 1.763 | Hayward 2011 |  | C | Pitcher 2005 |
| Manorina_melanocephala | -0.585 | Calhim 2006 | 1.748 | Calhim 2006 |  | C | Griffith 2002; Pitcher 2005; Biagolini 2017 |
| Manorina_melanophrys | -0.721 | Hayward 2011 | 1.477 | Hayward 2011 |  | C | Griffith 2002; Biagolini 2017 |
| Manucodia_ater | -0.886 | Calhim 2006 | 2.279 | Calhim 2006 |  | M | Pitcher 2005 |
| Manucodia_chalybatus | 0.380 | Calhim 2006 | 2.362 | Calhim 2006 |  | M | Pitcher 2005 |
| Manucodia_jobiensis | -0.959 | Calhim 2006 | 2.332 | Calhim 2006 |  | M | Pitcher 2005 |
| Manucodia_keraudrenii | 0.358 | Calhim 2006 | 2.207 | Calhim 2006 |  | M | Pitcher 2005 |
| Margarornis_squamiger | -1.523 | Calhim 2006 | 1.258 | Calhim 2006 |  | M | Pitcher 2005 |
| Megaceryle_alcyon | -0.770 | Calhim 2006 | 2.124 | Calhim 2006 |  | M | Pitcher 2005 |
| Megalurus_gramineus | -0.959 | Calhim 2006 | 1.255 | Calhim 2006 |  | M | Pitcher 2005 |
| Megapodius_reinwardt | 0.037 | Calhim 2006 | 3.041 | Calhim 2006 |  | M | Pitcher 2005 |
| Melanerpes_aurifrons | -0.409 | Calhim 2006 | 1.937 | Calhim 2006 |  | M | Pitcher 2005 |
| Melanerpes_carolinus | -0.770 | Calhim 2006 | 1.859 | Calhim 2006 |  | M | Pitcher 2005 |
| Melanerpes_erythrocephalus | -1.222 | Calhim 2006 | 1.862 | Calhim 2006 |  | M | Pitcher 2005 |
| Melanerpes_formicivorus | -0.032 | Calhim 2006 | 1.806 | Calhim 2006 |  | C | Griffith 2002; Pitcher 2005; Biagolini 2017 |
| Melanerpes_herminieri | -1.097 | Calhim 2006 | 2.000 | Calhim 2006 |  | M | Pitcher 2005 |
| Melanitta_fusca | 0.236 | Calhim 2006 | 3.302 | Calhim 2006 |  | M | Pitcher 2005 |
| Melanitta_nigra | 0.653 | Calhim 2006 | 2.978 | Calhim 2006 |  | M | Pitcher 2005 |
| Melanocharis_nigra | -0.921 | Calhim 2006 | 1.000 | Calhim 2006 |  | M | Pitcher 2005 |
| Melanocharis_versteri | -1.222 | Calhim 2006 | 1.041 | Calhim 2006 |  | M | Pitcher 2005 |
| Melanodryas_cucullata | -0.357 | Calhim 2006 | 1.322 | Calhim 2006 |  | C | Pitcher 2005 |
| Melanodryas_vittata | -0.420 | Calhim 2006 | 1.423 | Calhim 2006 |  | C | Pitcher 2005 |
| Meleagris_gallopavo | 1.447 | Hayward 2011 | 4.204 | Hayward 2011 | Lekking | PG | Garamszegi 2005; Pitcher 2005; Beletsky 1995 |
| Melidectes_belfordi | -0.143 | Calhim 2006 | 1.914 | Calhim 2006 |  | C | Pitcher 2005 |
| Melidectes_ochromelas | -0.745 | Calhim 2006 | 1.839 | Calhim 2006 |  | M | Pitcher 2005 |
| Melidectes_torquatus | -0.337 | Calhim 2006 | 1.699 | Calhim 2006 |  | M | Pitcher 2005 |
| Melilestes_megarhynchus | -0.310 | Calhim 2006 | 1.643 | Calhim 2006 |  | M | Pitcher 2005 |
| Meliphaga_analoga | -0.796 | Calhim 2006 | 1.322 | Calhim 2006 |  | M | Pitcher 2005 |
| Meliphaga_gracilis | -1.699 | Calhim 2006 | 1.161 | Calhim 2006 |  | M | Pitcher 2005 |
| Meliphaga_lewinii | -0.310 | Calhim 2006 | 1.602 | Calhim 2006 |  | M | Pitcher 2005 |
| Meliphaga_montana | -0.638 | Calhim 2006 | 1.415 | Calhim 2006 |  | M | Pitcher 2005 |
| Meliphaga_notata | -0.745 | Calhim 2006 | 1.398 | Calhim 2006 |  | M | Pitcher 2005 |
| Melipotes_fumigatus | -0.824 | Calhim 2006 | 1.690 | Calhim 2006 |  | M | Pitcher 2005 |
| Melithreptus_albogularis | -1.155 | Calhim 2006 | 1.079 | Calhim 2006 |  | C | Pitcher 2005 |
| Melithreptus_brevirostris | -0.409 | Calhim 2006 | 1.260 | Calhim 2006 |  | C | Pitcher 2005 |
| Melithreptus_lunatus | -0.721 | Calhim 2006 | 1.267 | Calhim 2006 |  | C | Pitcher 2005 |
| Melopsittacus_undulatus | -0.569 | Calhim 2006 | 1.505 | Calhim 2006 |  | M | Pitcher 2005 |
| Melospiza_georgiana | -0.161 | Calhim 2006 | 1.255 | Calhim 2006 |  | M | Pitcher 2005; Biagolini 2017 |
| Melospiza_lincolnii | -0.328 | Calhim 2006 | 1.241 | Calhim 2006 |  | M | Pitcher 2005 |
| Melospiza_melodia | -0.420 | Hayward 2011 | 1.322 | Hayward 2011 |  | PG | Pitcher 2005; Biagolini 2017 |
| Melozone_kieneri | -0.237 | Calhim 2006 | 1.678 | Calhim 2006 |  | M | Pitcher 2005 |
| Menura_novaehollandiae | 0.364 | Calhim 2006 | 3.072 | Calhim 2006 | Lekking |  | Pitcher 2005 |
| Mergus_merganser | 0.720 | Calhim 2006 | 3.233 | Calhim 2006 |  | M | Pitcher 2005 |
| Mergus_serrator | 1.124 | Calhim 2006 | 3.056 | Calhim 2006 |  | M | Pitcher 2005 |
| Merops_apiaster | -0.854 | Calhim 2006 | 1.753 | Calhim 2006 |  | C | Pitcher 2005 |
| Merops_ornatus | -2.000 | Calhim 2006 | 1.459 | Calhim 2006 |  | C | Pitcher 2005 |
| Microcerculus_marginatus | -0.796 | Calhim 2006 | 1.258 | Calhim 2006 |  |  |  |
| Microeca_fascinans | -0.237 | Calhim 2006 | 1.079 | Calhim 2006 |  | M | Pitcher 2005 |
| Microeca_flavigaster | -0.721 | Calhim 2006 | 1.130 | Calhim 2006 |  | M | Pitcher 2005 |
| Microeca_papuana | -0.310 | Calhim 2006 | 1.021 | Calhim 2006 |  | C | Pitcher 2005 |
| Miliaria_calandra | -0.337 | Calhim 2006 | 1.681 | Calhim 2006 |  | PG | Pitcher 2005; Biagolini 2017 |
| Milvus_migrans | 0.516 | Calhim 2006 | 2.791 | Calhim 2006 |  | M | Pitcher 2005 |
| Mimus_polyglottos | -0.602 | Calhim 2006 | 1.699 | Calhim 2006 |  | M | Garamszegi 2005; Pitcher 2005 |
| Mino_dumontii | -0.387 | Calhim 2006 | 2.447 | Calhim 2006 |  | C | Pitcher 2005 |
| Mionectes_macconnelli | -0.796 | Calhim 2006 | 1.176 | Calhim 2006 | Lekking |  | Pitcher 2005 |
| Mionectes_rufiventris | -1.699 | Calhim 2006 | 1.146 | Calhim 2006 | Lekking |  | Pitcher 2005 |
| Mniotilta_varia | -1.097 | Calhim 2006 | 0.996 | Calhim 2006 |  | M | Pitcher 2005 |
| Molothrus_ater | -0.056 | Hayward 2011 | 1.643 | Hayward 2011 |  | M | Garamszegi 2005; Pitcher 2005 |
| Molothrus_bonariensis | -0.538 | Calhim 2006 | 1.592 | Calhim 2006 |  | M | Pitcher 2005 |
| Monarcha_axillaris | -0.824 | Calhim 2006 | 1.061 | Calhim 2006 |  | M | Pitcher 2005 |
| Monarcha_chrysomela | -0.745 | Calhim 2006 | 1.146 | Calhim 2006 |  | M | Pitcher 2005 |
| Monarcha_guttula | -0.244 | Calhim 2006 | 1.161 | Calhim 2006 |  | M | Pitcher 2005 |
| Monarcha_melanopsis | -0.398 | Calhim 2006 | 1.362 | Calhim 2006 |  | M | Pitcher 2005 |
| Monarcha_trivirgatus | -0.337 | Calhim 2006 | 1.130 | Calhim 2006 |  | M | Pitcher 2005 |
| Motacilla_alba | 0.041 | Calhim 2006 | 1.362 | Calhim 2006 |  | M | Pitcher 2005 |
| Motacilla_flava | -0.432 | Calhim 2006 | 1.230 | Calhim 2006 |  | M | Pitcher 2005 |
| Myiagra_alecto | -1.699 | Calhim 2006 | 1.322 | Calhim 2006 |  | M | Pitcher 2005 |
| Myiagra_inquieta | -0.444 | Calhim 2006 | 1.097 | Calhim 2006 |  | M | Pitcher 2005 |
| Myiagra_rubecula | -0.377 | Calhim 2006 | 1.146 | Calhim 2006 |  | C | Pitcher 2005 |
| Myiarchus_cinerascens | -0.602 | Calhim 2006 | 1.462 | Calhim 2006 |  | M | Pitcher 2005 |
| Myiarchus_crinitus | -0.409 | Calhim 2006 | 1.552 | Calhim 2006 |  | M | Pitcher 2005 |
| Myiarchus_tuberculifer | -0.745 | Calhim 2006 | 1.299 | Calhim 2006 |  | M | Pitcher 2005 |
| Myiarchus_tyrannulus | -0.387 | Calhim 2006 | 1.599 | Calhim 2006 |  | M | Pitcher 2005 |
| Myiobius_barbatus | -1.301 | Calhim 2006 | 1.079 | Calhim 2006 | Lekking |  | Pitcher 2005 |
| Myioborus_miniatus | -1.046 | Calhim 2006 | 1.146 | Calhim 2006 |  | M | Pitcher 2005 |
| Myioborus_pictus | -0.721 | Calhim 2006 | 0.973 | Calhim 2006 |  | PG | Pitcher 2005 |
| Myiodynastes_maculatus | -0.481 | Calhim 2006 | 1.633 | Calhim 2006 |  | M | Pitcher 2005 |
| Myiopagis_gaimardii | -1.301 | Calhim 2006 | 1.079 | Calhim 2006 |  | M | Pitcher 2005 |
| Myiopagis_viridicata | -1.398 | Calhim 2006 | 1.146 | Calhim 2006 |  | M | Pitcher 2005 |
| Myzomela_erythrocephala | -0.770 | Calhim 2006 | 0.929 | Calhim 2006 |  | M | Pitcher 2005 |
| Myzomela_obscura | -1.046 | Calhim 2006 | 1.079 | Calhim 2006 |  | M | Pitcher 2005 |
| Myzomela_rosenbergii | -0.921 | Calhim 2006 | 0.875 | Calhim 2006 |  | M | Pitcher 2005 |
| Myzomela_sanguinolenta | -0.824 | Calhim 2006 | 0.845 | Calhim 2006 |  | M | Pitcher 2005 |
| Nectarinia_jugularis | -0.824 | Calhim 2006 | 0.929 | Calhim 2006 |  | M | Pitcher 2005 |
| Neochmia_modesta | -1.398 | Calhim 2006 | 1.124 | Calhim 2006 |  | M | Pitcher 2005 |
| Neochmia_phaeton | -1.301 | Calhim 2006 | 0.903 | Calhim 2006 |  | M | Pitcher 2005 |
| Neochmia_ruficauda | -1.155 | Calhim 2006 | 1.000 | Calhim 2006 |  | M | Pitcher 2005 |
| Neochmia_temporalis | -1.523 | Calhim 2006 | 0.954 | Calhim 2006 |  | M | Pitcher 2005 |
| Ninox_boobook | -0.824 | Calhim 2006 | 2.395 | Calhim 2006 |  | M | Pitcher 2005 |
| Notharchus_macrorhynchos | -1.097 | Calhim 2006 | 2.079 | Calhim 2006 |  | M | Pitcher 2005 |
| Nothoprocta_cinerascens | 0.496 | Calhim 2006 | 3.000 | Calhim 2006 |  | PA/PGA | Pitcher 2005 |
| Numenius_phaeopus | -0.456 | Calhim 2006 | 2.591 | Calhim 2006 |  | M | Pitcher 2005 |
| Numida_meleagris | 0.577 | Calhim 2006 | 3.267 | Calhim 2006 |  | M | Pitcher 2005 |
| Nyctibius_griseus | -0.585 | Calhim 2006 | 2.384 | Calhim 2006 |  | M | Pitcher 2005 |
| Nycticorax_nycticorax | 0.037 | Calhim 2006 | 2.960 | Calhim 2006 |  | M | Pitcher 2005 |
| Nymphicus_hollandicus | -1.155 | Calhim 2006 | 1.929 | Calhim 2006 |  | M | Pitcher 2005 |
| Oceanodroma_leucorhoa | -1.699 | Calhim 2006 | 1.500 | Calhim 2006 |  | M | Pitcher 2005; Biagolini 2017 |
| Oceanodroma_melania | -0.469 | Calhim 2006 | 1.694 | Calhim 2006 |  | M | Pitcher 2005 |
| Ocyphaps_lophotes | 0.097 | Calhim 2006 | 2.316 | Calhim 2006 |  | M | Pitcher 2005 |
| Oenanthe_oenanthe | -0.149 | Calhim 2006 | 1.415 | Calhim 2006 |  | PG | Pitcher 2005; Biagolini 2017 |
| Onychorhynchus_coronatus | -0.745 | Calhim 2006 | 1.270 | Calhim 2006 |  | M | Pitcher 2005 |
| Geothlypis_formosa | -0.638 | Calhim 2006 | 1.121 | Calhim 2006 |  | M | Pitcher 2005 |
| Geothlypis_philadelphia | -0.824 | Calhim 2006 | 1.075 | Calhim 2006 |  | M | Pitcher 2005 |
| Geothlypis_tolmiei | -0.824 | Calhim 2006 | 1.025 | Calhim 2006 |  | M | Pitcher 2005 |
| Oreoica_gutturalis | -0.208 | Calhim 2006 | 1.799 | Calhim 2006 |  | M | Pitcher 2005 |
| Oreopsar_bolivianus | -0.066 | Calhim 2006 | 1.868 | Calhim 2006 |  | M | Pitcher 2005 |
| Oreortyx_pictus | 0.029 | Calhim 2006 | 2.378 | Calhim 2006 |  |  |  |
| Oriolus_flavocinctus | 0.572 | Calhim 2006 | 2.121 | Calhim 2006 |  | M | Pitcher 2005 |
| Oriolus_sagittatus | 0.000 | Calhim 2006 | 1.964 | Calhim 2006 |  | M | Pitcher 2005 |
| Oriolus_szalayi | -0.745 | Calhim 2006 | 2.021 | Calhim 2006 |  | M | Pitcher 2005 |
| Ortalis_motmot | -0.131 | Calhim 2006 | 2.626 | Calhim 2006 |  | M | Pitcher 2005 |
| Ortalis_vetula | 0.299 | Calhim 2006 | 2.822 | Calhim 2006 |  | M | Pitcher 2005 |
| Orthonyx_spaldingii | -1.301 | Calhim 2006 | 2.185 | Calhim 2006 |  | M | Pitcher 2005 |
| Orthonyx_temminckii | -1.523 | Calhim 2006 | 1.819 | Calhim 2006 |  | C | Pitcher 2005 |
| Oxyura_australis | 1.311 | Calhim 2006 | 2.910 | Calhim 2006 | Lekking | M | Pitcher 2005; Scott 1990 |
| Oxyura_maccoa | -0.959 | Calhim 2006 | 2.825 | Calhim 2006 | Lekking | M | Pitcher 2005; Scott 1990 |
| Pachycephala_inornata | -0.347 | Calhim 2006 | 1.556 | Calhim 2006 |  | M | Pitcher 2005 |
| Pachycephala_olivacea | -0.222 | Calhim 2006 | 1.602 | Calhim 2006 |  | M | Pitcher 2005 |
| Pachycephala_pectoralis | -0.229 | Calhim 2006 | 1.431 | Calhim 2006 |  | M | Pitcher 2005; Biagolini 2017 |
| Pachycephala_rufiventris | -1.000 | Hayward 2011 | 1.301 | Hayward 2011 |  | M | Garamszegi 2005; Pitcher 2005 |
| Pachycephala_schlegelii | -0.337 | Calhim 2006 | 1.279 | Calhim 2006 |  | M | Pitcher 2005 |
| Pachycephala_simplex | -0.745 | Calhim 2006 | 1.267 | Calhim 2006 |  | M | Pitcher 2005 |
| Pachycephala_soror | -0.244 | Calhim 2006 | 1.371 | Calhim 2006 |  | M | Pitcher 2005 |
| Pandion_haliaetus | 1.247 | Calhim 2006 | 3.154 | Calhim 2006 |  | M | Pitcher 2005 |
| Panurus_biarmicus | -0.561 | Calhim 2006 | 1.196 | Calhim 2006 |  | M | Pitcher 2005; Biagolini 2017 |
| Paradisaea_apoda | -0.131 | Calhim 2006 | 2.398 | Calhim 2006 | Lekking | PG | Pitcher 2005; Hoglund 1989; Irestedt et al 2009 |
| Paradisaea_guilielmi | -0.125 | Calhim 2006 | 2.403 | Calhim 2006 | Lekking | PG | Pitcher 2005; Hoglund 1989; Irestedt et al 2009 |
| Paradisaea_minor | -0.018 | Calhim 2006 | 2.358 | Calhim 2006 | Lekking | PG | Pitcher 2005; Hoglund 1989; Irestedt et al 2009 |
| Paradisaea_raggiana | -0.004 | Calhim 2006 | 2.476 | Calhim 2006 | Lekking | PG | Pitcher 2005; Hoglund 1989; Irestedt et al 2009 |
| Paradisaea_rudolphi | -0.260 | Calhim 2006 | 2.207 | Calhim 2006 | Lekking | PG | Pitcher 2005; Irestedt et al 2009 |
| Pardalotus_rubricatus | -2.000 | Calhim 2006 | 1.079 | Calhim 2006 |  | M | Pitcher 2005 |
| Pardalotus_striatus | -1.699 | Calhim 2006 | 1.053 | Calhim 2006 |  | C | Pitcher 2005 |
| Paroaria_coronata | -0.252 | Calhim 2006 | 1.599 | Calhim 2006 |  | M | Pitcher 2005 |
| Parotia_lawesii | -0.229 | Calhim 2006 | 2.201 | Calhim 2006 | Lekking | PG | Pitcher 2005; Hoglund 1989; Irestedt et al 2009 |
| Parotia_wahnesi | -0.237 | Calhim 2006 | 2.190 | Calhim 2006 | Lekking | PG | Pitcher 2005; Hoglund 1989; Irestedt et al 2009 |
| Setophaga_americana | -0.678 | Calhim 2006 | 0.851 | Calhim 2006 |  | M | Pitcher 2005 |
| Oreothlypis_gutturalis | -0.824 | Calhim 2006 | 0.978 | Calhim 2006 |  | M | Pitcher 2005 |
| Oreothlypis_superciliosa | -1.222 | Calhim 2006 | 0.954 | Calhim 2006 |  | M | Pitcher 2005 |
| Poecile_atricapilla | -0.569 | Calhim 2006 | 1.104 | Calhim 2006 |  | M | Pitcher 2005; Biagolini 2017 |
| Poecile_carolinensis | -0.658 | Calhim 2006 | 1.037 | Calhim 2006 |  | M | Pitcher 2005 |
| Poecile_gambeli | -0.854 | Calhim 2006 | 1.079 | Calhim 2006 |  | M | Pitcher 2005 |
| Poecile_hudsonica | -0.770 | Calhim 2006 | 1.045 | Calhim 2006 |  | M | Pitcher 2005 |
| Parus_major | -0.886 | Hayward 2011 | 1.279 | Hayward 2011 |  | M | Garamszegi 2005; Pitcher 2005; Biagolini 2017 |
| Poecile_montanus | -0.959 | Hayward 2011 | 1.009 | Hayward 2011 |  | M | Garamszegi 2005; Pitcher 2005; Biagolini 2017 |
| Poecile_sclateri | -0.678 | Calhim 2006 | 1.041 | Calhim 2006 |  | M | Pitcher 2005 |
| Passer_domesticus | -0.377 | Hayward 2011 | 1.447 | Hayward 2011 |  | M | Pitcher 2005; Biagolini 2017 |
| Passer_hispaniolensis | -0.097 | Calhim 2006 | 1.431 | Calhim 2006 |  | M | Pitcher 2005 |
| Passer_montanus | -0.509 | Calhim 2006 | 1.342 | Calhim 2006 |  | M | Pitcher 2005; Biagolini 2017 |
| Passerculus_sandwichensis | -0.310 | Calhim 2006 | 1.276 | Calhim 2006 |  | PG | Pitcher 2005; Biagolini 2017 |
| Passerella_iliaca | -0.222 | Calhim 2006 | 1.550 | Calhim 2006 |  | M | Pitcher 2005 |
| Passerina_caerulea | -0.481 | Calhim 2006 | 1.210 | Calhim 2006 |  | M | Pitcher 2005; Biagolini 2017 |
| Passerina_ciris | -0.387 | Calhim 2006 | 1.161 | Calhim 2006 |  | PG | Pitcher 2005 |
| Passerina_cyanea | -0.252 | Calhim 2006 | 1.173 | Calhim 2006 |  | PG | Pitcher 2005; Biagolini 2017 |
| Passerina_versicolor | -0.854 | Calhim 2006 | 1.461 | Calhim 2006 |  | M | Pitcher 2005 |
| Patagioenas_fasciata | 0.248 | Calhim 2006 | 2.515 | Calhim 2006 |  | M | Pitcher 2005 |
| Patagioenas_leucocephala | 0.117 | Calhim 2006 | 2.420 | Calhim 2006 |  | M | Pitcher 2005 |
| Patagioenas_speciosa | 0.491 | Calhim 2006 | 2.418 | Calhim 2006 |  | M | Pitcher 2005 |
| Pavo_cristatus | 0.975 | Calhim 2006 | 3.680 | Calhim 2006 |  |  |  |
| Pelagodroma_marina | -0.854 | Calhim 2006 | 1.604 | Calhim 2006 |  | M | Pitcher 2005 |
| Pelecanoides_urinatrix | 0.580 | Calhim 2006 | 2.043 | Calhim 2006 |  | M | Pitcher 2005 |
| Peltops_blainvillii | -1.523 | Calhim 2006 | 1.544 | Calhim 2006 |  | M | Pitcher 2005 |
| Penelopina_nigra | 0.328 | Calhim 2006 | 2.949 | Calhim 2006 |  | PG | Pitcher 2005 |
| Peneothello_cyanus | -0.260 | Calhim 2006 | 1.389 | Calhim 2006 |  | C | Pitcher 2005 |
| Peneothello_sigillata | -1.301 | Calhim 2006 | 1.314 | Calhim 2006 |  | M | Pitcher 2005 |
| Perdicula_asiatica | -0.036 | Calhim 2006 | 1.903 | Calhim 2006 |  | M | Pitcher 2005 |
| Perdix_perdix | -0.244 | Hayward 2011 | 2.602 | Hayward 2011 |  | M | Garamszegi 2005 |
| Petrochelidon_pyrrhonota | -0.420 | Calhim 2006 | 1.334 | Calhim 2006 |  | M | Pitcher 2005 |
| Petroica_goodenovii | -0.886 | Calhim 2006 | 0.903 | Calhim 2006 |  | M | Pitcher 2005; Biagolini 2017 |
| Petroica_multicolor | -0.796 | Calhim 2006 | 1.079 | Calhim 2006 |  | M | Pitcher 2005 |
| Petroica_rodinogaster | -0.553 | Calhim 2006 | 0.991 | Calhim 2006 |  | M | Pitcher 2005 |
| Petroica_rosea | -0.638 | Calhim 2006 | 0.935 | Calhim 2006 |  | M | Pitcher 2005 |
| Pezopetes_capitalis | -0.022 | Calhim 2006 | 1.747 | Calhim 2006 |  | M | Pitcher 2005 |
| Phaethornis_superciliosus | -1.222 | Calhim 2006 | 0.699 | Calhim 2006 | Lekking |  | Pitcher 2005 |
| Phalacrocorax_aristotelis | 0.893 | Calhim 2006 | 3.288 | Calhim 2006 |  | M | Pitcher 2005 |
| Phalacrocorax_sulcirostris | 0.000 | Calhim 2006 | 3.041 | Calhim 2006 |  | M | Pitcher 2005 |
| Phalaropus_fulicaria | 0.212 | Hayward 2011 | 1.663 | Hayward 2011 |  | PA/PGA | Garamszegi 2005; Griffith 2002; Pitcher 2005; Biagolini 2017 |
| Phalaropus_lobatus | -0.347 | Hayward 2011 | 1.544 | Hayward 2011 |  | PA/PGA | Garamszegi 2005; Pitcher 2005; Biagolini 2017 |
| Phaps_chalcoptera | 0.322 | Calhim 2006 | 2.494 | Calhim 2006 |  | M | Pitcher 2005 |
| Pharomachrus_auriceps | -0.215 | Calhim 2006 | 2.248 | Calhim 2006 |  | M | Pitcher 2005 |
| Phasianus_colchicus | 0.914 | Hayward 2011 | 3.120 | Hayward 2011 | Lekking | PG | Pitcher 2005 |
| Pheucticus_ludovicianus | -0.347 | Calhim 2006 | 1.659 | Calhim 2006 |  | M | Pitcher 2005 |
| Pheucticus_melanocephalus | -0.201 | Calhim 2006 | 1.643 | Calhim 2006 |  | M | Pitcher 2005 |
| Philemon_argenticeps | -1.301 | Calhim 2006 | 1.987 | Calhim 2006 |  | M | Pitcher 2005 |
| Philemon_buceroides | -0.301 | Calhim 2006 | 2.201 | Calhim 2006 |  | M | Pitcher 2005 |
| Philemon_citreogularis | -0.658 | Calhim 2006 | 1.724 | Calhim 2006 |  | C | Pitcher 2005 |
| Philemon_corniculatus | 0.057 | Calhim 2006 | 2.049 | Calhim 2006 |  | M | Pitcher 2005 |
| Philemon_meyeri | -0.620 | Calhim 2006 | 1.653 | Calhim 2006 |  | M | Pitcher 2005 |
| Philomachus_pugnax | 0.657 | Calhim 2006 | 2.230 | Calhim 2006 | Lekking | PG | Pitcher 2005; Beehler 1988; Szekely 2000 |
| Phoenicurus_ochruros | -0.824 | Calhim 2006 | 1.217 | Calhim 2006 |  | M | Biagolini 2017 |
| Phoenicurus_phoenicurus | -1.000 | Calhim 2006 | 1.146 | Calhim 2006 |  | PG | Pitcher 2005; Biagolini 2017 |
| Phylidonyris_albifrons | -0.602 | Hayward 2011 | 1.262 | Hayward 2011 |  | M | Garamszegi 2005 |
| Phylidonyris_melanops | -0.585 | Calhim 2006 | 1.246 | Calhim 2006 |  | M | Pitcher 2005 |
| Phylidonyris_novaehollandiae | -0.678 | Calhim 2006 | 1.362 | Calhim 2006 |  | C | Pitcher 2005 |
| Phylidonyris_pyrrhoptera | -0.620 | Calhim 2006 | 1.279 | Calhim 2006 |  | M | Pitcher 2005; Biagolini 2017 |
| Phylloscopus_collybita | -0.721 | Calhim 2006 | 0.903 | Calhim 2006 |  | M | Pitcher 2005 |
| Phylloscopus_fuscatus | -1.114 | Calhim 2006 | 0.982 | Calhim 2006 |  | PG | Biagolini 2017 |
| Phylloscopus_sibilatrix | -0.796 | Calhim 2006 | 0.954 | Calhim 2006 |  | PG | Pitcher 2005; Biagolini 2017 |
| Phylloscopus_trochilus | -0.745 | Calhim 2006 | 0.954 | Calhim 2006 |  | PG | Pitcher 2005; Biagolini 2017 |
| Pica_pica | 0.097 | Calhim 2006 | 2.342 | Calhim 2006 |  | M | Pitcher 2005 |
| Picoides_arcticus | -1.699 | Calhim 2006 | 1.857 | Calhim 2006 |  | M | Pitcher 2005 |
| Picoides_pubescens | -1.046 | Calhim 2006 | 1.431 | Calhim 2006 |  | M | Pitcher 2005 |
| Picoides_scalaris | -1.398 | Calhim 2006 | 1.481 | Calhim 2006 |  | M | Pitcher 2005 |
| Picoides_villosus | -1.398 | Calhim 2006 | 1.845 | Calhim 2006 |  | M | Pitcher 2005 |
| Pipilo_chlorurus | -1.097 | Calhim 2006 | 1.468 | Calhim 2006 |  | M | Pitcher 2005 |
| Pipilo_crissalis | -0.174 | Calhim 2006 | 1.732 | Calhim 2006 |  | M | Pitcher 2005 |
| Pipilo_erythrophthalmus | -0.745 | Hayward 2011 | 1.594 | Hayward 2011 |  | M | Garamszegi 2005; Pitcher 2005 |
| Pipilo_fuscus | -0.060 | Calhim 2006 | 1.660 | Calhim 2006 |  | M | Pitcher 2005 |
| Pipilo_maculatus | -0.347 | Calhim 2006 | 1.584 | Calhim 2006 |  | M | Pitcher 2005; Biagolini 2017 |
| Pipilo_ocai | -0.585 | Calhim 2006 | 1.810 | Calhim 2006 |  |  |  |
| Pipra_erythrocephala | -0.523 | Calhim 2006 | 1.041 | Calhim 2006 | Lekking | PG | Pitcher 2005; Prum 1994 (Family is polygynous) |
| Pipra_filicauda | -0.959 | Calhim 2006 | 1.140 | Calhim 2006 | Lekking | PG | Pitcher 2005; Prum 1994 (Family is polygynous) |
| Pipra_mentalis | -1.398 | Calhim 2006 | 1.176 | Calhim 2006 | Lekking | PG | Pitcher 2005; Prum 1994 (Family is polygynous) |
| Piranga_bidentata | -0.301 | Calhim 2006 | 1.540 | Calhim 2006 |  | M | Pitcher 2005 |
| Piranga_erythrocephala | -0.409 | Calhim 2006 | 1.338 | Calhim 2006 |  | M | Pitcher 2005 |
| Piranga_flava | -0.585 | Calhim 2006 | 1.580 | Calhim 2006 |  | M | Pitcher 2005 |
| Piranga_ludoviciana | -0.377 | Calhim 2006 | 1.449 | Calhim 2006 |  | M | Pitcher 2005 |
| Piranga_olivacea | -0.292 | Calhim 2006 | 1.462 | Calhim 2006 |  | M | Pitcher 2005; Biagolini 2017 |
| Piranga_rubra | -0.319 | Calhim 2006 | 1.473 | Calhim 2006 |  | M | Pitcher 2005 |
| Pitohui_ferrugineus | 0.236 | Calhim 2006 | 1.954 | Calhim 2006 |  | C | Pitcher 2005 |
| Pitohui_nigrescens | -0.620 | Calhim 2006 | 1.792 | Calhim 2006 |  | M | Pitcher 2005 |
| Platycercus_elegans | -0.432 | Calhim 2006 | 2.127 | Calhim 2006 |  | M | Pitcher 2005 |
| Plectorhyncha_lanceolata | -1.301 | Calhim 2006 | 1.602 | Calhim 2006 |  |  |  |
| Plectrophenax_nivalis | 0.000 | Hayward 2011 | 1.519 | Hayward 2011 |  | M | Garamszegi 2005; Pitcher 2005; Biagolini 2017 |
| Plocepasser_mahali | -0.509 | Hayward 2011 | 1.643 | Hayward 2011 |  | C | Biagolini 2017 |
| Ploceus_cucullatus | -0.444 | Calhim 2006 | 1.602 | Calhim 2006 |  | PG | Pitcher 2005 |
| Ploceus_philippinus | -0.161 | Hayward 2011 | 1.398 | Hayward 2011 |  | PG | Pitcher 2005 |
| Pluvialis_apricaria | -0.201 | Calhim 2006 | 2.267 | Calhim 2006 |  | M | Pitcher 2005; Szekely 2000 |
| Pluvialis_dominica | -1.097 | Calhim 2006 | 2.161 | Calhim 2006 |  | M | Pitcher 2005; Biagolini 2017 |
| Pluvialis_squatarola | -1.301 | Calhim 2006 | 2.342 | Calhim 2006 |  | M | Pitcher 2005 |
| Podargus_strigoides | -0.569 | Calhim 2006 | 2.448 | Calhim 2006 |  | M | Pitcher 2005 |
| Podiceps_nigricollis | -0.721 | Calhim 2006 | 2.465 | Calhim 2006 |  | M | Pitcher 2005 |
| Podilymbus_podiceps | -0.036 | Calhim 2006 | 2.649 | Calhim 2006 |  | M | Pitcher 2005 |
| Poecilodryas_superciliosa | -0.959 | Calhim 2006 | 1.301 | Calhim 2006 |  | M | Pitcher 2005 |
| Poephila_cincta | -0.824 | Calhim 2006 | 1.218 | Calhim 2006 |  | M | Pitcher 2005 |
| Polioptila_caerulea | -1.222 | Calhim 2006 | 0.778 | Calhim 2006 |  | M | Pitcher 2005 |
| Polioptila_melanura | -1.301 | Calhim 2006 | 0.708 | Calhim 2006 |  | M | Pitcher 2005 |
| Pomatostomus_halli | -0.959 | Calhim 2006 | 1.613 | Calhim 2006 |  | C | Pitcher 2005 |
| Pomatostomus_ruficeps | -0.745 | Hayward 2011 | 1.701 | Hayward 2011 |  | C | Pitcher 2005 |
| Pomatostomus_superciliosus | -0.745 | Hayward 2011 | 1.623 | Hayward 2011 |  | C | Pitcher 2005 |
| Pomatostomus_temporalis | -0.268 | Calhim 2006 | 2.083 | Calhim 2006 |  | C | Pitcher 2005; Biagolini 2017 |
| Pooecetes_gramineus | -0.222 | Hayward 2011 | 1.431 | Hayward 2011 |  | M | Garamszegi 2005; Pitcher 2005 |
| Porphyrio_porphyrio | 0.865 | Calhim 2006 | 2.826 | Calhim 2006 |  | C | Griffith 2002; Pitcher 2005; Biagolini 2017 |
| Progne_chalybea | -0.004 | Calhim 2006 | 1.623 | Calhim 2006 |  | M | Pitcher 2005 |
| Progne_subis | -0.328 | Calhim 2006 | 1.708 | Calhim 2006 |  | PG | Pitcher 2005; Biagolini 2017 |
| Progne_tapera | -0.387 | Calhim 2006 | 1.549 | Calhim 2006 |  | M | Pitcher 2005 |
| Prosthemadera_novaeseelandiae | 0.161 | Calhim 2006 | 2.097 | Calhim 2006 |  | M | Biagolini 2017 |
| Protonotaria_citrea | -0.921 | Calhim 2006 | 1.124 | Calhim 2006 |  | M | Pitcher 2005 |
| Prunella_collaris | 0.483 | Calhim 2006 | 1.673 | Calhim 2006 |  | PA/PGA | Griffith 2002; Biagolini 2017 |
| Prunella_modularis | -0.161 | Calhim 2006 | 1.318 | Calhim 2006 |  | PA/PGA | Griffith 2002; Pitcher 2005; Biagolini 2017 |
| Psarocolius_decumanus | -0.143 | Calhim 2006 | 2.484 | Calhim 2006 |  | PG | Pitcher 2005 |
| Psarocolius_montezuma | -1.301 | Calhim 2006 | 2.603 | Calhim 2006 |  | PG | Pitcher 2005 |
| Psarocolius_wagleri | 0.204 | Calhim 2006 | 2.313 | Calhim 2006 |  | PG | Pitcher 2005 |
| Psephotus_haematonotus | -0.602 | Calhim 2006 | 1.813 | Calhim 2006 |  | M | Pitcher 2005 |
| Psittacula_krameri | -0.357 | Hayward 2011 | 2.107 | Hayward 2011 |  | M | Garamszegi 2005 |
| Psophodes_olivaceus | -0.276 | Calhim 2006 | 1.778 | Calhim 2006 |  | M | Pitcher 2005 |
| Pteridophora_alberti | -0.036 | Calhim 2006 | 1.949 | Calhim 2006 | Lekking | PG | Pitcher 2005; Irestedt et al 2009 |
| Ptilinopus_magnificus | 0.294 | Calhim 2006 | 2.681 | Calhim 2006 |  | M | Pitcher 2005 |
| Ptilinopus_regina | -0.553 | Calhim 2006 | 2.041 | Calhim 2006 |  | M | Pitcher 2005 |
| Ptilinopus_superbus | 0.155 | Calhim 2006 | 2.101 | Calhim 2006 |  | M | Pitcher 2005 |
| Ptilonorhynchus_violaceus | -0.959 | Hayward 2011 | 2.238 | Hayward 2011 | Lekking | PG | Garamszegi 2005; Pitcher 2005; Lenz 1994 |
| Ptiloprora_guisei | -0.770 | Calhim 2006 | 1.322 | Calhim 2006 |  | M | Pitcher 2005 |
| Ptiloris_magnificus | 0.212 | Calhim 2006 | 2.260 | Calhim 2006 | Lekking | PG | Pitcher 2005; Irestedt et al 2009 |
| Ptilorrhoa_caerulescens | -1.046 | Calhim 2006 | 1.740 | Calhim 2006 |  | M | Pitcher 2005 |
| Puffinus_tenuirostris | 0.146 | Calhim 2006 | 2.748 | Calhim 2006 |  | M | Pitcher 2005; Biagolini 2017 |
| Pycnonotus_barbatus | -0.215 | Calhim 2006 | 1.587 | Calhim 2006 |  | M | Pitcher 2005 |
| Pycnoptilus_floccosus | -1.046 | Calhim 2006 | 1.491 | Calhim 2006 |  | M | Pitcher 2005 |
| Pycnopygius_cinereus | -0.367 | Calhim 2006 | 1.690 | Calhim 2006 |  | M | Pitcher 2005 |
| Pygochelidon_cyanoleuca | -0.699 | Calhim 2006 | 1.033 | Calhim 2006 |  | M | Pitcher 2005 |
| Pygoscelis_adeliae | 1.292 | Hayward 2011 | 3.699 | Hayward 2011 |  | M | Garamszegi 2005; Pitcher 2005; Biagolini 2017 |
| Pygoscelis_papua | 1.292 | Hayward 2011 | 3.792 | Hayward 2011 |  | M | Garamszegi 2005; Pitcher 2005 |
| Pyrocephalus_rubinus | -0.638 | Calhim 2006 | 1.149 | Calhim 2006 |  | M | Pitcher 2005; Biagolini 2017 |
| Pyrrholaemus_brunneus | -1.097 | Calhim 2006 | 1.041 | Calhim 2006 |  | M | Pitcher 2005 |
| Pyrrhula_pyrrhula | -1.284 | Calhim 2006 | 1.290 | Calhim 2006 |  |  |  |
| Quelea_quelea | -0.523 | Calhim 2006 | 1.228 | Calhim 2006 |  | M | Biagolini 2017 |
| Quiscalus_major | 0.305 | Calhim 2006 | 2.292 | Calhim 2006 |  | PG | Pitcher 2005 |
| Quiscalus_mexicanus | 0.508 | Calhim 2006 | 2.345 | Calhim 2006 |  | PG | Pitcher 2005 |
| Quiscalus_quiscula | 0.220 | Calhim 2006 | 2.092 | Calhim 2006 |  | M | Pitcher 2005 |
| Rallus_elegans | -0.143 | Calhim 2006 | 2.618 | Calhim 2006 |  | M | Pitcher 2005 |
| Rallus_limicola | -0.284 | Calhim 2006 | 1.949 | Calhim 2006 |  | M | Pitcher 2005 |
| Ramphocaenus_melanurus | -0.745 | Calhim 2006 | 1.017 | Calhim 2006 |  | M | Pitcher 2005 |
| Ramphocelus_passerinii | -0.137 | Calhim 2006 | 1.504 | Calhim 2006 |  | M | Pitcher 2005 |
| Regulus_calendula | -0.921 | Calhim 2006 | 0.839 | Calhim 2006 |  | M | Pitcher 2005 |
| Regulus_satrapa | -0.854 | Calhim 2006 | 0.771 | Calhim 2006 |  | M | Pitcher 2005 |
| Rhipidura_albolimbata | -1.046 | Calhim 2006 | 0.929 | Calhim 2006 |  | M | Pitcher 2005 |
| Rhipidura_atra | -0.959 | Calhim 2006 | 1.033 | Calhim 2006 |  | M | Pitcher 2005 |
| Rhipidura_brachyrhyncha | -0.658 | Calhim 2006 | 0.903 | Calhim 2006 |  | M | Pitcher 2005 |
| Rhipidura_fuliginosa | -0.398 | Calhim 2006 | 0.968 | Calhim 2006 |  | M | Pitcher 2005; Biagolini 2017 |
| Rhipidura_hyperythra | -1.046 | Calhim 2006 | 1.041 | Calhim 2006 |  | M | Pitcher 2005 |
| Rhipidura_leucophrys | -0.131 | Calhim 2006 | 1.301 | Calhim 2006 |  | M | Pitcher 2005 |
| Rhipidura_leucothorax | -0.456 | Calhim 2006 | 1.352 | Calhim 2006 |  | M | Pitcher 2005 |
| Rhipidura_rufidorsa | -0.620 | Calhim 2006 | 0.699 | Calhim 2006 |  | M | Pitcher 2005 |
| Rhipidura_rufiventris | -0.137 | Calhim 2006 | 1.161 | Calhim 2006 |  | M | Pitcher 2005 |
| Rhipidura_threnothorax | -1.523 | Calhim 2006 | 1.230 | Calhim 2006 |  | M | Pitcher 2005 |
| Riparia_riparia | -0.620 | Calhim 2006 | 1.146 | Calhim 2006 |  | M | Pitcher 2005; Biagolini 2017 |
| Rissa_tridactyla | -0.155 | Calhim 2006 | 2.591 | Calhim 2006 |  | M | Pitcher 2005; Biagolini 2017 |
| Rostratula_benghalensis | 0.513 | Calhim 2006 | 2.097 | Calhim 2006 |  | PA/PGA | Pitcher 2005; Szekely 2000 |
| Rupicola_peruviana | -0.018 | Calhim 2006 | 2.452 | Calhim 2006 | Lekking |  | Pitcher 2005 |
| Saltator_maximus | -0.337 | Calhim 2006 | 1.672 | Calhim 2006 |  | M | Pitcher 2005 |
| Sappho_sparganura | -1.699 | Calhim 2006 | 0.740 | Calhim 2006 |  | PG | Pitcher 2005 |
| Saxicola_caprata | -1.222 | Calhim 2006 | 1.250 | Calhim 2006 |  | M | Pitcher 2005 |
| Saxicola_rubetra | -0.921 | Calhim 2006 | 1.220 | Calhim 2006 |  |  |  |
| Saxicola_torquata | -0.523 | Hayward 2011 | 1.204 | Hayward 2011 |  | M | Garamszegi 2005; Pitcher 2005 |
| Sayornis_nigricans | -1.398 | Calhim 2006 | 1.290 | Calhim 2006 |  | M | Pitcher 2005 |
| Sayornis_saya | -0.658 | Calhim 2006 | 1.326 | Calhim 2006 |  | M | Pitcher 2005 |
| Schoeniophylax_phryganophilus | -0.509 | Calhim 2006 | 1.276 | Calhim 2006 |  | M | Pitcher 2005 |
| Scolopax_minor | -0.060 | Calhim 2006 | 2.188 | Calhim 2006 | Lekking | PG | Pitcher 2005; Szekely 2000 |
| Seiurus_aurocapilla | -0.699 | Calhim 2006 | 1.276 | Calhim 2006 |  | M | Pitcher 2005 |
| Sericornis_citreogularis | -0.824 | Calhim 2006 | 1.176 | Calhim 2006 |  | M | Pitcher 2005 |
| Sericornis_frontalis | -0.432 | Calhim 2006 | 1.041 | Calhim 2006 |  | C | Griffith 2002; Pitcher 2005; Biagolini 2017 |
| Sericornis_magnirostris | -0.398 | Calhim 2006 | 1.000 | Calhim 2006 |  | C | Pitcher 2005 |
| Sericornis_nouhuysi | -0.276 | Calhim 2006 | 1.114 | Calhim 2006 |  | C | Pitcher 2005 |
| Sericornis_papuensis | -1.155 | Calhim 2006 | 0.929 | Calhim 2006 |  | M | Pitcher 2005 |
| Sericornis_perspicillatus | -0.569 | Calhim 2006 | 0.778 | Calhim 2006 |  | C | Pitcher 2005 |
| Sericulus_chrysocephalus | -0.086 | Calhim 2006 | 1.869 | Calhim 2006 | Lekking | PG | Pitcher 2005; Lenz 1994 |
| Serinus_canaria | -0.638 | Hayward 2011 | 1.114 | Hayward 2011 |  | M | Garamszegi 2005; Pitcher 2005; Biagolini 2017 |
| Serinus_serinus | -0.824 | Calhim 2006 | 1.049 | Calhim 2006 |  | M | Biagolini 2017 |
| Setophaga_ruticilla | -0.553 | Calhim 2006 | 0.935 | Calhim 2006 |  | M | Pitcher 2005; Biagolini 2017 |
| Sialia_currucoides | -0.180 | Calhim 2006 | 1.471 | Calhim 2006 |  | M | Pitcher 2005; Biagolini 2017 |
| Sialia_mexicana | -0.699 | Calhim 2006 | 1.462 | Calhim 2006 |  | C | Griffith 2002; Biagolini 2017 |
| Sialia_sialis | -0.577 | Calhim 2006 | 1.491 | Calhim 2006 |  | M | Pitcher 2005; Biagolini 2017 |
| Sicalis_flaveola | -0.444 | Calhim 2006 | 1.320 | Calhim 2006 |  |  |  |
| Sitta_canadensis | -0.959 | Calhim 2006 | 0.991 | Calhim 2006 |  | M | Pitcher 2005 |
| Sitta_carolinensis | -1.000 | Calhim 2006 | 1.324 | Calhim 2006 |  | M | Pitcher 2005 |
| Sitta_pusilla | -0.886 | Calhim 2006 | 1.004 | Calhim 2006 |  | C | Pitcher 2005; Biagolini 2017 |
| Sitta_pygmaea | -1.523 | Calhim 2006 | 1.025 | Calhim 2006 |  | C | Pitcher 2005 |
| Smicrornis_brevirostris | -1.301 | Calhim 2006 | 0.708 | Calhim 2006 |  | C | Pitcher 2005 |
| Somateria_mollissima | 0.732 | Calhim 2006 | 3.325 | Calhim 2006 |  | M | Pitcher 2005 |
| Spheniscus_magellanicus | 0.922 | Hayward 2011 | 3.650 | Hayward 2011 |  | M | Garamszegi 2005 |
| Sphyrapicus_varius | -1.046 | Calhim 2006 | 1.677 | Calhim 2006 |  | M | Pitcher 2005 |
| Spiza_americana | -0.187 | Calhim 2006 | 1.447 | Calhim 2006 |  | PG | Pitcher 2005; Biagolini 2017 |
| Spizella_arborea | -0.538 | Hayward 2011 | 1.305 | Hayward 2011 |  | M | Garamszegi 2005; Pitcher 2005 |
| Spizella_atrogularis | -1.000 | Calhim 2006 | 1.083 | Calhim 2006 |  | M | Pitcher 2005 |
| Spizella_breweri | -0.678 | Calhim 2006 | 1.037 | Calhim 2006 |  | M | Pitcher 2005 |
| Spizella_pallida | -0.699 | Calhim 2006 | 1.041 | Calhim 2006 |  | M | Pitcher 2005 |
| Spizella_passerina | -0.745 | Calhim 2006 | 1.093 | Calhim 2006 |  | PG | Pitcher 2005 |
| Spizella_pusilla | -0.796 | Calhim 2006 | 1.121 | Calhim 2006 |  | PG | Pitcher 2005 |
| Stagonopleura_guttata | -1.046 | Calhim 2006 | 1.176 | Calhim 2006 |  | M | Pitcher 2005 |
| Phalaropus_tricolor | -0.114 | Hayward 2011 | 1.714 | Hayward 2011 |  | PA/PGA | Garamszegi 2005; Pitcher 2005; Biagolini 2017 |
| Stercorarius_longicaudus | -0.770 | Calhim 2006 | 2.447 | Calhim 2006 |  | M | Pitcher 2005 |
| Stercorarius_parasiticus | 0.544 | Calhim 2006 | 2.642 | Calhim 2006 |  | M | Pitcher 2005 |
| Stercorarius_pomarinus | 0.013 | Calhim 2006 | 2.812 | Calhim 2006 |  | M | Pitcher 2005 |
| Sterna_forsteri | -0.921 | Calhim 2006 | 2.199 | Calhim 2006 |  | M | Pitcher 2005 |
| Sterna_hirundo | -1.046 | Calhim 2006 | 2.079 | Calhim 2006 |  | M | Pitcher 2005; Biagolini 2017; Szekely 2000 |
| Sterna_paradisaea | -0.959 | Calhim 2006 | 2.041 | Calhim 2006 |  | M | Pitcher 2005 |
| Streptopelia_chinensis | -0.187 | Calhim 2006 | 2.185 | Calhim 2006; Dunn 2001 |  | M | Pitcher 2005 |
| Streptopelia_senegalensis | -0.041 | Calhim 2006 | 2.000 | Dunn 2001 | Body size incorrect in original source (Peter Dunn, personal communication) | M | Pitcher 2005 |
| Stiltia_isabella | -0.745 | Calhim 2006 | 1.813 | Calhim 2006 |  | M | Pitcher 2005; Szekely 2000 |
| Streptopelia_decaocto | -0.086 | Hayward 2011 | 2.161 | Hayward 2011 |  | M | Garamszegi 2005; Pitcher 2005 |
| Streptopelia_roseogrisea | -0.155 | Hayward 2011 | 2.053 | Hayward 2011 |  | M | Garamszegi 2005 |
| Cypseloides_rutilus | -0.229 | Calhim 2006 | 2.025 | Calhim 2006 |  | M | Pitcher 2005 |
| Strix_aluco | 0.505 | Calhim 2006 | 2.623 | Calhim 2006 |  | M | Pitcher 2005; Biagolini 2017 |
| Strix_nebulosa | 0.565 | Calhim 2006 | 2.878 | Calhim 2006 |  | M | Pitcher 2005 |
| Strix_uralensis | 0.398 | Calhim 2006 | 2.794 | Calhim 2006 |  | M | Pitcher 2005 |
| Ciccaba_virgata | -0.432 | Calhim 2006 | 2.398 | Calhim 2006 |  | M | Pitcher 2005 |
| Struthidea_cinerea | -0.125 | Calhim 2006 | 2.107 | Calhim 2006 |  | C | Pitcher 2005 |
| Struthio_camelus | 2.062 | Hayward 2011 | 5.000 | Hayward 2011 |  | PG | Pitcher 2005 |
| Sturnella_magna | 0.025 | Calhim 2006 | 1.934 | Calhim 2006 |  | PG | Pitcher 2005 |
| Sturnella_militaris | -0.523 | Calhim 2006 | 1.735 | Calhim 2006 |  |  |  |
| Sturnella_neglecta | 0.179 | Hayward 2011 | 1.934 | Hayward 2011 |  | PG | Pitcher 2005 |
| Sturnus_roseus | -0.409 | Calhim 2006 | 1.906 | Calhim 2006 |  | M | Pitcher 2005 |
| Sturnus_vulgaris | -0.347 | Hayward 2011 | 1.928 | Hayward 2011 |  | PG | Pitcher 2005; Biagolini 2017 |
| Surnia_ulula | 0.204 | Calhim 2006 | 2.519 | Calhim 2006 |  | M | Pitcher 2005 |
| Sylvia_atricapilla | -0.097 | Calhim 2006 | 1.230 | Calhim 2006 |  | M | Pitcher 2005 |
| Sylvia_communis | -0.357 | Calhim 2006 | 1.176 | Calhim 2006 |  | PG | Pitcher 2005 |
| Sylvia_curruca | -0.357 | Calhim 2006 | 1.004 | Calhim 2006 |  |  |  |
| Synthliboramphus_antiquus | -0.658 | Calhim 2006 | 2.314 | Calhim 2006 |  | M | Pitcher 2005 |
| Tachybaptus_novaehollandiae | -0.432 | Calhim 2006 | 2.286 | Calhim 2006 |  | M | Pitcher 2005 |
| Tachycineta_bicolor | -0.194 | Calhim 2006 | 1.303 | Calhim 2006 |  | PG | Pitcher 2005; Biagolini 2017 |
| Taeniopygia_guttata | -1.301 | Hayward 2011 | 1.079 | Hayward 2011 |  | M | Garamszegi 2005; Pitcher 2005; Biagolini 2017 |
| Tangara_chilensis | -0.155 | Calhim 2006 | 1.316 | Calhim 2006 |  | M | Pitcher 2005 |
| Tangara_lavinia | -0.620 | Calhim 2006 | 1.342 | Calhim 2006 |  | M | Pitcher 2005 |
| Tersina_viridis | -0.444 | Calhim 2006 | 1.456 | Calhim 2006 |  | M | Pitcher 2005 |
| Lyrurus_tetrix | 0.491 | Hayward 2011 | 3.079 | Hayward 2011 | Lekking | PG | Beehler 1988 |
| Tetrao_urogallus | 0.477 | Hayward 2011 | 3.580 | Hayward 2011 | Lekking | PA/PGA | Pitcher 2005; Beletsky 1995 |
| Thamnophilus_punctatus | -1.523 | Calhim 2006 | 1.348 | Calhim 2006 |  | M | Pitcher 2005 |
| Thinocorus_orbignyianus | -0.886 | Calhim 2006 | 2.068 | Calhim 2006 |  | M | Pitcher 2005 |
| Thinornis_rubricollis | -1.699 | Calhim 2006 | 1.970 | Calhim 2006 |  | M | Pitcher 2005 |
| Thraupis_bonariensis | -0.125 | Calhim 2006 | 1.505 | Calhim 2006 |  | M | Pitcher 2005 |
| Thraupis_cyanocephala | -0.770 | Calhim 2006 | 1.544 | Calhim 2006 |  | M | Pitcher 2005 |
| Thraupis_episcopus | -0.495 | Hayward 2011 | 1.544 | Hayward 2011 |  | M | Garamszegi 2005; Pitcher 2005 |
| Thraupis_sayaca | -0.167 | Calhim 2006 | 1.505 | Calhim 2006 |  | M | Pitcher 2005 |
| Thryothorus_ludovicianus | -0.699 | Calhim 2006 | 1.308 | Calhim 2006 |  | M | Pitcher 2005; Biagolini 2017 |
| Tiaris_olivaceus | -0.886 | Calhim 2006 | 0.954 | Calhim 2006 |  | M | Pitcher 2005 |
| Timeliopsis_fulvigula | -0.721 | Calhim 2006 | 1.204 | Calhim 2006 |  | M | Pitcher 2005 |
| Tinamus_major | 0.822 | Calhim 2006 | 3.025 | Calhim 2006 |  | PA/PGA | Pitcher 2005 |
| Toxorhamphus_novaeguineae | -1.000 | Calhim 2006 | 1.176 | Calhim 2006 |  | M | Pitcher 2005 |
| Toxorhamphus_poliopterus | -1.046 | Calhim 2006 | 1.041 | Calhim 2006 |  | M | Pitcher 2005 |
| Toxostoma_curvirostre | 0.057 | Hayward 2011 | 1.900 | Hayward 2011 |  | M | Garamszegi 2005; Pitcher 2005 |
| Toxostoma_guttatum | -0.886 | Calhim 2006 | 1.723 | Calhim 2006 |  | M | Pitcher 2005 |
| Toxostoma_rufum | -0.180 | Calhim 2006 | 1.838 | Calhim 2006 |  | M | Pitcher 2005 |
| Tregellasia_leucops | -0.143 | Calhim 2006 | 1.146 | Calhim 2006 |  | C | Pitcher 2005 |
| Trichodere_cockerelli | -1.046 | Calhim 2006 | 1.182 | Calhim 2006 |  | M | Pitcher 2005 |
| Trichoglossus_chlorolepidotus | -1.046 | Calhim 2006 | 1.949 | Calhim 2006 |  | M | Pitcher 2005 |
| Trichoglossus_haematodus | -0.638 | Calhim 2006 | 2.090 | Calhim 2006 |  | M | Pitcher 2005 |
| Tringa_flavipes | -0.602 | Calhim 2006 | 1.908 | Calhim 2006 |  | M | Pitcher 2005 |
| Tringa_glareola | -0.252 | Calhim 2006 | 1.606 | Calhim 2006 |  | M | Pitcher 2005 |
| Tringa_melanoleuca | -0.585 | Calhim 2006 | 2.233 | Calhim 2006 |  | M | Pitcher 2005 |
| Tringa_solitaria | -1.000 | Calhim 2006 | 1.685 | Calhim 2006 |  | M | Pitcher 2005 |
| Troglodytes_aedon | -1.071 | Calhim 2006 | 1.045 | Calhim 2006 |  | PG | Pitcher 2005; Biagolini 2017 |
| Troglodytes_troglodytes | -0.921 | Calhim 2006 | 0.954 | Calhim 2006 |  | PG | Pitcher 2005 |
| Trogon_melanurus | -0.824 | Calhim 2006 | 2.063 | Calhim 2006 |  | M | Pitcher 2005 |
| Tryngites_subruficollis | -0.234 | Calhim 2006 | 1.826 | Calhim 2006 | Lekking | PG | Pitcher 2005; Cartar 1988; Szekely 2000 |
| Turdus_grayi | -0.108 | Hayward 2011 | 1.868 | Hayward 2011 |  | M | Garamszegi 2005; Pitcher 2005; Biagolini 2017 |
| Turdus_merula | 0.093 | Hayward 2011 | 2.025 | Hayward 2011 |  | M | Garamszegi 2005; Pitcher 2005 |
| Turdus_migratorius | -0.108 | Calhim 2006 | 1.873 | Calhim 2006 |  | M | Pitcher 2005; Biagolini 2017 |
| Turdus_philomelos | 0.167 | Calhim 2006 | 1.863 | Calhim 2006 |  | M | Pitcher 2005 |
| Turdus_pilaris | 0.479 | Calhim 2006 | 1.996 | Calhim 2006 |  | M | Pitcher 2005 |
| Turnix_pyrrhothorax | -0.585 | Calhim 2006 | 1.568 | Calhim 2006 |  | PA/PGA | Pitcher 2005 |
| Turnix_suscitator | -0.456 | Calhim 2006 | 2.000 | Calhim 2006 |  | PA/PGA | Pitcher 2005 |
| Turnix_sylvatica | -0.469 | Calhim 2006 | 1.653 | Calhim 2006 |  | PA/PGA | Pitcher 2005 |
| Tympanuchus_cupido | 0.207 | Calhim 2006 | 2.917 | Calhim 2006 | Lekking |  | Pitcher 2005 |
| Tympanuchus_phasianellus | 0.310 | Calhim 2006 | 2.887 | Calhim 2006 | Lekking |  | Pitcher 2005 |
| Tyrannus_crassirostris | -0.076 | Calhim 2006 | 1.748 | Calhim 2006 |  | M | Pitcher 2005 |
| Tyrannus_dominicensis | -0.357 | Calhim 2006 | 1.641 | Calhim 2006 |  | M | Pitcher 2005 |
| Tyrannus_forficatus | -0.155 | Calhim 2006 | 1.617 | Calhim 2006 |  | M | Pitcher 2005; Biagolini 2017 |
| Tyrannus_melancholicus | -0.292 | Calhim 2006 | 1.600 | Calhim 2006 |  | M | Pitcher 2005 |
| Tyrannus_savana | -0.398 | Calhim 2006 | 1.481 | Calhim 2006 |  | M | Pitcher 2005 |
| Tyrannus_tyrannus | -0.420 | Calhim 2006 | 1.611 | Calhim 2006 |  | M | Pitcher 2005; Biagolini 2017 |
| Tyrannus_vociferans | -0.071 | Calhim 2006 | 1.659 | Calhim 2006 |  | M | Pitcher 2005 |
| Tyto_alba | -0.523 | Calhim 2006 | 2.533 | Calhim 2006 |  | M | Pitcher 2005; Biagolini 2017 |
| Uria_aalge | 0.929 | Calhim 2006 | 2.961 | Calhim 2006 |  | M | Pitcher 2005; Biagolini 2017; Szekely 2000 |
| Uria_lomvia | 0.903 | Calhim 2006 | 2.978 | Calhim 2006 |  | M | Pitcher 2005; Biagolini 2017 |
| Uropsalis_segmentata | 0.061 | Calhim 2006 | 1.604 | Calhim 2006 |  | M | Pitcher 2005 |
| Oreothlypis_celata | -0.745 | Calhim 2006 | 0.954 | Calhim 2006 |  | M | Pitcher 2005 |
| Vermivora_chrysoptera | -0.921 | Calhim 2006 | 0.939 | Calhim 2006 |  | M | Pitcher 2005 |
| Oreothlypis_luciae | -0.921 | Calhim 2006 | 0.819 | Calhim 2006 |  | M | Pitcher 2005 |
| Oreothlypis_peregrina | -1.000 | Calhim 2006 | 1.009 | Calhim 2006 |  | M | Pitcher 2005 |
| Oreothlypis_ruficapilla | -0.921 | Calhim 2006 | 0.949 | Calhim 2006 |  | M | Pitcher 2005 |
| Oreothlypis_virginiae | -0.301 | Calhim 2006 | 0.940 | Calhim 2006 |  | M | Pitcher 2005 |
| Vidua_paradisaea | -1.046 | Calhim 2006 | 1.279 | Calhim 2006 | Lekking |  | Pitcher 2005 |
| Vireo_altiloquus | -0.602 | Calhim 2006 | 1.253 | Calhim 2006 |  | M | Pitcher 2005 |
| Vireo_atricapilla | -0.959 | Calhim 2006 | 0.929 | Calhim 2006 |  | M | Pitcher 2005 |
| Vireo_bellii | -0.959 | Calhim 2006 | 0.929 | Calhim 2006 |  | M | Pitcher 2005 |
| Vireo_flavifrons | -0.854 | Calhim 2006 | 1.255 | Calhim 2006 |  | M | Pitcher 2005 |
| Vireo_gilvus | -1.046 | Calhim 2006 | 1.121 | Calhim 2006 |  | M | Pitcher 2005 |
| Vireo_griseus | -0.796 | Calhim 2006 | 1.053 | Calhim 2006 |  | M | Pitcher 2005; Biagolini 2017 |
| Vireo_huttoni | -1.301 | Calhim 2006 | 1.064 | Calhim 2006 |  | M | Pitcher 2005 |
| Vireo_latimeri | -2.000 | Calhim 2006 | 1.049 | Calhim 2006 |  | M | Pitcher 2005 |
| Vireo_olivaceus | -0.387 | Calhim 2006 | 1.233 | Calhim 2006 |  | M | Pitcher 2005; Biagolini 2017 |
| Vireo_philadelphicus | -0.886 | Calhim 2006 | 1.086 | Calhim 2006 |  | M | Pitcher 2005 |
| Vireo_solitarius | -1.046 | Calhim 2006 | 1.220 | Calhim 2006 |  | M | Pitcher 2005; Biagolini 2017 |
| Vireo_vicinior | -1.222 | Calhim 2006 | 1.107 | Calhim 2006 |  | M | Pitcher 2005 |
| Setophaga_citrina | -0.367 | Calhim 2006 | 1.025 | Calhim 2006 |  | M | Pitcher 2005; Biagolini 2017 |
| Cardellina_pusilla | -0.796 | Calhim 2006 | 0.903 | Calhim 2006 |  | PG | Pitcher 2005 |
| Xanthocephalus_xanthocephalus | -0.013 | Hayward 2011 | 1.901 | Hayward 2011 |  | PG | Pitcher 2005 |
| Xanthotis_flaviventer | -1.398 | Calhim 2006 | 1.518 | Calhim 2006 |  | M | Pitcher 2005 |
| Zenaida_asiatica | -0.523 | Calhim 2006 | 2.185 | Calhim 2006 |  | M | Pitcher 2005 |
| Zenaida_aurita | -0.276 | Calhim 2006 | 2.201 | Calhim 2006 |  | M | Pitcher 2005 |
| Zenaida_macroura | -0.081 | Calhim 2006 | 2.093 | Calhim 2006 |  | M | Pitcher 2005 |
| Zonotrichia_albicollis | -0.367 | Hayward 2011 | 1.431 | Hayward 2011 |  | M | Garamszegi 2005; Pitcher 2005 |
| Zonotrichia_atricapilla | -0.268 | Calhim 2006 | 1.517 | Calhim 2006 |  | M | Pitcher 2005 |
| Zonotrichia_capensis | -0.699 | Calhim 2006 | 1.301 | Calhim 2006 |  | M | Garamszegi 2005; Pitcher 2005; Biagolini 2017 |
| Zonotrichia_leucophrys | -0.377 | Hayward 2011 | 1.468 | Hayward 2011 |  | PG | Pitcher 2005; Biagolini 2017 |
| Zonotrichia_querula | -0.032 | Calhim 2006 | 1.589 | Calhim 2006 |  | M | Pitcher 2005 |
| Zoothera_dauma | -1.000 | Calhim 2006 | 2.049 | Calhim 2006 |  | M | Pitcher 2005 |
| Zosterops_lateralis | -0.678 | Calhim 2006 | 1.079 | Calhim 2006 |  | M | Pitcher 2005; Biagolini 2017 |
| **Mammals** | | | | | | | |
| Acerodon_jubatus | 0.512 | Pitnick 2006 | 2.714 | Pitnick 2006 | Original data is for Acerodon_mackloti - taxa included by genus matching. |  |  |
| Axis_axis | 1.129 | Anderson 2004 | 4.735 | Anderson 2004 | Original data is for Axis_calamianensis - taxa included by genus matching. | PA/PGA | Anderson 2004 |
| Bunomys_andrewsi | -0.390 | Breed 2000 | 2.137 | Breed 2000 | Original data is for Bunomys_fratrorum - taxa included by genus matching. |  |  |
| Epomophorus_crypturus | -0.046 | Pitnick 2006 | 1.903 | Pitnick 2006 | Original data is for Epomophorus_labiatus - taxa included by genus matching. |  |  |
| Epomops_franqueti | -0.524 | Pitnick 2006 | 2.204 | Pitnick 2006 | Original data is for Epomops_buettikoferi - taxa included by genus matching. | PG | Hosken 2001 |
| Saccolaimus_flaviventris | -0.240 | Pitnick 2006 | 2.000 | Pitnick 2006 | Original data is for Saccolaimus_peli - taxa included by genus matching. | M | Hosken 2001; Pitnick 2006 |
| Abeomelomys_sevia | -0.069 | Breed 2000 | 1.716 | Breed 2000 |  |  |  |
| Acinonyx_jubatus | 1.041 | Iossa 2008 | 4.640 | Iossa 2008 |  | PA/PGA | Iossa 2008; Soulsbury 2010 |
| Acomys_cahirinus | -0.291 | Ramm 2005 | 1.878 | Ramm 2005 |  |  |  |
| Acrobates_pygmaeus | -0.750 | Birkhead 1998 | 1.090 | Birkhead 1998 |  | PA/PGA | Pantheria |
| Addax_nasomaculatus | 1.635 | Anderson 2004 | 4.929 | Anderson 2004 |  | PA/PGA | Anderson 2004 |
| Aepyceros_melampus | 1.461 | Anderson 2004 | 4.760 | Anderson 2004 |  | PA/PGA | Anderson 2004; Ginsberg 1990 |
| Aepyprymnus_rufescens | 0.670 | Birkhead 1998 | 3.380 | Birkhead 1998 |  |  |  |
| Aethomys_chrysophilus | -0.064 | Breed 2000 | 1.929 | Breed 2000 |  | M | Lukas 2012; Lukas 2013 |
| Aethomys_ineptus | -0.143 | Breed 2000 | 2.146 | Breed 2000 |  |  |  |
| Ailuropoda_melanoleuca | 2.487 | Iossa 2008 | 5.071 | Iossa 2008 |  | PA/PGA | Iossa 2008 |
| Akodon_molinae | -0.351 | Ramm 2005 | 1.618 | Ramm 2005 |  |  |  |
| Alcelaphus_buselaphus | 2.029 | Anderson 2004 | 5.204 | Anderson 2004 |  | PA/PGA | Anderson 2004; Ginsberg 1990 |
| Alces_alces | 2.025 | Ginsberg 1990 | 5.897 | Ginsberg 1990 |  | PG | Ginsberg 1990; Pantheria; Lukas 2012; Lukas 2013 |
| Allenopithecus_nigroviridis | 1.229 | Harcourt 1995 | 3.699 | Harcourt 1995 |  |  |  |
| Alouatta_palliata | 1.430 | Hayward 2011 | 3.864 | Hayward 2011 |  | PA/PGA | Kenagy 1986; Harcourt 1995 |
| Ammospermophilus_leucurus | 0.330 | Hayward 2011 | 1.994 | Hayward 2011 |  | PG | Waterman 2007 |
| Ammotragus_lervia | 1.275 | Anderson 2004 | 4.833 | Anderson 2004 |  | PA/PGA | Anderson 2004 |
| Anisomys_imitator | 0.620 | Ramm 2007 | 2.706 | Ramm 2007 |  |  |  |
| Anoura_cultrata | -1.004 | Pitnick 2006 | 1.233 | Pitnick 2006 |  |  |  |
| Anoura_geoffroyi | -1.301 | Pitnick 2006 | 1.179 | Pitnick 2006 |  |  |  |
| Antechinus_flavipes | -0.785 | Birkhead 1998 | 1.697 | Birkhead 1998 |  | PA/PGA | Rose 1997; Pantheria |
| Antechinus_stuartii | -0.456 | Hayward 2011 | 1.591 | Hayward 2011 |  | PA/PGA | Pantheria; Rose 1997 |
| Antechinus_swainsonii | -0.441 | Birkhead 1998 | 2.020 | Birkhead 1998 |  | PA/PGA | Pantheria; Rose 1997 |
| Antidorcas_marsupialis | 1.851 | Anderson 2004 | 4.607 | Anderson 2004 |  | PA/PGA | Anderson 2004; Ginsberg 1990 |
| Antilocapra_americana | 1.881 | Ginsberg 1990 | 4.790 | Ginsberg 1990 |  | PA/PGA | Soulsbury 2010; Ginsberg 1990; Lukas 2013; Lukas 2012 |
| Antrozous_pallidus | -0.770 | Ramm 2007 | 1.346 | Ramm 2007 |  |  |  |
| Aotus_trivirgatus | 0.079 | Hayward 2011 | 3.009 | Hayward 2011 |  | M | Pantheria; Kenagy 1986; Lukas 2013; Lukas 2012 |
| Apodemus_agrarius | -0.003 | Hayward 2011 | 1.338 | Hayward 2011 |  | PA/PGA | Soulsbury 2010 |
| Apodemus_argenteus | -0.207 | Hayward 2011 | 1.322 | Hayward 2011 |  |  |  |
| Apodemus_flavicollis | -0.050 | Hayward 2011 | 1.464 | Hayward 2011 |  | PA/PGA | Soulsbury 2010 |
| Apodemus_semotus | 0.139 | Breed 2000 | 1.447 | Breed 2000 |  |  |  |
| Apodemus_speciosus | 0.091 | Breed 2000 | 1.663 | Breed 2000 |  |  |  |
| Apodemus_sylvaticus | -0.103 | Hayward 2011 | 1.364 | Hayward 2011 |  | PA/PGA | Soulsbury 2010; Waterman 2007 |
| Apodemus_uralensis | -0.487 | Hayward 2011 | 1.283 | Hayward 2011 |  | PA/PGA | Soulsbury 2010 |
| Arctocephalus_forsteri | 1.462 | Fitzpatrick 2012 | 4.999 | Fitzpatrick 2012 |  | PG | Pantheria |
| Arctocephalus_pusillus | 1.645 | Fitzpatrick 2012 | 5.097 | Fitzpatrick 2012 |  | PG | Pantheria |
| Arctocephalus_tropicalis | 1.462 | Fitzpatrick 2012 | 4.985 | Fitzpatrick 2012 |  | PG | Pantheria |
| Ardops_nichollsi | -1.004 | Pitnick 2006 | 1.228 | Pitnick 2006 |  |  |  |
| Artibeus_jamaicensis | -1.292 | Pitnick 2006 | 1.602 | Pitnick 2006 |  | PG | Hosken 2001; Soulsbury 2010 |
| Artibeus_lituratus | -0.640 | Pitnick 2006 | 1.898 | Pitnick 2006 |  |  |  |
| Artibeus_phaeotis | -1.292 | Pitnick 2006 | 1.079 | Pitnick 2006 |  |  |  |
| Arvicola_amphibius | 0.326 | Hayward 2011 | 2.432 | Hayward 2011 |  |  |  |
| Atelerix_albiventris | 0.369 | Anderson 2004 | 2.716 | Anderson 2004 |  | PA/PGA | Anderson 2004 |
| Ateles_fusciceps | 0.959 | Moller 1988 | 3.687 | Moller 1988 |  | PA/PGA | Moller 1988 |
| Ateles_geoffroyi | 1.127 | Hayward 2011 | 3.900 | Hayward 2011 |  | PA/PGA | Pantheria; Kenagy 1986; Moller 1988; Harcourt 1995 |
| Avahi_laniger | 0.320 | Harcourt 1995 | 3.114 | Harcourt 1995 |  | M | Pantheria; Lukas 2013; Lukas 2012; Harcourt 1995 |
| Axis_porcinus | 1.694 | Anderson 2004 | 4.712 | Anderson 2004 |  | PG | Anderson 2004; Pantheria; Lukas 2012; Lukas 2013 |
| Balaena_mysticetus | 5.212 | Macleod 2010 | 7.708 | Macleod 2010 |  |  |  |
| Balaenoptera_acutorostrata | 3.944 | Dines 2015 | 7.079 | Dines 2015 |  | PA/PGA | Skaugh 2008 |
| Balaenoptera_borealis | 4.215 | Macleod 2010 | 7.255 | Macleod 2010 |  |  |  |
| Balaenoptera_edeni | 4.301 | Macleod 2010 | 7.255 | Macleod 2010 |  |  |  |
| Balaenoptera_musculus | 4.845 | Macleod 2010 | 8.029 | Macleod 2010 |  |  |  |
| Balaenoptera_physalus | 3.217 | Hayward 2011 | 7.581 | Hayward 2011 |  |  |  |
| Bandicota_bengalensis | 0.327 | Breed 2000 | 2.461 | Breed 2000 |  |  |  |
| Bandicota_indica | 0.309 | Hayward 2011 | 2.787 | Hayward 2011 |  |  |  |
| Bandicota_savilei | 0.101 | Breed 2000 | 2.438 | Breed 2000 |  |  |  |
| Bathyergus_suillus | 0.343 | Ramm 2005 | 2.875 | Ramm 2005 |  |  |  |
| Batomys_salomonseni | 0.192 | Breed 2000 | 2.288 | Breed 2000 |  |  |  |
| Berardius_bairdii | 4.000 | Macleod 2010 | 7.056 | Macleod 2010 |  |  |  |
| Berylmys_bowersi | 1.159 | Breed 2000 | 2.634 | Breed 2000 |  |  |  |
| Bettongia_gaimardi | 0.759 | Rose 1997 | 3.226 | Rose 1997 |  |  |  |
| Bettongia_penicillata | 0.222 | Birkhead 1998 | 2.941 | Birkhead 1998 |  |  |  |
| Bison_bonasus | 2.707 | Ginsberg 1990 | 5.829 | Ginsberg 1990 |  | PA/PGA | Ginsberg 1990 |
| Bos_taurus | 2.297 | Anderson 2004 | 5.954 | Anderson 2004 |  | PG | Anderson 2004; Lazo 1995; Bailey 2003 |
| Brachyphylla_cavernarum | -1.194 | Pitnick 2006 | 1.603 | Pitnick 2006 |  |  |  |
| Budorcas_taxicolor | 1.970 | Anderson 2004 | 5.398 | Anderson 2004 |  | PA/PGA | Anderson 2004 |
| Bullimus_bagobus | 0.611 | Breed 2000 | 2.580 | Breed 2000 |  |  |  |
| Bunomys_chrysocomus | 0.547 | Breed 2000 | 2.068 | Breed 2000 |  |  |  |
| Burramys_parvus | -1.429 | Birkhead 1998 | 1.591 | Birkhead 1998 |  |  |  |
| Cacajao_calvus | 0.763 | Harcourt 1995 | 3.538 | Harcourt 1995 |  | PA/PGA | Harcourt 1995 |
| Callithrix_argentata | 0.262 | Harcourt 1995 | 2.556 | Harcourt 1995 |  | M | Lukas 2012; Lukas 2013 |
| Callithrix_geoffroyi | -0.260 | Anderson 2004 | 2.580 | Anderson 2004 |  | M | Lukas 2013; Lukas 2012 |
| Callithrix_jacchus | 0.114 | Hayward 2011 | 2.505 | Hayward 2011 |  | M | Kenagy 1986; Lukas 2013; Lukas 2012 |
| Callithrix_pygmaea | -0.481 | Harcourt 1995 | 2.114 | Harcourt 1995 |  | M | Pantheria; Lukas 2012; Lukas 2013 |
| Callorhinus_ursinus | 1.803 | Hayward 2011 | 4.462 | Hayward 2011 |  | PG | Ferguson 2004 |
| Callospermophilus_lateralis | 0.512 | Hayward 2011 | 2.273 | Hayward 2011 |  | PA/PGA | Kenagy 1986; Waterman 2007 |
| Callospermophilus_saturatus | 0.362 | Hayward 2011 | 2.356 | Hayward 2011 |  |  |  |
| Calomys_musculinus | -0.704 | Ramm 2005 | 1.549 | Ramm 2005 |  |  |  |
| Camelus_dromedarius | 2.085 | Anderson 2004 | 5.903 | Anderson 2004 |  | PG | Anderson 2004; Nowak 1983 |
| Canis_aureus | 1.124 | Iossa 2008 | 4.000 | Iossa 2008 |  | M | Iossa 2008; Pantheria; Lukas 2013; Lukas 2012; Ferguson 2004 |
| Canis_latrans | 1.188 | Soulsbury 2010 | 4.025 | Soulsbury 2010 |  | M | Iossa 2008; Soulsbury 2010; Pantheria; Lukas 2013; Lukas 2012; Ferguson 2004 |
| Canis_lupus | 1.437 | Iossa 2008 | 4.516 | Iossa 2008 |  | M | Iossa 2008; Pantheria; Lukas 2013; Lukas 2012; Ferguson 2004 |
| Caperea_marginata | 3.279 | Macleod 2010 | 6.462 | Macleod 2010 |  |  |  |
| Capra_falconeri | 1.321 | Anderson 2004 | 4.580 | Anderson 2004 |  | PA/PGA | Anderson 2004 |
| Capra_ibex | 1.584 | Anderson 2004 | 4.875 | Anderson 2004 |  | PA/PGA | Anderson 2004 |
| Capreolus_capreolus | 1.653 | Hayward 2011 | 4.310 | Hayward 2011 |  | PG | Soulsbury 2010; Kenagy 1986; Pantheria |
| Capricornis_crispus | 1.806 | Ginsberg 1990 | 5.079 | Ginsberg 1990 |  | PA/PGA | Ginsberg 1990; Lukas 2013 |
| Cardioderma_cor | -1.398 | Ramm 2007 | 1.462 | Ramm 2007 |  | M | Hosken 2001; Pitnick 2006; Pantheria; Lukas 2013 |
| Carollia_perspicillata | -0.903 | Pitnick 2006 | 1.267 | Pitnick 2006 |  | PG | Hosken 2001; Pitnick 2006; Pantheria |
| Castor_canadensis | 0.968 | Hayward 2011 | 4.280 | Hayward 2011 |  | M | Soulsbury 2010; Pantheria; Kenagy 1986; Lukas 2013; Lukas 2012 |
| Castor_fiber | 1.241 | Hayward 2011 | 4.260 | Hayward 2011 |  | M | Lukas 2013; Lukas 2012; Waterman 2007 |
| Cavia_aperea | 0.572 | Soulsbury 2010 | 2.754 | Soulsbury 2010 |  | PG | Soulsbury 2010; Waterman 2007 |
| Cavia_porcellus | 0.613 | Hayward 2011 | 2.910 | Hayward 2011 |  |  |  |
| Cebus_apella | 0.959 | Ramm 2007 | 3.415 | Ramm 2007 |  | PA/PGA | Moller 1988; Harcourt 1995 |
| Centurio_senex | -1.097 | Pitnick 2006 | 1.360 | Pitnick 2006 |  |  |  |
| Cephalophus_silvicultor | 1.522 | Anderson 2004 | 4.884 | Anderson 2004 |  | PG | Anderson 2004; Pantheria; Lukas 2012; Lukas 2013 |
| Cephalorhynchus_commersonii | 3.086 | Macleod 2010 | 4.892 | Macleod 2010 |  |  |  |
| Cephalorhynchus_heavisidii | 2.728 | Dines 2015 | 4.872 | Dines 2015 |  |  |  |
| Cephalorhynchus_hectori | 2.948 | Macleod 2010 | 4.614 | Macleod 2010 |  |  |  |
| Cercartetus_concinnus | -1.208 | Birkhead 1998 | 1.155 | Birkhead 1998 |  |  |  |
| Cercartetus_nanus | -1.174 | Birkhead 1998 | 1.290 | Birkhead 1998 |  |  |  |
| Cercocebus_atys | 1.400 | Harcourt 1995 | 3.939 | Harcourt 1995 |  | PA/PGA | Lukas 2013; Harcourt 1995 |
| Cercocebus_torquatus | 1.400 | Hayward 2011 | 3.939 | Hayward 2011 |  |  |  |
| Cercopithecus_ascanius | 0.477 | Harcourt 1995 | 3.729 | Harcourt 1995 |  | PA/PGA | Harcourt 1995 |
| Cervus_elaphus | 1.848 | Anderson 2004 | 5.505 | Anderson 2004 |  | PA/PGA | Soulsbury 2010; Kenagy 1986 |
| Chaerephon_pumilus | -1.420 | Pitnick 2006 | 1.033 | Pitnick 2006 |  | PG | Hosken 2001; Pitnick 2006 |
| Chaetodipus_formosus | -0.678 | Hayward 2011 | 1.294 | Hayward 2011 |  | PA/PGA | Waterman 2007 |
| Chalinolobus_gouldii | -2.000 | Ramm 2007 | 1.130 | Ramm 2007 |  |  |  |
| Cheirogaleus_major | 0.362 | Harcourt 1995 | 2.531 | Harcourt 1995 |  | PG | Pantheria; Lukas 2013; Lukas 2012 |
| Chinchilla_lanigera | 0.716 | Hayward 2011 | 2.626 | Hayward 2011 |  |  |  |
| Chiropodomys_gliroides | -0.420 | Breed 2000 | 1.342 | Breed 2000 |  |  |  |
| Chiruromys_vates | 0.242 | Breed 2000 | 1.681 | Breed 2000 |  |  |  |
| Chlorocebus_aethiops | 1.314 | Hayward 2011 | 3.723 | Hayward 2011 |  | PA/PGA | Kenagy 1986; Moller 1988; Harcourt 1995 |
| Chrotopterus_auritus | -0.740 | Pitnick 2006 | 1.964 | Pitnick 2006 |  | M | Hosken 2001; Pitnick 2006; Lukas 2013 |
| Chrysocyon_brachyurus | 1.103 | Iossa 2008 | 4.498 | Iossa 2008 |  | M | Iossa 2008; Pantheria; Lukas 2013 |
| Coendou_prehensilis | 0.389 | Anderson 2004 | 3.481 | Anderson 2004 |  | PG | Anderson 2004; Lukas 2012; Lukas 2013 |
| Coleura_afra | -1.523 | Pitnick 2006 | 1.000 | Pitnick 2006 |  | PG | Hosken 2001; Pitnick 2006 |
| Colobus_guereza | 0.474 | Harcourt 1995 | 4.017 | Harcourt 1995 |  | PA/PGA | Harcourt 1995 |
| Colobus_polykomos | 1.137 | Hayward 2011 | 3.993 | Hayward 2011 |  | PA/PGA | Kenagy 1986; Harcourt 1995 |
| Conilurus_penicillatus | 0.642 | Ramm 2007 | 2.265 | Ramm 2007 |  |  |  |
| Connochaetes_gnou | 2.137 | Ginsberg 1990 | 5.134 | Ginsberg 1990 |  | PA/PGA | Ginsberg 1990 |
| Connochaetes_taurinus | 2.486 | Ginsberg 1990 | 5.356 | Ginsberg 1990 |  | PA/PGA | Ginsberg 1990 |
| Crocidura_russula | -1.398 | Soulsbury 2010 | 1.152 | Soulsbury 2010 |  | M | Soulsbury 2010 |
| Crocidura_suaveolens | -1.000 | Hayward 2011 | 0.875 | Hayward 2011 |  |  |  |
| Crocuta_crocuta | 0.993 | Soulsbury 2010 | 4.653 | Soulsbury 2010 |  | PA/PGA | Iossa 2008; Soulsbury 2010 |
| Cryptomys_hottentotus | -0.237 | Soulsbury 2010 | 2.017 | Soulsbury 2010 |  | M | Soulsbury 2010; Pantheria; Lukas 2013; Lukas 2012 |
| Cryptoprocta_ferox | 1.047 | Anderson 2004 | 4.029 | Anderson 2004 |  | PA/PGA | Ferguson 2004; Iossa 2008 |
| Ctenomys_talarum | -0.500 | Hayward 2011 | 2.127 | Hayward 2011 |  | PG | Soulsbury 2010; Waterman 2007 |
| Cuon_alpinus | 0.803 | Anderson 2004 | 4.252 | Anderson 2004 |  | M | Lukas 2013; Lukas 2012 |
| Cynictis_penicillata | 0.297 | Soulsbury 2010 | 2.954 | Soulsbury 2010 |  | PG | Iossa 2008; Soulsbury 2010; Lukas 2013; Lukas 2012 |
| Cynomops_abrasus | -0.733 | Pitnick 2006 | 0.903 | Pitnick 2006 |  |  |  |
| Cynomys_ludovicianus | 0.602 | Soulsbury 2010 | 3.021 | Soulsbury 2010 |  | PG | Soulsbury 2010; Waterman 2007 |
| Cynopterus_brachyotis | -0.921 | Ramm 2007 | 1.498 | Ramm 2007 |  |  |  |
| Cynopterus_sphinx | -0.745 | Ramm 2007 | 1.570 | Ramm 2007 |  | PA/PGA | Hosken 2001; Pitnick 2006 |
| Cystophora_cristata | 2.034 | Fitzpatrick 2012 | 5.409 | Fitzpatrick 2012 |  | M | Lukas 2012; Ferguson 2004 |
| Dactylopsila_trivirgata | -0.602 | Birkhead 1998 | 2.669 | Birkhead 1998 |  |  |  |
| Dama_dama | 1.602 | Anderson 2004 | 4.775 | Anderson 2004 |  | PA/PGA | Anderson 2004; Ginsberg 1990 |
| Damaliscus_pygargus | 2.204 | Ginsberg 1990 | 4.860 | Ginsberg 1990 |  | PA/PGA | Ginsberg 1990 |
| Dasykaluta_rosamondae | -0.900 | Birkhead 1998 | 1.548 | Birkhead 1998 |  |  |  |
| Dasymys_incomtus | 0.484 | Hayward 2011 | 2.384 | Hayward 2011 |  |  |  |
| Dasyuroides_byrnei | 0.140 | Birkhead 1998 | 2.092 | Birkhead 1998 |  |  |  |
| Dasyurus_hallucatus | 0.130 | Birkhead 1998 | 2.973 | Birkhead 1998 |  |  |  |
| Dasyurus_viverrinus | 0.372 | Birkhead 1998 | 3.183 | Birkhead 1998 |  |  |  |
| Delphinapterus_leucas | 3.221 | Dines 2015 | 6.098 | Dines 2015 |  |  |  |
| Delphinus_capensis | 3.807 | Dines 2015 | 5.104 | Dines 2015 |  |  |  |
| Delphinus_delphis | 3.207 | Hayward 2011 | 4.924 | Hayward 2011 |  |  |  |
| Desmodus_rotundus | -1.081 | Pitnick 2006 | 1.528 | Pitnick 2006 |  | PG | Hosken 2001; Pitnick 2006 |
| Dicrostonyx_groenlandicus | -0.860 | Hayward 2011 | 1.792 | Hayward 2011 |  |  |  |
| Didelphis_albiventris | 0.246 | Birkhead 1998 | 3.111 | Birkhead 1998 |  |  |  |
| Didelphis_virginiana | 0.477 | Anderson 2004 | 3.574 | Anderson 2004 |  | PG | Anderson 2004; Lukas 2012; Lukas 2013 |
| Dipodomys_merriami | -0.347 | Hayward 2011 | 1.587 | Hayward 2011 |  | PA/PGA | Waterman 2007 |
| Dipodomys_microps | -0.398 | Hayward 2011 | 1.789 | Hayward 2011 |  |  |  |
| Dipodomys_ordii | -0.276 | Hayward 2011 | 1.751 | Hayward 2011 |  |  |  |
| Dipodomys_panamintinus | -0.284 | Hayward 2011 | 1.883 | Hayward 2011 |  |  |  |
| Ectophylla_alba | -1.102 | Pitnick 2006 | 0.740 | Pitnick 2006 |  | PG | Hosken 2001 |
| Eidolon_helvum | 0.452 | Ramm 2007 | 2.413 | Ramm 2007 |  | PA/PGA | Hosken 2001 |
| Elaphurus_davidianus | 1.759 | Anderson 2004 | 5.330 | Anderson 2004 |  | PA/PGA | Anderson 2004 |
| Elephas_maximus | 3.458 | Anderson 2004 | 6.658 | Anderson 2004 |  | PA/PGA | Anderson 2004; Ginsberg 1990; Rasmussen 1998 |
| Eonycteris_spelaea | -0.398 | Ramm 2007 | 1.792 | Ramm 2007 |  | PG | Hosken 2001; Lukas 2013 |
| Eptesicus_furinalis | -1.222 | Ramm 2007 | 0.877 | Ramm 2007 |  |  |  |
| Equus_asinus | 2.306 | Anderson 2004 | 5.462 | Anderson 2004 |  | PA/PGA | Anderson 2004 |
| Equus_burchellii | 2.480 | Ginsberg 1990 | 5.377 | Ginsberg 1990 |  | PG | Pantheria; Lukas 2012; Lukas 2013 |
| Equus_caballus | 2.531 | Anderson 2004 | 5.398 | Anderson 2004 |  | PA/PGA | Linklater 1999; Anderson 2004; Ginsberg 1990 |
| Equus_grevyi | 3.061 | Ginsberg 1990 | 5.587 | Ginsberg 1990 |  | PA/PGA | Ginsberg 1990 |
| Equus_kiang | 2.210 | Anderson 2004 | 5.544 | Anderson 2004 |  | PG | Anderson 2004; Lukas 2012; Lukas 2013 |
| Equus_zebra | 2.146 | Anderson 2004 | 5.536 | Anderson 2004 |  | PG | Anderson 2004; Lukas 2013; Lukas 2012; Ginsberg 1990 |
| Erignathus_barbatus | 2.109 | Fitzpatrick 2012 | 5.431 | Fitzpatrick 2012 |  | PA/PGA | Ferguson 2004 |
| Erinaceus_europaeus | 0.494 | Anderson 2004 | 2.845 | Anderson 2004 |  | PA/PGA | Anderson 2004; Soulsbury 2010 |
| Erythrocebus_patas | 0.857 | Ramm 2007 | 4.000 | Ramm 2007 |  | PA/PGA | Moller 1988; Harcourt 1995 |
| Eschrichtius_robustus | 4.829 | Macleod 2010 | 7.398 | Macleod 2010 |  |  |  |
| Eubalaena_japonica | 5.988 | Dines 2015 | 7.895 | Dines 2015 |  |  |  |
| Euderma_maculatum | -1.180 | Pitnick 2006 | 1.176 | Pitnick 2006 |  |  |  |
| Eudorcas_rufifrons | 0.898 | Anderson 2004 | 4.428 | Anderson 2004 |  | PA/PGA | Anderson 2004 |
| Eulemur_fulvus | 0.891 | Soulsbury 2010 | 3.398 | Soulsbury 2010 |  | PG | Soulsbury 2010; Ostner 1999 |
| Eulemur_macaco | 1.221 | Harcourt 1995 | 3.398 | Harcourt 1995 |  | PA/PGA | Harcourt 1995 |
| Eumetopias_jubatus | 2.250 | Ramm 2007 | 5.986 | Ramm 2007 |  | PG | Ferguson 2004 |
| Felis_catus | 0.558 | Soulsbury 2010 | 3.679 | Soulsbury 2010 |  | PG | Iossa 2008; Soulsbury 2010 |
| Felis_chaus | 0.665 | Iossa 2008 | 3.907 | Iossa 2008 |  |  |  |
| Felis_nigripes | 0.325 | Iossa 2008 | 3.297 | Iossa 2008 |  | PG | Iossa 2008 |
| Felis_silvestris | 0.438 | Anderson 2004 | 3.438 | Anderson 2004 |  | PA/PGA | Anderson 2004; Iossa 2008; Ferguson 2004 |
| Feresa_attenuata | 2.877 | Dines 2015 | 5.356 | Dines 2015 |  |  |  |
| Funambulus_palmarum | 0.354 | Hayward 2011 | 2.157 | Hayward 2011 |  |  |  |
| Galago_alleni | 0.210 | Anderson 2004 | 2.497 | Anderson 2004 |  | PA/PGA | Anderson 2004 |
| Galago_senegalensis | 0.220 | Anderson 2004 | 2.322 | Anderson 2004 |  | PA/PGA | Anderson 2004 |
| Galagoides_demidoff | -0.009 | Anderson 2004 | 1.908 | Anderson 2004 |  | PA/PGA | Anderson 2004 |
| Galidia_elegans | 0.229 | Iossa 2008 | 2.997 | Iossa 2008 |  | M | Iossa 2008; Pantheria; Lukas 2013; Lukas 2012 |
| Gazella_cuvieri | 1.595 | Anderson 2004 | 4.423 | Anderson 2004 |  | PG | Anderson 2004; Alados 1994 |
| Gazella_dorcas | 1.498 | Anderson 2004 | 4.165 | Anderson 2004 |  | PG | Anderson 2004; Lukas 2012; Lukas 2013; Pantheria |
| Gazella_leptoceros | 0.823 | Anderson 2004 | 4.061 | Anderson 2004 |  | PA/PGA | Anderson 2004 |
| Gazella_subgutturosa | 0.916 | Anderson 2004 | 4.398 | Anderson 2004 |  | PA/PGA | Anderson 2004 |
| Genetta_genetta | -0.018 | Iossa 2008 | 3.276 | Iossa 2008 |  | PA/PGA | Iossa 2008; Ferguson 2004; Lukas 2013; Lukas 2012 |
| Gerbilliscus_afra | 0.889 | Hayward 2011 | 1.964 | Hayward 2011 |  |  |  |
| Gerbilliscus_brantsii | 0.725 | Hayward 2011 | 1.973 | Hayward 2011 |  |  |  |
| Giraffa_camelopardalis | 2.362 | Anderson 2004 | 5.953 | Anderson 2004 |  | PA/PGA | Anderson 2004; Ginsberg 1990 |
| Globicephala_macrorhynchus | 4.158 | Dines 2015 | 6.556 | Dines 2015 |  |  |  |
| Globicephala_melas | 3.699 | Hayward 2011 | 6.289 | Hayward 2011 |  |  |  |
| Glossophaga_leachii | -1.538 | Pitnick 2006 | 0.924 | Pitnick 2006 |  |  |  |
| Glossophaga_morenoi | -2.000 | Hosken 2001 | 0.924 | Hosken 2001 |  |  |  |
| Gorilla_beringei | 1.556 | Soulsbury 2010 | 5.301 | Soulsbury 2010 |  | PA/PGA | Soulsbury 2010; Lukas 2013 |
| Gorilla_gorilla | 1.365 | Hayward 2011 | 5.127 | Hayward 2011 |  | PG | Pantheria; Kenagy 1986; Moller 1988; Harcourt 1995 |
| Grammomys_macmillani | -0.179 | Breed 2000 | 1.602 | Breed 2000 |  |  |  |
| Grampus_griseus | 4.104 | Dines 2015 | 5.699 | Dines 2015 |  |  |  |
| Gulo_gulo | 1.230 | Soulsbury 2010 | 4.161 | Soulsbury 2010 |  | PA/PGA | Iossa 2008; Soulsbury 2010; Pantheria; Ferguson 2004 |
| Gymnobelideus_leadbeateri | -0.951 | Birkhead 1998 | 2.063 | Birkhead 1998 |  | PG | Lukas 2012; Lukas 2013; Rose 1997 |
| Halichoerus_grypus | 2.204 | Fitzpatrick 2012 | 5.399 | Fitzpatrick 2012 |  | PG | Lukas 2012; Ferguson 2004; Pantheria |
| Haplonycteris_fischeri | -0.092 | Pitnick 2006 | 1.255 | Pitnick 2006 |  |  |  |
| Helogale_parvula | -0.347 | Anderson 2004 | 2.658 | Anderson 2004 |  | M | Lukas 2013; Lukas 2012; Pantheria; Allaine 2000 |
| Hemitragus_jemlahicus | 1.427 | Anderson 2004 | 4.881 | Anderson 2004 |  | PA/PGA | Anderson 2004 |
| Herpailurus_yaguarondi | 0.519 | Iossa 2008 | 3.771 | Iossa 2008 |  | PG | Iossa 2008 |
| Herpestes_javanicus | 0.593 | Iossa 2008 | 2.841 | Iossa 2008 |  | PA/PGA | Iossa 2008 |
| Heterohyrax_brucei | 1.114 | Hayward 2011 | 3.176 | Hayward 2011 |  | PG | Pantheria |
| Hippopotamus_amphibius | 2.813 | Ginsberg 1990 | 6.204 | Ginsberg 1990 |  | PG | Pantheria; Lukas 2012; Lukas 2013 |
| Hipposideros_galeritus | -0.523 | Ramm 2007 | 0.813 | Ramm 2007 |  | M | Hosken 2001; Pitnick 2006; Lukas 2013 |
| Hipposideros_speoris | -1.398 | Ramm 2007 | 1.000 | Ramm 2007 |  |  |  |
| Homo_sapiens | 1.701 | Anderson 2004 | 4.803 | Anderson 2004 |  | PG | Anderson 2004; Kenagy 1986; Moller 1988; Harcourt 1995 |
| Hyaena_hyaena | 0.891 | Iossa 2008 | 4.525 | Iossa 2008 |  | PA/PGA | Iossa 2008; Soulsbury 2010 |
| Hydrochoerus_hydrochaeris | 1.611 | Anderson 2004 | 4.770 | Anderson 2004 |  | PA/PGA | Anderson 2004 |
| Hydromys_chrysogaster | 1.071 | Ramm 2007 | 2.872 | Ramm 2007 |  |  |  |
| Hylobates_agilis | 0.801 | Harcourt 1995 | 3.778 | Harcourt 1995 |  | M | Lukas 2013; Lukas 2012; Harcourt 1995 |
| Hylobates_lar | 0.740 | Hayward 2011 | 3.740 | Hayward 2011 |  | M | Pantheria; Kenagy 1986; Lukas 2013; Lukas 2012; Moller 1988; Harcourt 1995 |
| Hylobates_moloch | 0.756 | Hayward 2011 | 3.749 | Hayward 2011 |  | M | Kenagy 1986; Lukas 2013; Lukas 2012; Moller 1988; Harcourt 1995 |
| Hylochoerus_meinertzhageni | 2.699 | Ginsberg 1990 | 5.305 | Ginsberg 1990 |  | PA/PGA | Ginsberg 1990; Lukas 2013 |
| Hyomys_goliath | 0.574 | Hayward 2011 | 3.000 | Hayward 2011 |  |  |  |
| Hyperoodon_ampullatus | 3.078 | Macleod 2010 | 6.845 | Macleod 2010 |  |  |  |
| Hypsignathus_monstrosus | -0.620 | Ramm 2007 | 2.549 | Ramm 2007 |  | PG | Hosken 2001 |
| Hystrix_africaeaustralis | 0.823 | Ramm 2005 | 4.072 | Ramm 2005 |  | M | Lukas 2013; Lukas 2012; Waterman 2007 |
| Inia_geoffrensis | 3.160 | Hayward 2011 | 5.086 | Hayward 2011 |  |  |  |
| Isoodon_macrourus | 0.373 | Hayward 2011 | 3.106 | Hayward 2011 |  | PG | Nowak 1983 |
| Isoodon_obesulus | 0.230 | Hayward 2011 | 3.024 | Hayward 2011 |  | PG | Rose 1997; Lukas 2013 |
| Kobus_ellipsiprymnus | 1.867 | Anderson 2004 | 5.306 | Anderson 2004 |  | PA/PGA | Anderson 2004; Ginsberg 1990 |
| Kobus_kob | 1.620 | Anderson 2004 | 5.164 | Anderson 2004 |  | PA/PGA | Anderson 2004; Ginsberg 1990 |
| Kobus_leche | 1.832 | Anderson 2004 | 5.037 | Anderson 2004 |  | PA/PGA | Anderson 2004 |
| Kobus_megaceros | 1.716 | Anderson 2004 | 5.015 | Anderson 2004 |  | PA/PGA | Anderson 2004 |
| Kogia_breviceps | 4.021 | Dines 2015 | 5.657 | Dines 2015 |  |  |  |
| Kogia_sima | 3.692 | Dines 2015 | 5.481 | Dines 2015 |  |  |  |
| Lagenorhynchus_acutus | 2.521 | Macleod 2010 | 5.301 | Macleod 2010 |  |  |  |
| Lagenorhynchus_obliquidens | 3.048 | Dines 2015 | 5.297 | Dines 2015 |  |  |  |
| Lagenorhynchus_obscurus | 3.427 | Macleod 2010 | 4.903 | Macleod 2010 |  |  |  |
| Lagothrix_lagotricha | 1.049 | Hayward 2011 | 3.718 | Hayward 2011 |  | PA/PGA | Pantheria; Kenagy 1986; Lukas 2013; Lukas 2012; Harcourt 1995 |
| Lasiorhinus_latifrons | 1.208 | Anderson 2004 | 4.407 | Anderson 2004 |  | PA/PGA | Anderson 2004 |
| Lasiurus_ega | -1.699 | Ramm 2007 | 1.077 | Ramm 2007 |  |  |  |
| Lasiurus_seminolus | -1.886 | Pitnick 2006 | 0.968 | Pitnick 2006 |  |  |  |
| Leggadina_forresti | -0.264 | Ramm 2007 | 1.477 | Ramm 2007 |  |  |  |
| Leontopithecus_rosalia | -0.319 | Anderson 2004 | 2.716 | Anderson 2004 |  | M | Lukas 2013; Lukas 2012; Harcourt 1995 |
| Leopardus_colocolo | 0.489 | Iossa 2008 | 3.525 | Iossa 2008 |  | PG | Iossa 2008 |
| Leopardus_geoffroyi | 0.782 | Iossa 2008 | 3.681 | Iossa 2008 |  | PG | Iossa 2008 |
| Leopardus_pardalis | 1.547 | Iossa 2008 | 4.155 | Iossa 2008 |  | PA/PGA | Iossa 2008; Ferguson 2004 |
| Leopardus_tigrinus | 0.665 | Iossa 2008 | 3.477 | Iossa 2008 |  | PG | Iossa 2008; Lukas 2013 |
| Leopardus_wiedii | 0.834 | Iossa 2008 | 3.531 | Iossa 2008 |  | PG | Iossa 2008 |
| Leopoldamys_sabanus | 0.956 | Breed 2000 | 2.572 | Breed 2000 |  |  |  |
| Leporillus_conditor | 0.602 | Ramm 2007 | 2.272 | Ramm 2007 |  |  |  |
| Leptomys_elegans | 0.093 | Ramm 2007 | 1.839 | Ramm 2007 |  |  |  |
| Leptonycteris_curasoae | -0.580 | Pitnick 2006 | 1.462 | Pitnick 2006 |  |  |  |
| Lepus_americanus | 1.072 | Soulsbury 2010 | 3.114 | Soulsbury 2010 |  | PA/PGA | Soulsbury 2010 |
| Lepus_timidus | 1.222 | Hayward 2011 | 3.421 | Hayward 2011 |  |  |  |
| Lipotes_vexillifer | 1.778 | Dines 2015 | 5.097 | Dines 2015 |  |  |  |
| Lissodelphis_borealis | 3.189 | Dines 2015 | 5.055 | Dines 2015 |  |  |  |
| Lontra_canadensis | 1.255 | Iossa 2008 | 3.792 | Iossa 2008 |  | PA/PGA | Iossa 2008; Ferguson 2004 |
| Lophocebus_aterrimus | 1.139 | Harcourt 1995 | 4.000 | Harcourt 1995 |  | PA/PGA | Lukas 2013; Harcourt 1995 |
| Lophostoma_carrikeri | -0.388 | Pitnick 2006 | 1.362 | Pitnick 2006 |  |  |  |
| Lophuromys_flavopunctatus | 0.056 | Breed 2000 | 1.778 | Breed 2000 |  |  |  |
| Lorentzimys_nouhuysi | -0.447 | Hayward 2011 | 1.204 | Hayward 2011 |  |  |  |
| Loris_tardigradus | 0.310 | Hayward 2011 | 2.442 | Hayward 2011 |  | PA/PGA | Gupta 2007 (and therein) |
| Loxodonta_africana | 3.486 | Anderson 2004 | 6.702 | Anderson 2004 |  | PA/PGA | Anderson 2004; Ginsberg 1990; Rasmussen 1998 |
| Lutra_lutra | 0.607 | Anderson 2004 | 3.893 | Anderson 2004 |  | PG | Anderson 2004; Iossa 2008 |
| Lycalopex_culpaeus | 1.274 | Iossa 2008 | 3.957 | Iossa 2008 |  | M | Iossa 2008 |
| Lycalopex_griseus | 1.049 | Iossa 2008 | 3.398 | Iossa 2008 |  | M | Iossa 2008 |
| Lycaon_pictus | 1.583 | Iossa 2008 | 4.498 | Iossa 2008 |  | PA/PGA | Iossa 2008; Soulsbury 2010; Pantheria; Lukas 2013; Lukas 2012 |
| Lynx_lynx | 0.857 | Iossa 2008 | 4.292 | Iossa 2008 |  | PA/PGA | Iossa 2008 |
| Lynx_rufus | 0.706 | Iossa 2008 | 4.079 | Iossa 2008 |  | PA/PGA | Iossa 2008; Ferguson 2004 |
| Macaca_arctoides | 1.683 | Hayward 2011 | 4.022 | Hayward 2011 |  | PA/PGA | Kenagy 1986; Moller 1988 |
| Macaca_fascicularis | 1.547 | Anderson 2004 | 3.653 | Anderson 2004 |  | PA/PGA | Soulsbury 2010; Kenagy 1986; Moller 1988 |
| Macaca_fuscata | 1.845 | Soulsbury 2010 | 4.090 | Soulsbury 2010 |  | PA/PGA | Soulsbury 2010; Moller 1988 |
| Macaca_mulatta | 1.690 | Anderson 2004 | 4.049 | Anderson 2004 |  | PA/PGA | Kenagy 1986; Moller 1988; Harcourt 1995 |
| Macaca_nemestrina | 1.824 | Hayward 2011 | 3.999 | Hayward 2011 |  | PA/PGA | Kenagy 1986; Moller 1988 |
| Macaca_radiata | 1.760 | Hayward 2011 | 3.924 | Hayward 2011 |  | PA/PGA | Kenagy 1986; Moller 1988 |
| Macroderma_gigas | -0.775 | Pitnick 2006 | 2.033 | Pitnick 2006 |  |  |  |
| Macroglossus_minimus | -0.854 | Ramm 2007 | 1.180 | Ramm 2007 |  |  |  |
| Macroglossus_sobrinus | -0.796 | Ramm 2007 | 1.362 | Ramm 2007 |  |  |  |
| Macropus_agilis | 1.409 | Birkhead 1998 | 4.057 | Birkhead 1998 |  | PA/PGA | Rose 1997; Pantheria |
| Macropus_eugenii | 1.792 | Birkhead 1998 | 3.767 | Birkhead 1998 |  | PA/PGA | Rose 1997; Pantheria |
| Macropus_fuliginosus | 1.713 | Birkhead 1998 | 4.533 | Birkhead 1998 |  | PA/PGA | Rose 1997; Pantheria |
| Macropus_giganteus | 1.623 | Birkhead 1998 | 4.610 | Birkhead 1998 |  | PA/PGA | Pantheria; Rose 1997 |
| Macropus_robustus | 1.602 | Rose 1997 | 4.477 | Rose 1997 |  | PA/PGA | Rose 1997; Pantheria |
| Macropus_rufogriseus | 1.737 | Birkhead 1998 | 4.267 | Birkhead 1998 |  | PA/PGA | Rose 1997; Pantheria |
| Macropus_rufus | 1.582 | Birkhead 1998 | 4.600 | Birkhead 1998 |  | PA/PGA | Pantheria |
| Macrotus_waterhousii | -1.432 | Pitnick 2006 | 1.204 | Pitnick 2006 |  |  |  |
| Madoqua_guentheri | 0.498 | Anderson 2004 | 3.512 | Anderson 2004 |  | M | Anderson 2004; Lukas 2013 |
| Madoqua_kirkii | 0.924 | Hayward 2011 | 3.699 | Hayward 2011 |  | M | Soulsbury 2010; Pantheria; Kenagy 1986; Lukas 2013; Lukas 2012 |
| Mallomys_rothschildi | 1.157 | Hayward 2011 | 3.036 | Hayward 2011 |  |  |  |
| Mammelomys_lanosus | 0.478 | Breed 2000 | 2.090 | Breed 2000 |  |  |  |
| Mandrillus_leucophaeus | 1.436 | Anderson 2004 | 4.455 | Anderson 2004 |  | PA/PGA | Anderson 2004 |
| Mandrillus_sphinx | 1.833 | Harcourt 1995 | 4.544 | Harcourt 1995 |  | PG | Harcourt 1995 |
| Marmota_monax | 0.869 | Hayward 2011 | 3.620 | Hayward 2011 |  | PA/PGA | Kenagy 1986; Waterman 2007 |
| Martes_pennanti | 0.916 | Hayward 2011 | 3.724 | Hayward 2011 |  | PA/PGA | Iossa 2008; Ferguson 2004 |
| Mastacomys_fuscus | 0.571 | Ramm 2007 | 2.068 | Ramm 2007 |  |  |  |
| Mastomys_coucha | -0.130 | Breed 2000 | 1.845 | Breed 2000 |  |  |  |
| Mastomys_natalensis | 0.204 | Hayward 2011 | 1.910 | Hayward 2011 |  | PA/PGA | Soulsbury 2010 |
| Maxomys_bartelsii | 0.624 | Breed 2000 | 1.968 | Breed 2000 |  |  |  |
| Maxomys_surifer | 0.522 | Breed 2000 | 2.190 | Breed 2000 |  |  |  |
| Maxomys_whiteheadi | -0.021 | Breed 2000 | 1.806 | Breed 2000 |  |  |  |
| Megaderma_lyra | -1.097 | Ramm 2007 | 1.806 | Ramm 2007 |  |  |  |
| Megaderma_spasma | -0.947 | Pitnick 2006 | 1.354 | Pitnick 2006 |  |  |  |
| Megaptera_novaeangliae | 3.091 | Hayward 2011 | 7.502 | Hayward 2011 |  | PA/PGA | Pantheria |
| Meles_meles | 1.158 | Hayward 2011 | 4.162 | Hayward 2011 |  | PA/PGA | Iossa 2008; Soulsbury 2010; Kenagy 1986 |
| Mellivora_capensis | 1.732 | Iossa 2008 | 3.987 | Iossa 2008 |  | PA/PGA | Iossa 2008; Ferguson 2004 |
| Melomys_burtoni | 0.281 | Breed 2000 | 1.892 | Breed 2000 |  |  |  |
| Melomys_cervinipes | 0.444 | Breed 2000 | 2.143 | Breed 2000 |  |  |  |
| Melomys_leucogaster | 0.042 | Breed 2000 | 2.017 | Breed 2000 |  |  |  |
| Melomys_rufescens | 0.128 | Ramm 2007 | 1.833 | Ramm 2007 |  |  |  |
| Mephitis_mephitis | 0.703 | Iossa 2008 | 3.703 | Iossa 2008 |  | PG | Iossa 2008; Ferguson 2004 |
| Mesembriomys_gouldii | 0.740 | Ramm 2007 | 2.736 | Ramm 2007 |  |  |  |
| Mesembriomys_macrurus | 0.918 | Breed 2000 | 2.533 | Breed 2000 |  |  |  |
| Mesocricetus_auratus | 0.501 | Hayward 2011 | 2.033 | Hayward 2011 |  |  |  |
| Mesoplodon_carlhubbsi | 2.699 | Dines 2015 | 6.176 | Dines 2015 |  |  |  |
| Mesoplodon_europaeus | 2.505 | Dines 2015 | 6.079 | Dines 2015 |  |  |  |
| Mesoplodon_ginkgodens | 2.447 | Dines 2015 | 6.079 | Dines 2015 |  |  |  |
| Mesoplodon_mirus | 2.515 | Dines 2015 | 6.009 | Dines 2015 |  |  |  |
| Mesoplodon_perrini | 2.301 | Dines 2015 | 6.079 | Dines 2015 |  |  |  |
| Micaelamys_namaquensis | 0.169 | Breed 2000 | 1.857 | Breed 2000 |  |  |  |
| Microcebus_murinus | 0.262 | Soulsbury 2010 | 1.855 | Soulsbury 2010 |  | PA/PGA | Soulsbury 2010 |
| Micromys_minutus | -0.750 | Hayward 2011 | 0.892 | Hayward 2011 |  |  |  |
| Micropteropus_pusillus | -0.921 | Ramm 2007 | 1.511 | Ramm 2007 |  | PG | Hosken 2001 |
| Microtus_agrestis | -0.095 | Hayward 2011 | 1.667 | Hayward 2011 |  |  |  |
| Microtus_arvalis | -0.373 | Hayward 2011 | 1.455 | Hayward 2011 |  |  |  |
| Microtus_californicus | -0.569 | Hayward 2011 | 1.818 | Hayward 2011 |  | PG | Lukas 2012; Waterman 2007; Pantheria |
| Microtus_montanus | -0.420 | Ramm 2007 | 1.633 | Ramm 2007 |  | PA/PGA | Lukas 2012; Waterman 2007; Pantheria |
| Microtus_ochrogaster | -0.056 | Soulsbury 2010 | 1.580 | Soulsbury 2010 |  | M | Soulsbury 2010; Pantheria; Lukas 2013; Lukas 2012 |
| Microtus_oeconomus | -0.378 | Hayward 2011 | 1.542 | Hayward 2011 |  | PA/PGA | Soulsbury 2010; Waterman 2007 |
| Microtus_pennsylvanicus | 0.068 | Soulsbury 2010 | 1.737 | Soulsbury 2010 |  | PA/PGA | Soulsbury 2010; Waterman 2007 |
| Microtus_pinetorum | -1.155 | Ramm 2007 | 1.303 | Ramm 2007 |  | PA/PGA | Waterman 2007; Lukas 2012; Lukas 2013 |
| Microtus_subterraneus | -0.625 | Hayward 2011 | 1.233 | Hayward 2011 |  |  |  |
| Millardia_meltada | 0.033 | Breed 2000 | 1.857 | Breed 2000 |  |  |  |
| Miniopterus_australis | -1.398 | Pitnick 2006 | 0.875 | Pitnick 2006 |  | PG | Hosken 2001; Pitnick 2006 |
| Miniopterus_minor | -1.301 | Pitnick 2006 | 0.875 | Pitnick 2006 |  | PA/PGA | Hosken 2001; Pitnick 2006 |
| Miniopterus_schreibersii | -1.097 | Pitnick 2006 | 1.114 | Pitnick 2006 |  |  |  |
| Miopithecus_talapoin | 0.716 | Harcourt 1995 | 3.097 | Harcourt 1995 |  | PA/PGA | Harcourt 1995 |
| Monodelphis_domestica | -0.244 | Birkhead 1998 | 2.041 | Birkhead 1998 |  |  |  |
| Monodon_monoceros | 3.082 | Dines 2015 | 6.329 | Dines 2015 |  |  |  |
| Monophyllus_plethodon | -1.377 | Pitnick 2006 | 1.190 | Pitnick 2006 |  |  |  |
| Mops_condylurus | -0.921 | Pitnick 2006 | 1.602 | Pitnick 2006 |  |  |  |
| Mormopterus_planiceps | -1.046 | Ramm 2007 | 0.978 | Ramm 2007 |  |  |  |
| Mungos_mungo | 0.246 | Anderson 2004 | 2.176 | Anderson 2004 |  | PA/PGA | Anderson 2004; Ferguson 2004 |
| Muntiacus_muntjak | 0.813 | Anderson 2004 | 4.243 | Anderson 2004 |  | PG | Anderson 2004; Lukas 2012; Lukas 2013 |
| Mus_caroli | -0.967 | Hayward 2011 | 1.176 | Hayward 2011 |  |  |  |
| Mus_minutoides | -1.167 | Hayward 2011 | 0.903 | Hayward 2011 |  | M | Lukas 2012 |
| Mus_musculus | -0.924 | Hayward 2011 | 1.185 | Hayward 2011 |  | PA/PGA | Soulsbury 2010; Kenagy 1986 |
| Mustela_erminea | 0.422 | Hayward 2011 | 2.511 | Hayward 2011 |  | PA/PGA | Iossa 2008; Soulsbury 2010; Pantheria; Ferguson 2004 |
| Mustela_eversmannii | 0.485 | Iossa 2008 | 3.312 | Iossa 2008 |  | PA/PGA | Iossa 2008 |
| Mustela_frenata | 0.274 | Iossa 2008 | 2.458 | Iossa 2008 |  | PA/PGA | Iossa 2008; Ferguson 2004 |
| Mustela_nigripes | 0.473 | Iossa 2008 | 3.009 | Iossa 2008 |  | PA/PGA | Iossa 2008; Ferguson 2004 |
| Mustela_nivalis | -0.377 | Hayward 2011 | 2.064 | Hayward 2011 |  | PA/PGA | Iossa 2008; Ferguson 2004 |
| Mustela_putorius | 0.516 | Hayward 2011 | 3.020 | Hayward 2011 |  | PA/PGA | Iossa 2008; Ferguson 2004 |
| Mustela_sibirica | 0.220 | Iossa 2008 | 2.620 | Iossa 2008 |  | PG | Iossa 2008 |
| Myodes_glareolus | -0.190 | Hayward 2011 | 1.338 | Hayward 2011 |  | PA/PGA | Soulsbury 2010; Waterman 2007 |
| Myodes_rufocanus | -0.793 | Soulsbury 2010 | 1.591 | Soulsbury 2010 |  | PA/PGA | Soulsbury 2010; Lukas 2013 |
| Myotis_adversus | -0.845 | Pitnick 2006 | 1.000 | Pitnick 2006 |  | PA/PGA | Hosken 2001; Pitnick 2006 |
| Myotis_albescens | -0.434 | Pitnick 2006 | 0.740 | Pitnick 2006 |  | PA/PGA | Hosken 2001; Pitnick 2006 |
| Myotis_bocagii | -1.097 | Pitnick 2006 | 0.903 | Pitnick 2006 |  | PG | Hosken 2001; Pitnick 2006 |
| Myotis_evotis | -0.717 | Pitnick 2006 | 0.833 | Pitnick 2006 |  |  |  |
| Myotis_lucifugus | -1.301 | Ramm 2007 | 0.854 | Ramm 2007 |  | PA/PGA | Hosken 2001; Pitnick 2006 |
| Myotis_nigricans | -1.585 | Pitnick 2006 | 0.602 | Pitnick 2006 |  | PG | Hosken 2001; Pitnick 2006 |
| Naemorhedus_goral | 1.562 | Anderson 2004 | 4.439 | Anderson 2004 |  | PG | Anderson 2004; Lukas 2012; Lukas 2013; Pantheria |
| Nanger_dama | 1.197 | Anderson 2004 | 4.648 | Anderson 2004 |  |  |  |
| Nasalis_larvatus | 1.167 | Hayward 2011 | 4.336 | Hayward 2011 |  | PA/PGA | Kenagy 1986; Harcourt 1995 |
| Nasua_nasua | 0.714 | Iossa 2008 | 3.751 | Iossa 2008 |  | PA/PGA | Iossa 2008; Ferguson 2004 |
| Neofelis_nebulosa | 1.480 | Anderson 2004 | 4.398 | Anderson 2004 |  | PA/PGA | Ferguson 2004 |
| Neomys_fodiens | -0.678 | Hayward 2011 | 1.301 | Hayward 2011 |  |  |  |
| Neophocaena_phocaenoides | 3.248 | Macleod 2010 | 4.856 | Macleod 2010 |  |  |  |
| Neoromicia_nanus | -1.509 | Pitnick 2006 | 0.544 | Pitnick 2006 |  | PA/PGA | Hosken 2001; Pitnick 2006 |
| Neotoma_micropus | 0.398 | Soulsbury 2010 | 2.423 | Soulsbury 2010 |  | PA/PGA | Soulsbury 2010 |
| Neotragus_moschatus | 1.770 | Anderson 2004 | 3.813 | Anderson 2004 |  | PG | Anderson 2004; Lukas 2012; Lukas 2013 |
| Neovison_vison | 0.695 | Soulsbury 2010 | 3.233 | Soulsbury 2010 |  | PA/PGA | Iossa 2008; Soulsbury 2010; Ferguson 2004 |
| Niviventer_cremoriventer | 0.087 | Breed 2000 | 1.875 | Breed 2000 |  |  |  |
| Niviventer_culturatus | 0.273 | Breed 2000 | 2.013 | Breed 2000 |  |  |  |
| Noctilio_albiventris | -1.194 | Pitnick 2006 | 1.494 | Pitnick 2006 |  |  |  |
| Noctilio_leporinus | -0.752 | Pitnick 2006 | 1.812 | Pitnick 2006 |  | PG | Hosken 2001; Pitnick 2006 |
| Notomys_alexis | -1.301 | Hayward 2011 | 1.531 | Hayward 2011 |  | M | Lukas 2012 |
| Notomys_cervinus | -0.703 | Hayward 2011 | 1.519 | Hayward 2011 |  |  |  |
| Notomys_fuscus | -1.174 | Hayward 2011 | 1.643 | Hayward 2011 |  |  |  |
| Notomys_mitchellii | -1.208 | Hayward 2011 | 1.591 | Hayward 2011 |  |  |  |
| Nyctalus_noctula | -0.569 | Ramm 2007 | 1.455 | Ramm 2007 |  | PA/PGA | Hosken 2001; Pitnick 2006 |
| Nyctereutes_procyonoides | 0.963 | Iossa 2008 | 3.540 | Iossa 2008 |  | M | Iossa 2008; Pantheria; Lukas 2013; Lukas 2012; Ferguson 2004 |
| Nycticebus_coucang | 0.079 | Anderson 2004 | 3.024 | Anderson 2004 |  | PA/PGA | Anderson 2004 |
| Nyctophilus_geoffroyi | -1.523 | Ramm 2007 | 0.806 | Ramm 2007 |  |  |  |
| Nyctophilus_gouldi | -0.959 | Ramm 2007 | 0.903 | Ramm 2007 |  |  |  |
| Ochotona_curzoniae | 0.210 | Soulsbury 2010 | 2.169 | Soulsbury 2010 |  | PG | Soulsbury 2010 |
| Odobenus_rosmarus | 2.696 | Fitzpatrick 2012 | 5.936 | Fitzpatrick 2012 |  | PG | Ferguson 2004 |
| Odocoileus_hemionus | 1.939 | Hayward 2011 | 5.050 | Hayward 2011 |  | M | Kenagy 1986 |
| Odocoileus_virginianus | 1.932 | Hayward 2011 | 4.651 | Hayward 2011 |  | PA/PGA | Soulsbury 2010; Kenagy 1986 |
| Okapia_johnstoni | 1.856 | Anderson 2004 | 5.398 | Anderson 2004 |  | PG | Anderson 2004; Lukas 2012; Lukas 2013 |
| Ondatra_zibethicus | 0.704 | Hayward 2011 | 3.112 | Hayward 2011 |  | M | Kenagy 1986; Lukas 2013; Lukas 2012 |
| Onychomys_torridus | 0.170 | Hayward 2011 | 1.307 | Hayward 2011 |  |  |  |
| Orcinus_orca | 4.013 | Macleod 2010 | 6.867 | Macleod 2010 |  | PA/PGA | Pilot 2009 |
| Ornithorhynchus_anatinus | 1.230 | Birkhead 1998 | 3.176 | Birkhead 1998 |  |  |  |
| Oryctolagus_cuniculus | 0.741 | Anderson 2004 | 3.279 | Anderson 2004 |  | PA/PGA | Anderson 2004; Soulsbury 2010 |
| Oryx_dammah | 1.728 | Anderson 2004 | 5.111 | Anderson 2004 |  | PA/PGA | Anderson 2004 |
| Oryx_gazella | 1.579 | Anderson 2004 | 5.152 | Anderson 2004 |  | PG | Anderson 2004; Lukas 2012; Lukas 2013; Pantheria |
| Oryzomys_palustris | -0.398 | Hayward 2011 | 1.748 | Hayward 2011 |  |  |  |
| Otaria_byronia | 1.842 | Fitzpatrick 2012 | 5.477 | Fitzpatrick 2012 |  | PG | Pantheria |
| Otocolobus_manul | 0.342 | Iossa 2008 | 3.738 | Iossa 2008 |  | PG | Iossa 2008 |
| Otomops_martiensseni | -0.770 | Pitnick 2006 | 1.556 | Pitnick 2006 |  | PG | Hosken 2001; Pitnick 2006 |
| Otospermophilus_beecheyi | 0.957 | Hayward 2011 | 2.924 | Hayward 2011 |  | PG | Soulsbury 2010; Waterman 2007 |
| Ovis_aries | 2.504 | Hayward 2011 | 4.544 | Hayward 2011 |  | PA/PGA | Soulsbury 2010; Kenagy 1986 |
| Ovis_canadensis | 2.529 | Anderson 2004 | 5.076 | Anderson 2004 |  | PA/PGA | Anderson 2004; Ginsberg 1990 |
| Ovis_nivicola | 1.909 | Anderson 2004 | 4.881 | Anderson 2004 |  | PA/PGA | Anderson 2004 |
| Pan_paniscus | 2.398 | Soulsbury 2010 | 4.653 | Soulsbury 2010 |  | PA/PGA | Soulsbury 2010; Moller 1988 |
| Pan_troglodytes | 2.075 | Hayward 2011 | 4.647 | Hayward 2011 |  | PA/PGA | Soulsbury 2010; Kenagy 1986; Moller 1988; Harcourt 1995; Pantheria |
| Panthera_leo | 1.740 | Soulsbury 2010 | 5.274 | Soulsbury 2010 |  | PA/PGA | Iossa 2008; Soulsbury 2010; Pantheria; Ferguson 2004 |
| Panthera_onca | 1.931 | Anderson 2004 | 4.892 | Anderson 2004 |  | PA/PGA | Ferguson 2004; Iossa 2008 |
| Panthera_tigris | 1.387 | Anderson 2004 | 5.532 | Anderson 2004 |  | PA/PGA | Ferguson 2004 |
| Papio_anubis | 1.971 | Ramm 2007 | 4.422 | Ramm 2007 |  |  |  |
| Papio_cynocephalus | 1.716 | Soulsbury 2010 | 5.386 | Soulsbury 2010 |  | PA/PGA | Soulsbury 2010; Moller 1988; Harcourt 1995 |
| Papio_hamadryas | 1.859 | Hayward 2011 | 4.384 | Hayward 2011 |  | PG | Kenagy 1986; Harcourt 1995 |
| Papio_papio | 1.949 | Harcourt 1995 | 4.505 | Harcourt 1995 |  |  |  |
| Papio_ursinus | 1.857 | Harcourt 1995 | 4.502 | Harcourt 1995 |  |  |  |
| Paramelomys_platyops | 0.223 | Ramm 2007 | 1.954 | Ramm 2007 |  |  |  |
| Paramelomys_rubex | 0.272 | Breed 2000 | 1.699 | Breed 2000 |  |  |  |
| Parastrellus_hesperus | -1.301 | Ramm 2007 | 0.544 | Ramm 2007 |  |  |  |
| Paruromys_dominator | 0.204 | Breed 2000 | 2.534 | Breed 2000 |  |  |  |
| Pecari_tajacu | 1.053 | Hayward 2011 | 4.090 | Hayward 2011 |  | PA/PGA | Ginsberg 1990 |
| Pedetes_capensis | 1.261 | Ramm 2005 | 3.509 | Ramm 2005 |  | M | Lukas 2012 |
| Peponocephala_electra | 3.254 | Macleod 2010 | 5.314 | Macleod 2010 |  |  |  |
| Perameles_nasuta | 0.602 | Rose 1997 | 3.000 | Rose 1997 |  | PA/PGA | Rose 1997; Lee 1985 |
| Perognathus_longimembris | -1.155 | Hayward 2011 | 0.892 | Hayward 2011 |  |  |  |
| Perognathus_parvus | -0.745 | Hayward 2011 | 1.274 | Hayward 2011 |  |  |  |
| Peromyscus_californicus | -2.018 | Soulsbury 2010 | 1.719 | Nelson 1995 |  | M | Soulsbury 2010; Pantheria; Lukas 2013; Lukas 2012 |
| Peromyscus_leucopus | -0.337 | Soulsbury 2010 | 1.346 | Soulsbury 2010 |  | PA/PGA | Soulsbury 2010; Waterman 2007 |
| Peromyscus_maniculatus | -0.406 | Hayward 2011 | 1.290 | Hayward 2011 |  | PA/PGA | Soulsbury 2010; Kenagy 1986; Waterman 2007 |
| Petauroides_volans | 0.199 | Hayward 2011 | 3.096 | Hayward 2011 |  | PG | Pantheria; Lukas 2012; Lukas 2013; Rose 1997 |
| Petaurus_breviceps | -0.699 | Birkhead 1998 | 2.076 | Birkhead 1998 |  | PG | Pantheria; Lukas 2012; Lukas 2013; Rose 1997 |
| Petaurus_norfolcensis | -0.790 | Birkhead 1998 | 2.255 | Birkhead 1998 |  |  |  |
| Phacochoerus_aethiopicus | 1.968 | Anderson 2004 | 4.863 | Anderson 2004 |  | PA/PGA | Anderson 2004; Ginsberg 1990; Somers 1995 |
| Phascolarctos_cinereus | 1.175 | Anderson 2004 | 3.674 | Anderson 2004 |  | PA/PGA | Anderson 2004; Pantheria |
| Philander_opossum | 0.176 | Rose 1997 | 2.581 | Rose 1997 |  |  |  |
| Phoca_groenlandica | 2.107 | Fitzpatrick 2012 | 5.011 | Fitzpatrick 2012 |  | PA/PGA | Ferguson 2004 |
| Phoca_vitulina | 1.978 | Fitzpatrick 2012 | 4.875 | Fitzpatrick 2012 |  | PA/PGA | Ferguson 2004 |
| Phocarctos_hookeri | 1.821 | Fitzpatrick 2012 | 5.350 | Fitzpatrick 2012 |  | PG | Pantheria |
| Phocoena_phocoena | 3.365 | Hayward 2011 | 4.763 | Hayward 2011 |  |  |  |
| Phocoena_sinus | 3.048 | Macleod 2010 | 4.589 | Macleod 2010 |  |  |  |
| Phocoena_spinipinnis | 2.692 | Macleod 2010 | 4.786 | Macleod 2010 |  |  |  |
| Phocoenoides_dalli | 2.582 | Macleod 2010 | 5.162 | Macleod 2010 |  |  |  |
| Phodopus_sungorus | -0.032 | Hayward 2011 | 1.627 | Hayward 2011 |  |  |  |
| Phyllostomus_discolor | -0.188 | Pitnick 2006 | 1.652 | Pitnick 2006 |  | PG | Hosken 2001; Pitnick 2006 |
| Phyllostomus_hastatus | -0.511 | Pitnick 2006 | 1.963 | Pitnick 2006 |  | PG | Hosken 2001; Pitnick 2006 |
| Physeter_catodon | 4.080 | Macleod 2010 | 7.569 | Macleod 2010 |  |  |  |
| Pipistrellus_kuhlii | -1.301 | Ramm 2007 | 0.765 | Ramm 2007 |  |  |  |
| Pipistrellus_pipistrellus | -0.959 | Ramm 2007 | 0.729 | Ramm 2007 |  | PA/PGA | Hosken 2001; Pitnick 2006 |
| Pipistrellus_rusticus | -0.796 | Ramm 2007 | 0.602 | Ramm 2007 |  |  |  |
| Pipistrellus_subflavus | -1.523 | Ramm 2007 | 0.747 | Ramm 2007 |  |  |  |
| Pipistrellus_tenuis | -1.523 | Ramm 2007 | 0.556 | Ramm 2007 |  |  |  |
| Pithecia_pithecia | -0.036 | Harcourt 1995 | 3.204 | Harcourt 1995 |  | PA/PGA | Pantheria; Moller 1988; Lukas 2013; Lukas 2012 |
| Platanista_gangetica | 3.045 | Dines 2015 | 4.959 | Dines 2015 |  |  |  |
| Plecotus_auritus | -1.046 | Ramm 2007 | 0.890 | Ramm 2007 |  | PA/PGA | Hosken 2001; Pitnick 2006 |
| Plecotus_rafinesquii | -0.420 | Ramm 2007 | 0.959 | Ramm 2007 |  | PA/PGA | Hosken 2001; Pitnick 2006 |
| Pogonomys_loriae | 0.540 | Breed 2000 | 2.021 | Breed 2000 |  |  |  |
| Pogonomys_macrourus | 0.391 | Breed 2000 | 1.690 | Breed 2000 |  |  |  |
| Pongo_pygmaeus | 1.548 | Anderson 2004 | 5.000 | Anderson 2004 |  | PA/PGA | Anderson 2004; Kenagy 1986; Moller 1988 |
| Pontoporia_blainvillei | 1.301 | Dines 2015 | 4.618 | Dines 2015 |  |  |  |
| Potorous_tridactylus | 0.641 | Birkhead 1998 | 3.107 | Birkhead 1998 |  | PA/PGA | Pantheria |
| Praomys_jacksoni | -0.052 | Breed 2000 | 1.602 | Breed 2000 |  |  |  |
| Presbytis_rubicunda | 0.556 | Hayward 2011 | 3.792 | Hayward 2011 |  | PG | Pantheria |
| Prionailurus_viverrinus | 1.196 | Anderson 2004 | 4.061 | Anderson 2004 |  | PA/PGA | Ferguson 2004; Iossa 2008 |
| Procavia_capensis | 1.236 | Anderson 2004 | 3.306 | Anderson 2004 |  | PG | Anderson 2004; Lukas 2012; Lukas 2013 |
| Procyon_lotor | 1.137 | Soulsbury 2010 | 3.710 | Soulsbury 2010 |  | PA/PGA | Iossa 2008; Soulsbury 2010; Pantheria; Ferguson 2004 |
| Proteles_cristatus | 1.003 | Iossa 2008 | 4.053 | Iossa 2008 |  | M | Iossa 2008; Lukas 2013; Lukas 2012 |
| Przewalskium_albirostris | 2.066 | Anderson 2004 | 5.334 | Anderson 2004 |  | PA/PGA | Anderson 2004 |
| Pseudantechinus_macdonnellensis | -0.959 | Birkhead 1998 | 1.433 | Birkhead 1998 |  |  |  |
| Pseudocheirus_peregrinus | 0.734 | Birkhead 1998 | 2.835 | Birkhead 1998 |  | PG | Pantheria; Lukas 2012; Lukas 2013; Rose 1997 |
| Pseudochirops_archeri | 0.097 | Birkhead 1998 | 2.679 | Birkhead 1998 |  |  |  |
| Pseudohydromys_ellermani | -0.268 | Breed 2000 | 1.255 | Breed 2000 |  |  |  |
| Pseudomys_apodemoides | -0.851 | Hayward 2011 | 1.491 | Hayward 2011 |  |  |  |
| Pseudomys_australis | 0.351 | Hayward 2011 | 1.771 | Hayward 2011 |  |  |  |
| Pseudomys_delicatulus | -1.367 | Hayward 2011 | 0.903 | Hayward 2011 |  |  |  |
| Pseudomys_desertor | -0.253 | Hayward 2011 | 1.591 | Hayward 2011 |  |  |  |
| Pseudomys_fumeus | 0.385 | Ramm 2007 | 1.851 | Ramm 2007 |  |  |  |
| Pseudomys_gracilicaudatus | 0.035 | Hayward 2011 | 2.021 | Hayward 2011 |  |  |  |
| Pseudomys_hermannsburgensis | -0.788 | Hayward 2011 | 1.255 | Hayward 2011 |  |  |  |
| Pseudomys_nanus | 0.273 | Hayward 2011 | 1.892 | Hayward 2011 |  |  |  |
| Pseudomys_novaehollandiae | -1.149 | Hayward 2011 | 1.279 | Hayward 2011 |  |  |  |
| Pseudomys_pilligaensis | -1.284 | Breed 2000 | 1.041 | Breed 2000 |  |  |  |
| Pseudomys_shortridgei | -0.462 | Hayward 2011 | 1.892 | Hayward 2011 |  |  |  |
| Pseudorca_crassidens | 4.170 | Dines 2015 | 6.134 | Dines 2015 |  |  |  |
| Pteronotus_davyi | -0.759 | Pitnick 2006 | 0.964 | Pitnick 2006 |  |  |  |
| Pteropus_alecto | 0.407 | Ramm 2007 | 2.903 | Ramm 2007 |  | PA/PGA | Hosken 2001; Pitnick 2006 |
| Pteropus_giganteus | 0.342 | Anderson 2004 | 3.161 | Anderson 2004 |  | PA/PGA | Anderson 2004; Hosken 2001 |
| Pteropus_poliocephalus | 0.829 | Ramm 2007 | 2.925 | Ramm 2007 |  | PA/PGA | Hosken 2001; Pitnick 2006 |
| Pteropus_scapulatus | 0.423 | Ramm 2007 | 2.690 | Ramm 2007 |  | PA/PGA | Hosken 2001; Pitnick 2006 |
| Pteropus_tonganus | 0.580 | Hayward 2011 | 3.011 | Hayward 2011 |  | PG | Hosken 2001 |
| Puma_concolor | 1.173 | Soulsbury 2010 | 4.653 | Soulsbury 2010 |  | PA/PGA | Iossa 2008; Soulsbury 2010; Pantheria; Ferguson 2004 |
| Pusa_hispida | 1.677 | Fitzpatrick 2012 | 4.839 | Fitzpatrick 2012 |  | PG | Smith 1981 |
| Pygathrix_nemaeus | 0.924 | Anderson 2004 | 4.269 | Anderson 2004 |  | PA/PGA | Anderson 2004 |
| Rangifer_tarandus | 1.678 | Anderson 2004 | 5.043 | Anderson 2004 |  | PA/PGA | Soulsbury 2010 |
| Rattus_argentiventer | 0.484 | Breed 2000 | 2.336 | Breed 2000 |  |  |  |
| Rattus_colletti | 0.287 | Ramm 2007 | 2.188 | Ramm 2007 |  |  |  |
| Rattus_exulans | 0.415 | Hayward 2011 | 1.778 | Hayward 2011 |  |  |  |
| Rattus_fuscipes | 0.630 | Ramm 2007 | 2.041 | Ramm 2007 |  | PA/PGA | Waterman 2007 |
| Rattus_hoffmanni | 0.373 | Breed 2000 | 2.146 | Breed 2000 |  |  |  |
| Rattus_leucopus | 0.618 | Breed 2000 | 2.230 | Breed 2000 |  |  |  |
| Rattus_losea | 0.337 | Breed 2000 | 2.041 | Breed 2000 |  |  |  |
| Rattus_lutreolus | 0.647 | Ramm 2007 | 2.212 | Ramm 2007 |  | PA/PGA | Waterman 2007 |
| Rattus_niobe | 0.277 | Ramm 2007 | 1.690 | Ramm 2007 |  |  |  |
| Rattus_norvegicus | 0.580 | Anderson 2004 | 2.748 | Anderson 2004 |  | PA/PGA | Anderson 2004 |
| Rattus_rattus | 0.857 | Hayward 2011 | 2.210 | Hayward 2011 |  |  |  |
| Rattus_sordidus | 0.547 | Breed 2000 | 2.199 | Breed 2000 |  |  |  |
| Rattus_steini | 0.045 | Ramm 2007 | 1.968 | Ramm 2007 |  |  |  |
| Rattus_tiomanicus | 0.541 | Breed 2000 | 1.949 | Breed 2000 |  |  |  |
| Rattus_tunneyi | 0.687 | Ramm 2007 | 2.386 | Ramm 2007 |  |  |  |
| Rattus_villosissimus | 0.332 | Ramm 2007 | 2.316 | Ramm 2007 |  |  |  |
| Rhabdomys_pumilio | -0.041 | Breed 2000 | 1.740 | Breed 2000 |  | M | Lukas 2012 |
| Rhinolophus_clivosus | -0.745 | Ramm 2007 | 1.149 | Ramm 2007 |  |  |  |
| Rhinolophus_hipposideros | -1.699 | Ramm 2007 | 0.640 | Ramm 2007 |  |  |  |
| Rhinolophus_megaphyllus | -0.921 | Ramm 2007 | 0.978 | Ramm 2007 |  |  |  |
| Rhinonicteris_aurantia | -1.921 | Pitnick 2006 | 0.991 | Pitnick 2006 |  |  |  |
| Rhinopoma_hardwickii | -0.886 | Ramm 2007 | 1.279 | Ramm 2007 |  |  |  |
| Rhinopoma_microphyllum | -0.201 | Ramm 2007 | 1.507 | Ramm 2007 |  |  |  |
| Rhogeessa_tumida | -1.114 | Pitnick 2006 | 0.708 | Pitnick 2006 |  |  |  |
| Rhynchonycteris_naso | -2.000 | Ramm 2007 | 0.627 | Ramm 2007 |  | PA/PGA | Hosken 2001; Pitnick 2006 |
| Rousettus_aegyptiacus | 0.243 | Ramm 2007 | 2.180 | Ramm 2007 |  |  |  |
| Rousettus_amplexicaudatus | -0.432 | Ramm 2007 | 1.921 | Ramm 2007 |  |  |  |
| Rusa_timorensis | 1.438 | Anderson 2004 | 4.875 | Anderson 2004 |  | PA/PGA | Anderson 2004 |
| Rusa_unicolor | 1.889 | Anderson 2004 | 5.303 | Anderson 2004 |  | PG | Anderson 2004; Lukas 2013 |
| Saccopteryx_bilineata | -1.959 | Pitnick 2006 | 0.869 | Pitnick 2006 |  | PG | Hosken 2001; Pitnick 2006; Soulsbury 2010; Pantheria |
| Saccopteryx_leptura | -1.602 | Pitnick 2006 | 0.613 | Pitnick 2006 |  | M | Pitnick 2006; Pantheria |
| Saguinus_midas | 0.262 | Harcourt 1995 | 2.756 | Harcourt 1995 |  | PA/PGA | Pantheria; Lukas 2012; Lukas 2013 |
| Saguinus_nigricollis | 0.580 | Harcourt 1995 | 2.672 | Harcourt 1995 |  | PA/PGA | Lukas 2012; Lukas 2013; Harcourt 1995; Moller 1988 |
| Saguinus_oedipus | 0.170 | Hayward 2011 | 2.700 | Hayward 2011 |  | M | Kenagy 1986; Lukas 2013; Lukas 2012 |
| Saimiri_sciureus | 0.505 | Anderson 2004 | 2.879 | Anderson 2004 |  | PA/PGA | Kenagy 1986; Moller 1988; Harcourt 1995 |
| Sarcophilus_harrisii | 0.716 | Anderson 2004 | 3.795 | Anderson 2004 |  | PA/PGA | Anderson 2004; Lukas 2012; Lukas 2013; Gooley 2018 |
| Scalopus_aquaticus | 0.342 | Hayward 2011 | 2.097 | Hayward 2011 |  |  |  |
| Sciurus_carolinensis | 0.714 | Hayward 2011 | 2.719 | Hayward 2011 |  | PA/PGA | Kenagy 1986; Waterman 2007 |
| Sciurus_vulgaris | 0.449 | Hayward 2011 | 2.544 | Hayward 2011 |  | PG | Waterman 2007 |
| Scotophilus_borbonicus | -0.854 | Ramm 2007 | 1.342 | Ramm 2007 |  |  |  |
| Scotophilus_heathii | -0.770 | Ramm 2007 | 1.574 | Ramm 2007 |  | PG | Hosken 2001 |
| Semnopithecus_entellus | 1.047 | Anderson 2004 | 4.232 | Anderson 2004 |  | PA/PGA | Anderson 2004; Soulsbury 2010; Harcourt 1995 |
| Sigmodon_hispidus | 0.238 | Hayward 2011 | 2.348 | Hayward 2011 |  |  |  |
| Sminthopsis_crassicaudata | -0.861 | Birkhead 1998 | 1.190 | Birkhead 1998 |  | PG | Rose 1997; Pantheria |
| Sminthopsis_macroura | -0.608 | Birkhead 1998 | 1.405 | Birkhead 1998 |  |  |  |
| Sminthopsis_virginiae | -0.599 | Birkhead 1998 | 1.491 | Birkhead 1998 |  |  |  |
| Sorex_araneus | -0.553 | Hayward 2011 | 1.124 | Hayward 2011 |  | PA/PGA | Soulsbury 2010 |
| Sorex_cinereus | -1.000 | Hayward 2011 | 0.724 | Hayward 2011 |  |  |  |
| Sorex_minutus | -1.000 | Hayward 2011 | 0.708 | Hayward 2011 |  |  |  |
| Sorex_palustris | -0.824 | Hayward 2011 | 1.246 | Hayward 2011 |  |  |  |
| Sotalia_fluviatilis | 2.679 | Hayward 2011 | 4.515 | Hayward 2011 |  |  |  |
| Sotalia_guianensis | 3.356 | Hayward 2011 | 4.958 | Hayward 2011 |  |  |  |
| Speothos_venaticus | 0.916 | Iossa 2008 | 3.869 | Iossa 2008 |  | M | Iossa 2008; Lukas 2013; Lukas 2012 |
| Spilogale_gracilis | 0.401 | Iossa 2008 | 2.728 | Iossa 2008 |  | PA/PGA | Iossa 2008; Ferguson 2004 |
| Spilogale_putorius | 0.732 | Iossa 2008 | 2.859 | Iossa 2008 |  | PA/PGA | Iossa 2008; Ferguson 2004 |
| Stenella_attenuata | 3.012 | Hayward 2011 | 4.906 | Hayward 2011 |  |  |  |
| Stenella_coeruleoalba | 2.653 | Dines 2015 | 5.193 | Dines 2015 |  |  |  |
| Stenella_frontalis | 3.234 | Dines 2015 | 5.155 | Dines 2015 |  |  |  |
| Stenella_longirostris | 3.227 | Dines 2015 | 4.871 | Dines 2015 |  | PA/PGA | Perrin 2003 |
| Steno_bredanensis | 3.477 | Dines 2015 | 5.190 | Dines 2015 |  |  |  |
| Stenonycteris_lanosus | 0.279 | Anderson 2004 | 2.000 | Anderson 2004 |  | PA/PGA | Anderson 2004 |
| Sturnira_bidens | -1.237 | Pitnick 2006 | 1.243 | Pitnick 2006 |  |  |  |
| Sturnira_lilium | -0.917 | Pitnick 2006 | 1.310 | Pitnick 2006 |  |  |  |
| Sundamys_muelleri | 0.872 | Breed 2000 | 2.602 | Breed 2000 |  |  |  |
| Suricata_suricatta | 0.114 | Soulsbury 2010 | 2.864 | Soulsbury 2010 |  | M | Iossa 2008; Soulsbury 2010; Pantheria; Lukas 2013; Lukas 2012 |
| Sus_scrofa | 2.857 | Anderson 2004 | 5.290 | Anderson 2004 |  | PG | Soulsbury 2010; Poteaux 2009 |
| Sylvicapra_grimmia | 1.327 | Anderson 2004 | 4.267 | Anderson 2004 |  | PG | Anderson 2004; Lukas 2012; Lukas 2013 |
| Sylvilagus_floridanus | 1.223 | Hayward 2011 | 3.058 | Hayward 2011 |  |  |  |
| Tachyglossus_aculeatus | 1.699 | Birkhead 1998 | 3.699 | Birkhead 1998 |  |  |  |
| Tadarida_aegyptiaca | -1.155 | Ramm 2007 | 1.207 | Ramm 2007 |  |  |  |
| Tadarida_brasiliensis | -1.301 | Ramm 2007 | 1.107 | Ramm 2007 |  | PA/PGA | Hosken 2001; Pitnick 2006 |
| Tamias_amoenus | -0.208 | Hayward 2011 | 1.629 | Hayward 2011 |  | PA/PGA | Soulsbury 2010; Waterman 2007 |
| Tamias_minimus | -0.143 | Hayward 2011 | 1.502 | Hayward 2011 |  |  |  |
| Tamias_townsendii | -0.086 | Hayward 2011 | 1.833 | Hayward 2011 |  |  |  |
| Tamiasciurus_hudsonicus | 0.465 | Soulsbury 2010 | 2.270 | Soulsbury 2010 |  | PA/PGA | Soulsbury 2010; Waterman 2007 |
| Taphozous_georgianus | -1.398 | Ramm 2007 | 1.477 | Ramm 2007 |  |  |  |
| Taphozous_longimanus | -1.114 | Pitnick 2006 | 1.556 | Pitnick 2006 |  | PG | Hosken 2001; Lukas 2013 |
| Tarsipes_rostratus | -0.438 | Birkhead 1998 | 0.949 | Birkhead 1998 |  |  |  |
| Tasmacetus_shepherdi | 3.130 | Dines 2015 | 6.329 | Dines 2015 |  |  |  |
| Tatera_vicina | 0.807 | Hayward 2011 | 2.303 | Hayward 2011 |  |  |  |
| Taurotragus_derbianus | 2.016 | Anderson 2004 | 5.646 | Anderson 2004 |  | PG | Anderson 2004; Lukas 2012; Lukas 2013 |
| Taxidea_taxus | 1.551 | Iossa 2008 | 3.895 | Iossa 2008 |  | PA/PGA | Iossa 2008; Ferguson 2004 |
| Tenrec_ecaudatus | 0.491 | Anderson 2004 | 3.301 | Anderson 2004 |  | PA/PGA | Anderson 2004 |
| Theropithecus_gelada | 1.332 | Hayward 2011 | 4.310 | Hayward 2011 |  | PA/PGA | Kenagy 1986; Moller 1988; Harcourt 1995 |
| Thomomys_bottae | 0.551 | Hayward 2011 | 2.279 | Hayward 2011 |  | PA/PGA | Kenagy 1986; Waterman 2007 |
| Thylogale_billardierii | 1.671 | Rose 1997 | 3.901 | Rose 1997 |  | PA/PGA | Rose 1997; Pantheria |
| Tokudaia_osimensis | -0.672 | Hayward 2011 | 1.949 | Hayward 2011 |  |  |  |
| Trachypithecus_cristatus | 0.799 | Hayward 2011 | 3.826 | Hayward 2011 |  | PG | Kenagy 1986 |
| Trachypithecus_francoisi | 0.600 | Anderson 2004 | 3.881 | Anderson 2004 |  | PG | Anderson 2004; Lukas 2012; Lukas 2013 |
| Trachypithecus_obscurus | 0.681 | Hayward 2011 | 3.872 | Hayward 2011 |  | PG | Kenagy 1986 |
| Tragelaphus_angasii | 1.583 | Anderson 2004 | 4.971 | Anderson 2004 |  | PA/PGA | Anderson 2004 |
| Tragelaphus_eurycerus | 1.723 | Anderson 2004 | 5.291 | Anderson 2004 |  | PG | Anderson 2004; Lukas 2012; Lukas 2013; Pantheria |
| Tragelaphus_strepsiceros | 1.964 | Anderson 2004 | 5.285 | Anderson 2004 |  | PA/PGA | Anderson 2004; Ginsberg 1990 |
| Trichechus_manatus | 2.789 | Reynolds 2004 | 5.650 | Reynolds 2004 |  | PA/PGA | Reynolds 2004 |
| Trichosurus_vulpecula | 0.602 | Anderson 2004 | 3.562 | Anderson 2004 |  | PA/PGA | Anderson 2004; Pantheria |
| Tursiops_aduncus | 3.312 | Dines 2015 | 5.362 | Dines 2015 |  | PA/PGA | Pantheria |
| Tursiops_truncatus | 3.090 | Dines 2015 | 5.393 | Dines 2015 |  | PA/PGA | Pantheria |
| Tylonycteris_pachypus | -1.155 | Ramm 2007 | 0.613 | Ramm 2007 |  | PA/PGA | Hosken 2001; Pitnick 2006 |
| Tylonycteris_robustula | -1.000 | Ramm 2007 | 0.924 | Ramm 2007 |  | PA/PGA | Hosken 2001; Pitnick 2006 |
| Uncia_uncia | 1.098 | Iossa 2008 | 4.699 | Iossa 2008 |  | PA/PGA | Iossa 2008 |
| Uranomys_ruddi | -0.458 | Breed 2000 | 1.602 | Breed 2000 |  |  |  |
| Urocitellus_beldingi | 0.544 | Soulsbury 2010 | 2.568 | Soulsbury 2010 |  | PG | Soulsbury 2010; Waterman 2007 |
| Urocyon_cinereoargenteus | 0.713 | Soulsbury 2010 | 3.568 | Soulsbury 2010 |  | M | Iossa 2008; Soulsbury 2010; Pantheria; Lukas 2013; Lukas 2012; Ferguson 2004 |
| Uroderma_bilobatum | -1.009 | Pitnick 2006 | 1.173 | Pitnick 2006 |  | PG | Hosken 2001; Lukas 2013 |
| Uromys_anak | 1.064 | Breed 2000 | 2.944 | Breed 2000 |  |  |  |
| Uromys_caudimaculatus | 0.857 | Hayward 2011 | 2.914 | Hayward 2011 |  |  |  |
| Ursus_americanus | 1.590 | Soulsbury 2010 | 5.010 | Soulsbury 2010 |  | PA/PGA | Iossa 2008; Soulsbury 2010; Pantheria; Ferguson 2004 |
| Ursus_arctos | 2.186 | Soulsbury 2010 | 5.494 | Soulsbury 2010 |  | PA/PGA | Iossa 2008; Soulsbury 2010; Pantheria; Ferguson 2004 |
| Ursus_maritimus | 2.119 | Iossa 2008 | 5.630 | Iossa 2008 |  | PA/PGA | Iossa 2008; Ferguson 2004 |
| Ursus_thibetanus | 1.824 | Iossa 2008 | 5.001 | Iossa 2008 |  | PA/PGA | Iossa 2008; Ferguson 2004 |
| Vampyrodes_caraccioli | -0.479 | Pitnick 2006 | 1.430 | Pitnick 2006 |  | PG | Hosken 2001; Lukas 2013 |
| Vampyrum_spectrum | -0.693 | Pitnick 2006 | 2.201 | Pitnick 2006 |  | M | Hosken 2001; Pitnick 2006; Pantheria; Lukas 2013; Lukas 2012 |
| Varecia_variegata | 0.420 | Anderson 2004 | 3.540 | Anderson 2004 |  | PG | Anderson 2004; Harcourt 1995; Lukas 2012; Lukas 2013 |
| Vombatus_ursinus | 1.265 | Birkhead 1998 | 4.603 | Birkhead 1998 |  |  |  |
| Vulpes_lagopus | 0.609 | Soulsbury 2010 | 3.681 | Soulsbury 2010 |  | M | Iossa 2008; Soulsbury 2010; Pantheria; Lukas 2013; Lukas 2012; Ferguson 2004 |
| Vulpes_vulpes | 0.954 | Hayward 2011 | 3.705 | Hayward 2011 |  | PG | Iossa 2008; Soulsbury 2010; Kenagy 1986; Lukas 2013; Lukas 2012; Ferguson 2004 |
| Vulpes_zerda | 0.736 | Iossa 2008 | 3.176 | Iossa 2008 |  | M | Iossa 2008; Pantheria; Ferguson 2004 |
| Wallabia_bicolor | 1.169 | Birkhead 1998 | 4.498 | Birkhead 1998 |  | PA/PGA | Rose 1997 |
| Zaglossus_bruijni | 1.908 | Birkhead 1998 | 3.839 | Birkhead 1998 |  |  |  |
| Ziphius_cavirostris | 3.903 | Macleod 2010 | 6.477 | Macleod 2010 |  |  |  |
| Zyzomys_argurus | -0.363 | Ramm 2007 | 1.653 | Ramm 2007 |  |  |  |
| Zyzomys_woodwardi | 0.238 | Ramm 2007 | 2.009 | Ramm 2007 |  |  |  |
| **Reptiles** | | | | | | | |
| Acrochordus_granulatus | 0.045 | Olsson 1998 | 2.000 | Macleod 2009 |  |  |  |
| Anolis_opalinus | -1.597 | Olsson 1998 | 0.342 | Macleod 2009 |  |  |  |
| Aspidoscelis_sexlineata | -1.018 | Olsson 1998 | 0.903 | Macleod 2009 |  |  |  |
| Aspidoscelis_tigris | -0.758 | Olsson 1998 | 1.255 | White 2006 |  |  |  |
| Calotes_versicolor | -0.677 | Olsson 1998 | 1.164 | Yu 2014 |  |  |  |
| Cerberus_rynchops | 0.152 | Olsson 1998 | 1.875 | Macleod 2009 |  |  |  |
| Cnemidophorus_ocellifer | -0.194 | Olsson 1998 | 1.877 | Macleod 2009 |  |  |  |
| Eulamprus_quoyii | -1.638 | Hayward 2011 | 1.362 | Hayward 2011 |  | PA/PGA | Noble 2013 |
| Hemidactylus_flaviviridis | -1.514 | Olsson 1998 | 0.477 | Macleod 2009 |  |  |  |
| Lacerta_agilis | -0.724 | Olsson 1998 | 0.924 | White 2006 |  | PA/PGA | Gullberg 1997 |
| Laticauda_colubrina | -0.222 | Olsson 1998 | 2.398 | Macleod 2009 |  | PA/PGA | Shetty 2002 |
| Micrurus_fulvius | -0.263 | Olsson 1998 | 1.477 | Macleod 2009 |  |  |  |
| Naja_naja | 1.377 | Olsson 1998 | 2.826 | Feldman 2016 |  |  |  |
| Notechis_scutatus | 1.062 | Olsson 1998 | 2.354 | Macleod 2009 |  |  |  |
| Orthriophis_taeniurus | -0.004 | Olsson 1998 | 1.882 | Macleod 2009 |  |  |  |
| Phrynosoma_cornutum | -0.009 | Olsson 1998 | 1.544 | White 2006 |  |  |  |
| Podarcis_siculus | -1.405 | Olsson 1998 | 0.879 | Macleod 2009 |  |  |  |
| Pseudonaja_textilis | 1.110 | Olsson 1998 | 2.647 | Macleod 2009 |  |  |  |
| Sceloporus_graciosus | -0.628 | Olsson 1998 | 0.699 | White 2006 |  | M/PG | Tinkle 1973 |
| Sceloporus_grammicus | -0.526 | Olsson 1998 | 0.784 | Macleod 2009 |  | M/PG | Degnan 2004 |
| Sceloporus_mucronatus | -0.681 | Olsson 1998 | 1.240 | Feldman 2016 |  |  |  |
| Sceloporus_occidentalis | -0.416 | Olsson 1998 | 1.005 | White 2006 |  | M/PG | Schall 1987 |
| Sceloporus_scalaris | -1.475 | Hayward 2011 | 0.851 | Hayward 2011 |  |  |  |
| Sceloporus_torquatus | -0.342 | Olsson 1998 | 1.488 | Macleod 2009 |  |  |  |
| Sceloporus_undulatus | -1.051 | Olsson 1998 | 0.580 | White 2006 |  | PA/PGA | Ferner 1974 |
| Tiliqua_rugosa | 0.121 | Olsson 1998 | 2.706 | White 2006 |  | M/PG | Olsson 1998 |
| Tropidurus_torquatus | -0.686 | Olsson 1998 | 1.680 | Macleod 2009 |  |  |  |
| Varanus_indicus | 0.429 | Olsson 1998 | 3.087 | Meiri 2010 |  |  |  |
| Varanus_olivaceus | 0.786 | Olsson 1998 | 3.640 | Bennett 2000 |  |  |  |
| Vipera_aspis | 0.052 | Olsson 1998 | 1.837 | Yu 2014 |  | PA/PGA | Olsson 1998 |
| Xantusia_riversiana | -0.734 | Olsson 1998 | 1.279 | White 2006 |  |  |  |
| Zootoca_vivipara | -1.408 | Olsson 1998 | 0.505 | Yu 2014 |  | PA/PGA | Richard 2012 |
