# Supplementary material for: Rapid decreases in relative testes mass among monogamous birds but not in other vertebrates
Source: Ecol Lett. 2019 Nov 21;23(2):283–92. doi: 10.1111/ele.13431 (PMC6973093; doi:10.1111/ele.13431)
Supplement: Supplementary file 2 [file ELE-23-283-s002.docx]

| **Table S2:** Shifts in the rate of testes size evolution identified across the vertebrate tree of life in all groups, including rate increases found in individual species. Each entry in the table represents a species or group of species that is evolving at a different rate compared to the clade from which they descended (Ancestral Clade). It is important to note that not all species in the reported clade may belong to the shift, only branches leading to the number of species reported as N. The median optimized rate (σ^2^_v_) for both the branch leading to the heritable rate shift and the ancestral lineage from which it descends is recorded. Although there is variation in the median rates attributed to a single ancestral clade (e.g. Neognathae), these do not differ in terms of their distribution (see Methods, Results, Figure 3). | | | | | |
| --- | --- | --- | --- | --- | --- |
| **Vertebrate Clade** | **Description** | **N** | **Log_10_ *σ^2^_v_*** | **Ancestral Clade** | ***Ancestral* Log_10_ *σ^2^_v_*** |
| *Fish* | | | | | |
|  | Order: Perciformes  (all members) | 26 | 0.003 | Background (Vertebrates) | 0.001 |
|  | Family: Cyprinidae  (most members) | 22 | 0.002 | Background (Vertebrates) | 0.001 |
|  | Pipefish, seahorses, and sea dragons (several genera) | 12 | 0.002 | Background (Vertebrates) | 0.001 |
|  | True minnows  (Genera: *Nocomis*, *Campostoma*) | 5 | 0.008 | Cyprinidae | 0.002 |
| *Frogs* | | | | | |
|  | Superfamily: Hyloidea +  Family: Myobatrachidae | 126 | 0.005 | Background (Vertebrates) | 0.001 |
|  | Families: Dicroglossidae and Rhacophoridae | 19 | 0.004 | Background (Vertebrates) | 0.001 |
|  | Genera: *Rana* and *Odorrana*  (all members) | 11 | 0.007 | Background (Vertebrates) | 0.001 |
|  | Genus: *Cyclorana*  (three members) | 3 | 0.112 | Bufonoidea | 0.040 |
|  | White-Lipped frogs  (Genus: *Leptodactylus*) | 3 | 0.030 | Bufonoidea | 0.005 |
|  | Family: Megophryidae  (all members) | 3 | 0.004 | Background (Vertebrates) | 0.001 |
|  |  |  |  |  |  |
| *Birds* | | | | | |
|  | Neognathae  (birds excl. tinamous and ratites) | 979 | 0.005 | Neognathae | 0.001 |
|  | Wading birds  (Order: Charardriiformes) | 18 | 0.016 | Neognathae | 0.005 |
|  | Kingfishers and bee-eaters (Order: Coraciiformes) | 8 | 0.022 | Neognathae | 0.005 |
|  | Crows and relatives  (Genus: *Corvus*) | 7 | 0.046 | Neognathae | 0.005 |
|  | Gerygones and peep-warblers (Genus: *Gerygone*) | 7 | 0.044 | Neognathae | 0.005 |
|  | Falcons  (genus: *Falco*) | 5 | 0.038 | Neognathae | 0.005 |
|  | Rufous-sided towhee complex (genus: *Pipilo*) | 4 | 0.045 | Neognathae | 0.005 |
|  | Two species of orpopendola (Genus: *Psarocolius*) | 2 | 0.100 | Neognathae | 0.017 |
|  | Two species of oriole  (genus: *Oriolus*) | 2 | 0.071 | Neognathae | 0.005 |
|  | Honeyeaters  (Genus: *Conopophila*) | 2 | 0.020 | Neognathae | 0.005 |
|  | Two species of friarbird  (Genus: *Philemon*) | 2 | 0.054 | Neognathae | 0.005 |
|  | Two species of manakin  (Genus *Pipra*) | 2 | 0.071 | Neognathae | 0.005 |
|  | Stiff-tailed ducks  (Genus: *Oxyura*) | 2 | 0.089 | Neognathae | 0.010 |
|  | Common redpoll  (*Carduelis flammea*) | 1 | 0.069 | Neognathae | 0.005 |
|  | Clamorous reed-warbler (*Acrocephalus stentoreus*) | 1 | 0.090 | Neognathae | 0.005 |
|  | Shining flycatcher  (*Myiagra alecto*) | 1 | 0.070 | Neognathae | 0.005 |
|  | Puerto Rican Vireo  (*Vireo latimeri*) | 1 | 0.075 | Neognathae | 0.005 |
|  | Yellow wattlebird  (*Anthochaera paradoxa*) | 1 | 0.117 | Neognathae | 0.022 |
|  | Yellow honeyeater (*Lichenostomus flavus*) | 1 | 0.062 | Neognathae | 0.005 |
|  | California gull  (*Larus californicus*) | 1 | 0.082 | Neognathae | 0.007 |
|  | Lesser scaup  (*Aythya affinis*) | 1 | 0.719 | Neognathae | 0.181 |
| *Mammals* | | | | | |
|  | All mammals excl. monotremes (Theria) | 618 | 0.003 | Background (Vertebrates) | 0.001 |
|  | Whales and dolphins  (Order: Cetacea) | 58 | 0.020 | Theria | 0.004 |
|  | The mouse genus *Pseudomys*  (all members) | 10 | 0.019 | Theria | 0.005 |
|  | Rousettines + African megabats  (Family: Pteropodidae) | 7 | 0.024 | Theria | 0.004 |
|  | Mouse-eared bats  (Genus: *Myotis*) | 4 | 0.023 | Theria | 0.004 |
|  | Hopping mice  (Genus: *Notomys*)* | 4 | 0.061 | Theria | 0.004 |
|  | Sheep  (Genus: *Ovis*) | 3 | 0.050 | Theria | 0.005 |
|  | Asses and zebras  (Genus: *Equus*) | 3 | 0.058 | Theria | 0.013 |
|  | The mouse genus *Mus*  (three members) | 3 | 0.015 | Theria | 0.004 |
|  | Fawn hopping mouse  (*Notomys* *cervinus*)** | 1 | 0.005 | Notomys | 0.061 |
|  | California mouse  (*Peromyscus californicus*) | 1 | 0.098 | Theria | 0.010 |
|  | Yellow baboon  (*Papio cynocephalus*) | 1 | 0.055 | Theria | 0.018 |
| *Reptiles* | | | | | |
|  | Spiny lizards  (Genus: *Sceloporus*) | 7 | 0.001 | Background (Vertebrates) | 0.001 |
|  | Elapid snakes  (Family: Elapidae) | 5 | 0.004 | Background (Vertebrates) | 0.001 |
| *This is the only rate shift identified that represents a “mean shift”; i.e. the branch leading to this group of taxa has an elevated rate, but the branches within the clade return to the rate of evolution observed within the ancestral clade.  ** All rate shifts with the exception of this branch, leading to *Notomys cervinus* are rate increases. The branch leading to this taxa is evolving at a slower rate compared to its ancestor (in this case, both the mean shift leading to all *Notomys* species, and the rate acting across all Therian mammals). | | | | | |
