# Supplementary material for: Rapid decreases in relative testes mass among monogamous birds but not in other vertebrates
Source: Ecol Lett. 2019 Nov 21;23(2):283–92. doi: 10.1111/ele.13431 (PMC6973093; doi:10.1111/ele.13431)
Supplement: Supplementary file 3 [file ELE-23-283-s003.docx]

# Supplementary Text

## Testes mass and body mass data

We collected testes mass and body mass for vertebrate species from the literature, prioritizing data from sources that contained data for multiple species, and supplementing data for individual species. To avoid conflicts among datasets, we enforced a standardized protocol to ensure a single measurement for each species.

Firstly, we preferred sources that directly measured testes mass and male body mass of individuals. Where a source was a literature compilation, we preferred those that attempted to obtain measurements from single individuals or, failing that, attempted to obtain testes and body mass of individuals from the same geographic regions (e.g. Kenagy & Trombulak 1986). Details on how this was achieved differs for individual datasets and so refer to the original sources for more information. Where single values represent multiple individuals or populations, we preferred mean values to maximums (only 29 species total come from a dataset that reports maximal testes mass). We placed low priority on values collected using data extraction software from images (e.g. Fitzpatrick et al. 2012) owing to possible user-associated error. Finally, if multiple sources fit the above criteria, we prioritized more recent datasets. We placed any ambiguous data sources at the end of the priority list. Where multiple datasets remained, we prioritized the dataset with the largest sample size (note that whilst using weighted means is an option, not all datasets provide sample size and using only a single source per taxa allowed us to apply a single protocol across all species and sources). Any sources that only contained testes mass (and no body mass) were included as the lowest priority, and were supplemented with body mass data from additional sources (N = 109 species in our final dataset, mostly within fish).

Our complete testes and body mass dataset comprised measurements for 1913 vertebrate species (91 fish, 186 frogs, 983 birds, 621 mammals, and 32 reptiles) collated from 92 different published sources – all data and references are recorded in Table S1.

We manually modified two values identified as errors as follows. Firstly, the California mouse (*Peromyscus californicus*) is erroneous in our preferred source (Soulsbury 2010), where it is reported to have 9.6g testes mass. This results from an incorrect unit conversion in the original cited source (Nelson et al. 1995), which we correct in our dataset (0.0096g). Secondly, the body mass of the laughing dove (*Streptopelia senegalensis*) was identified by Peter Dunn (personal communication) to be incorrect in our preferred source (Calhim & Birkhead 2007) which reports this species to be 584g. This large body mass also falls well outside the mass range of 66.8-101g reported in Dunning (2007). We adjusted the body mass of this species to 100.1g as reported in other published sources (Dunn et al. 2001; Pitcher et al. 2005).

## Matching species names

We matched species to the vertebrate portion of the time tree of life (Hedges et al. 2015), checking for spellings and synonymy using major taxonomic resources (Species Survival Commision 2001; AmphibiaWeb 2005; Lepage 2009; Froese & Pauly 2012; BirdLife Taxonomic Working Group 2015; Roskov et al. 2016). To maximize our sample size, we incorporated species that did not match by genus matching. Where a species with testes mass data is the sole member of its genus found in the data, we substituted the data for that species with another member of the genus found in the phylogeny. We only did this where genera comprised monophyletic clades, and where it did not alter existing relationships among the species included in our dataset (Table S1).

## Testes mass – Body mass allometry

We find differences in the slope of the relationship between testes mass and body mass for each of the major vertebrate clades, with the exception of reptiles (Figure 2). This is in line with recent analyses that find differences among animal groups (Hayward & Gillooly 2011), but in contrast with analyses that suggest that a single slope could explain most variation amongst amniotes (MacLeod & MacLeod 2009). This analysis suggested that there are differences in testes mass allometry for species of different body masses (MacLeod & MacLeod 2009; MacLeod 2014). That is, there is a single relationship across all species that differs not among taxonomic groups but instead over the range of observed body masses.

If the testes mass of large-, intermediate-, and small-bodied species (however these might be defined) differ in relative investment into testes mass independently and regardless of ancestry (MacLeod & MacLeod 2009; MacLeod 2014) then we should find a cubic relationship between testes mass and body mass (MacLeod 2014). To test this, we repeated our variable rates models but included an additional parameter that estimates a cubic curvature in the relationship. There is no support for the inclusion of a cubic parameter after considering the different testes mass – body mass relationships among the major vertebrate clades (P*_[x]_* = 0.126) that are supported by our main analyses.

## Social mating system data

We collected social mating system data for species with testes mass and body mass data from the literature as a three-state categorical variable: monogamy, polygyny, and polyandry/polygynandry. We used the datasets from which we collected testes mass data in additional to several other compilations from the literature (see Table S1 for full reference list). We treated all sources of mating system data as equally valid; species-level classifications were therefore taken as that of the highest level of complexity (in increasing order from monogamy, through polygyny to polyandry/polygynandry). For example, a species classified as polyandrous in one dataset and polygynous in another would be defined here as “polyandrous/polygynandrous” whereas species classified as monogamous and polygynous in different datasets would be considered here as polygynous. In the absence of other information, several sources were excluded owing to a lack of clearly defined social mating system: e.g those which simply classify species as “non-monogamous” (Lukas & Clutton-Brock 2012; Lukas & Clutton-Brock 2013) or those which only provide details on female mating system (polyandry or monogamy/polygyny) (Anderson et al. 2004). To avoid discarding data, where possible, we combined these ambiguous sources to clearly deduce a mating system for a species that would otherwise lack classification: for example, in two different sources, *Rusa unicolor* (Sambar deer) is classified as female single-partner (Anderson et al. 2004) and as non-monogamous (Lukas & Clutton-Brock 2013). Combining these two mating systems gives a mating system of polyandrous, and so this species was classified as having a social mating system of polyandry/polygynandry.

Our complete testes, body mass, and social mating dataset comprised measurements for 63 fish, 169 frogs, 845 birds, and 358 mammals, collated from 77 different published sources – all data and references can be found in Table S1. Where required, additional details pertaining to collection of social mating system data for the major vertebrate groups can be found below.

### Frogs

For frogs, most available mating system data comes from binary classifications (monandry or polyandry) and so for these species, we simplified our mating system categorization to these two categories only. There are still clear expectations for monandrous vs polyandrous testes masses. In (Byrne et al. (2002)) frogs are given a “sperm competition rank” where species ranked 0-3 are less likely to be multi-male than single male and 4 is polyandry. We define species with a sperm competition rank of 4 as polyandrous and all others as monandrous.

### Fish

Group spawning is defined as promiscuous mating, whereas pair spawning tends to only involve a single male and single female (Johnston & Page 1992). We therefore define three “pair spawning” fish are defined as monogamous in the absence of further details*: Nocomis asper*, *N. leptocephalus*, and *N. micropogon* (Pyron *et al.* 2013). Although pair-spawning fish commonly have reproductive tactics and sneak-mating (Neff 2001) which make social mating systems difficult to identify, our results are identical if these three species are excluded from the analysis.

Note that our sample sizes for monogamous and polygynous fish were too small to analyse separately (N = 15 and N = 8 respectively). In our analysis that estimated a separate slope in the relationship between testes mass and path-wise rate for different mating systems, we therefore combined these two mating systems into a single category. As the slope is non-significant (see results), we estimated separate intercepts for all three mating systems – although we have very few polygynous species, it should still possible to estimate a mean (i.e., intercept difference).

### Birds

In birds, 90 species are classified as belonging to a cooperative social mating system. Most cooperative breeders consist of a monogamous mating pair with additional related helpers (Hartley & Davies 1994). However, several cooperative breeders are known to have high levels of polyandry e.g. (Hartley & Davies 1994; Chao 1997). In many cases, cooperative mating systems were denoted simply as “cooperative” and thus it was unclear of the composition of social groups. Rather than include a category with mixed composition of social groups, we excluded these species from the analyses.

We identify 55 species as belonging to “lekking/promiscuous” social mating systems from several sources. Although it is suggested that sperm competition acts similarly within lekking species as it does for monogamous species (Garamszegi et al. 2005), we made no assumptions and instead defined social mating systems independently for these species (see Table S1 for sources). We excluded 12 lekking species for which we could not obtain more specific social mating system data; however, results do not differ if we consider these 12 species as either polygynous or polyandrous/polygynandrous.

### Reptiles

To our knowledge, there is no compilation of mating system data readily available for squamates – at least not one that overlaps with our testes mass data. For these species, we collected data from individual sources (N = 12, see Table S1). However, we did not perform additional tests on these data owing to too small of a sample size.

**Cross-validating the trend in monogamous birds**

We ensure the consistency of our results by performing 100 independent cross-validation tests. To do this, we divide our dataset in two by randomly sampling 50% of all monogamous bird species. We then repeat our trends analysis in both halves of the dataset, estimating the relationship between testes mass and path-wise rate. We find that the significant negative relationship stands in both halves of 98% of all randomly re-sampled datasets (Figure S1). We are therefore confident in the robustness of our results across monogamous birds.


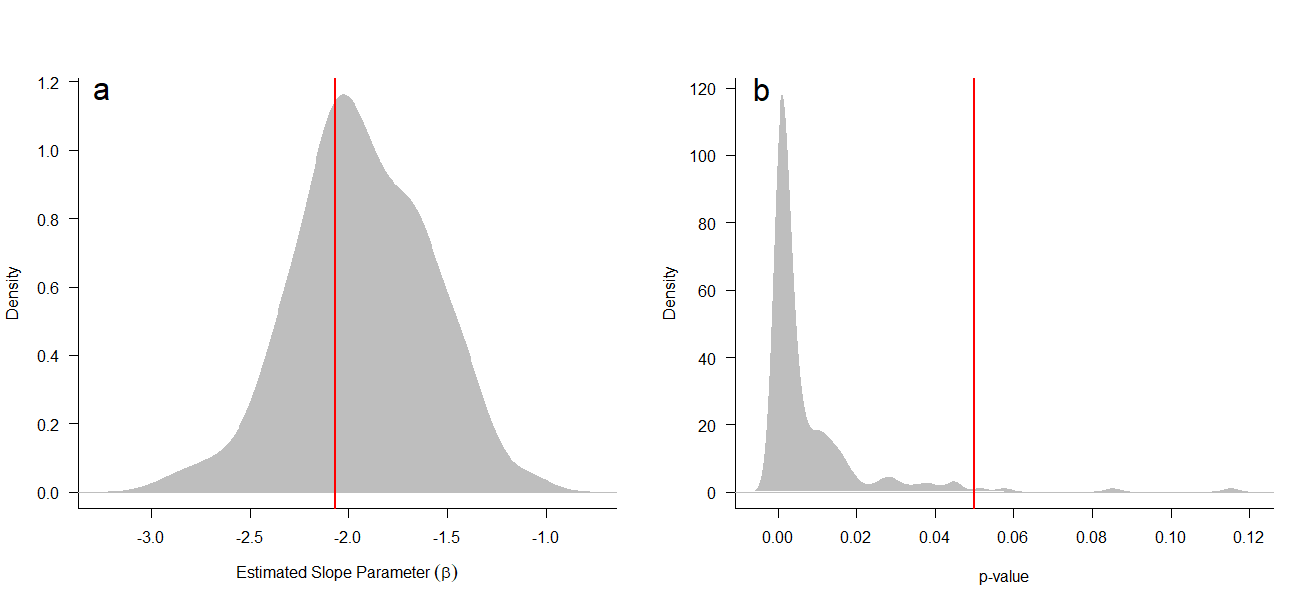


**Figure S1** – The results of 100 cross-validation tests. The distribution of estimated β parameters (the slope value for the relationship between testes mass and path-wise rate) and its significance (p-value) are shown in (a) and (b) respectively. Values are shown for two datasets for each cross-validation test, generated by randomly re-sampling 50% of monogamous bird species (i.e. 200 models are presented in this figure). For reference, vertical lines are drawn at the slope value from the model in our main text that estimates a relationship across all monogamous birds in (a) and at p=0.05 in (b).

**Running Variable Rates Regressions in BayesTraits V3**

BayesTraits is a command-line driven program. The variable rates regression model requires the specification of both a tree file (this must be in nexus format) and a tab-delimited text file containing the data (full details on the structure of this data file and also the nexus phylogenetic tree file format can be found on pgs. 8-10 of the BayesTraits manual, available at: <http://www.evolution.rdg.ac.uk/BayesTraitsV3.0.1/Files/BayesTraitsV3.Manual.pdf>).

BayesTraitsV3.exe Tree.nexus Data.txt

After specifying a phylogenetic tree and dataset as above, options for the model choice are prompted on screen from which the user can select their desired option. In this case, we would see the options as shown below, and select option 9 as we are interested in running the fast-likelihood regression model (outlined on p. 8 of the manual with full instructions on p.42):

1) MultiState

4) Continuous: Random Walk (Model A)

5) Continuous: Directional (Model B)

6) Continuous: Regression

7) Independent Contrast

8) Independent Contrast: Correlation

9) Independent Contrast: Regression

The program then prompts the user to specify whether they would prefer to use maximum likelihood or MCMC. In this case, we are interested in MCMC and so select option 2.

Please select the analysis method to use.

1) Maximum Likelihood.

2) MCMC

Optional model specifications are then entered manually according to the desired construct of the model. In this case, we want the variable rates model (p. 51- 52 of the manual), remove the first 500 thousand iterations as burn-in, sample 1 billion and 500 thousand iterations keeping only 500 thousandth sample (details of these options are on pages 16-17 of the manual). We want to estimate stepping stones using a total of 200 stones with 1 million iterations per stone (pg. 14). We then want to run this analysis (pgs. 8-10).

varrates

burnin 500000

iterations 1000500000

sample 500000

stones 200 1000000

run

A full list of all optional commands is provided in the manual with detailed descriptions of their function and specification.

**References**

1. AmphibiaWeb (2005). AmphibiaWeb: Information on amphibian biology and conservation. Available at: <http://amphibiaweb.org/> Last accessed March 2016.

2. Anderson, M.J., Nyholt, J. & Dixson, A.F. (2004). Sperm competition affects the structure of the mammalian vas deferens. *J Zool*, 264, 97-103.

3. BirdLife Taxonomic Working Group (2015). BirdLife Taxonomic Checklist v8.0. Available at: <http://www.birdlife.org/datazone/info/taxonomy> Last accessed March 2016.

4. Byrne, P.G., Roberts, J.D. & Simmons, L.W. (2002). Sperm competition selects for increased testes mass in Australian frogs. *J Evol Biol*, 15, 347-355.

5. Calhim, S. & Birkhead, T.R. (2007). Testes size in birds: quality versus quantity - assumptions, errors, and estimates. *Behav Ecol*, 18, 271-275.

6. Chao, L. (1997). Evolution of polyandry in a communal breeding system. *Behav Ecol*, 8, 668-674.

7. Dunn, P.O., Whittingham, L.A. & Pitcher, T.E. (2001). Mating systems, sperm competition, and the evolution of sexual dimorphism in birds. *Evolution*, 55, 161-175.

8. Dunning, J.B. (2007). *CRC handbook of avian body masses*. 2nd edn. Taylor & Francis, Florida, USA.

9. Fitzpatrick, J.L., Almbro, M., Gonzalez‐Voyer, A., Kolm, N. & Simmons, L.W. (2012). Male contest competition and the coevolution of weaponry and testes in pinnipeds. *Evolution*, 66, 3595-3604.

10. Froese, R. & Pauly, D. (2012). FishBase. Available at: <http://www.fishbase.org> Last accessed March 2016.

11. Garamszegi, L.Z., Eens, M., Hurtrez-Boussès, S. & Møller, A.P. (2005). Testosterone, testes size, and **mating success in birds: a comparative study. *Horm Behav*, 47, 389-409.**

12. Hartley, I.R. & Davies, N.B. (1994). Limits to Cooperative Polyandry in Birds. *Proc R Soc Lond [Biol]*, 257, 67-73.

13. Hayward, A. & Gillooly, J.F. (2011). The cost of sex: Quantifying energetic investment in gamete production by males and females. *PLoS ONE*, 6, e16557.

14. Hedges, S.B., Marin, J., Suleski, M., Paymer, M. & Kumar, S. (2015). Tree of life reveals clock-like speciation and diversification. *Mol Biol Evol*, 32, 835-845.

15. Johnston, C.E. & Page, L.M. (1992). The evolution of complex reproductive strategies in North American minnows (Cyprinidae). *Systematics, historical ecology, and North American freshwater fishes Stanford University Press, Stanford, California*, 600-621.

16. Kenagy, G. & Trombulak, S.C. (1986). Size and function of mammalian testes in relation to body size. *J Mammal*, 67, 1-22.

17. Lepage, D. (2009). Avibase–the world bird database. Available at: <http://avibase.bsc-eoc.org> Last accessed March 2016.

18. Lukas, D. & Clutton-Brock, T. (2012). Cooperative breeding and monogamy in mammalian societies. *Proc R Soc Lond B Biol Sci*, 279, 2151-2156.

19. Lukas, D. & Clutton-Brock, T.H. (2013). The evolution of social monogamy in mammals. *Science*, 341, 526-530.

20. MacLeod, C.D. (2014). Exploring and explaining complex allometric relationships: A case study on amniote testes mass allometry. *Systems*, 2, 379-392.

21. MacLeod, C.D. & MacLeod, R. (2009). The relationship between body mass and relative investment in testes mass in amniotes and other vertebrates. *Oikos*, 118, 903-916.

22. Neff, B.D. (2001). Alternative reproductive tactics and sexual selection. *Trends Ecol Evol*, 16, 669.

23. Nelson, R.J., Gubernick, D.J. & Blom, J.M. (1995). Influence of photoperiod, green food, and water availability on reproduction in male California mice (*Peromyscus californicus*). *Physiol Behav*, 57, 1175-1180.

24. Pitcher, T., Dunn, P. & Whittingham, L. (2005). Sperm competition and the evolution of testes size in birds. *J Evol Biol*, 18, 557-567.

25. Pyron, M., Pitcher, T. & Jacquemin, S. (2013). Evolution of mating systems and sexual size dimorphism in North American cyprinids. *Behav Ecol Sociobiol*, 67, 747-756.

26. Roskov, Y., Abucay, L., Orrell, T., Nicolson, D., Flann, C., Bailly, N. *et al.* (2016). Species 2000 & ITIS Catalogue of Life, 2016 Annual Checklist. Naturalis Leiden, the Netherlands.

27. Soulsbury, C.D. (2010). Genetic patterns of paternity and testes size in mammals. *PLoS ONE*, 5, e9581.

28. Species Survival Commision (2001). IUCN red list categories and criteria: version 3.1. *Prepared by the IUCN Species Survival Commission*.
